# Nanoscopic Characterization of the Thermal Phase Behavior of Random Poly(ethylene glycol-glycidyl methyl ether) Copolymers (rPEGs)

**DOI:** 10.1021/acs.macromol.6c01114

**Published:** 2026-07-16

**Authors:** Dominik Schulz, Elena Berger-Nicoletti, Johannes Wingert, Julian Gatzki, Haleh Hashemi Haeri, Dariush Hinderberger, Holger Frey

**Affiliations:** † Department of Chemistry, 9182 Johannes Gutenberg-University Mainz, Duesbergweg 10-14, 55128 Mainz, Germany; ‡ Institute of Chemistry, 9176 Martin-Luther-Universität Halle-Wittenberg, Von-Danckelmann-Platz 4, 06120 Halle, Germany

## Abstract

PEGylation—linking of poly­(ethylene glycol) (PEG) to a nanocarrier or active pharmaceutical ingredient to achieve stealth properties—is a key technology of nanomedicine. However, PEG has been shown to induce the formation of anti-PEG antibodies, motivating the search for alternative polymers for conjugation with nanocarriers. Recently, the concept of randomized PEG (rPEG), random copolymers of ethylene oxide and glycidyl methyl ether (GME), was introduced. These polymers are structural isomers of PEG with potential for manifold biomedical applications. To investigate their phase behavior in aqueous solution, a series of rPEGs with systematically varied monomer ratios were synthesized. Turbidimetric determination of the cloud point temperatures (***T***_**cp**_) was applied to study macroscopic lower critical solution temperature behavior. Paralleling these measurements, local nanophase separation was investigated by electron paramagnetic resonance spectroscopy (EPR) using amphiphilic spin probes to address two key questions: (i) polymer hydration at nanoscopic level and (ii) to determine the temperature at which the onset of the collapse of the polymer chains occurs. The results reveal no phase separation below 96 °C for copolymers of a GME content of 35 mol % and less (26 and 0 mol % GME), while for GME content exceeding 40 mol %, cloud points of 70 °C–96 °C were observed. Comparison of both methods shows good accordance between the cloud points determined by turbidimetry and EPR with the exception of poly­(glycidyl methyl ether) (PGME), for which EPR showed a lower ***T***_**cp**_ by 5–10 °C. Combining the results with literature data, a model could be established that gives insight into the nanoscopic processes and allows for an approximation of the macroscopic cloud point temperatures in dependence of the GME content. Excellent aqueous solubility of all samples could be demonstrated in the physiological temperature range, satisfying the requirements for biomedical applications.

## Graphical abstract

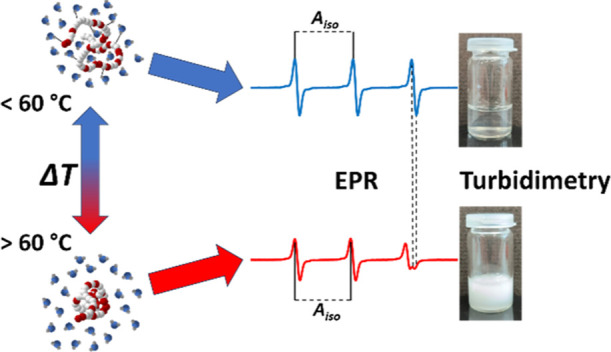

## Introduction

When aiming at pharmaceutical or medical applications, the behavior of polymers in aqueous solution is of particular importance.^1,2^ Consequently, key polymers like poly­(ethylene glycol) that possess excellent aqueous solubility are of special interest for the medical field.^3,4^ This feature together with its very low toxicity has led to an immense variety of uses for PEG in the fields of pharmaceutical technology and nanomedicine,^5–7^ cosmetics,^8^ and also as a food additive.^9^ The high aqueous solubility of PEG is due to the position of the oxygen atoms in the polymer backbone. With 2.88 Å, the oxygen–oxygen distance is very similar to the distance of the hydrogen atoms in liquid water at 25 °C (2.85 Å).^10^ Therefore, PEG can be integrated into a water lattice at room temperature by forming a hydration shell around its backbone.^10,11^

However, this behavior does not hold true for all polyethers. Introduction of hydrophobic side chains into the backbone of the polyether, for instance, by copolymerization of propylene oxide, leads to significantly reduced solubility in aqueous solution because the formation of the hydration shell of the polymer chain is sterically disabled.^11^ The solubility of polyethers in aqueous solution can therefore be tailored by either variation of the epoxide monomer side chain^12^ or copolymerization of hydrophobic and hydrophilic monomers.^13,14^

Most commonly, the aqueous solubility of polymers is quantified by observing the dependence of their solubility on the temperature of the solution.^15,16^ The formation of the hydration shell of a polyether is enthalpically favored but entropically disfavored. For low temperatures, the enthalpic term of the Gibbs–Helmholtz equation will outbalance the entropic term, leading to negative Gibbs free energy Δ ***G***_**mix**_ and therefore a solution of the polymer in water. With increasing temperature, the entropic term ***T*** Δ ***S***_**mix**_ will increase in value until Δ ***G***_**mix**_ becomes positive, and the polymer undergoes a coil to globule transition. This leads to a phase separation caused by “shedding” of the hydration shell and eventually results in precipitation of the polymer.^17,18^ Macroscopically, this can be observed by turbidity of the mixture. For a defined concentration of the polymer in a solvent like water, a cloud point temperature ***T***_**cp**_ can be determined by measuring the transmittance of a polymer/solvent mixture depending on its temperature. The lowest possible cloud point that can be achieved when varying the molar ratio of the polymer and solvent in the mixture is called the lower critical solution temperature (LCST). Below this temperature, the polymer and solvent are completely miscible at any given ratio.^19^ The turbidity of a solution, however, is a macroscopically observable effect. To observe the coil to globule transition on a molecular level, other methods such as NMR spectroscopy and dynamic light scattering (DLS) can be employed. Valuable information at the nanoscopic level can be obtained via electron paramagnetic resonance (EPR) spectroscopy. This method affords comprehensive data regarding the hydration state of a polymer and local density of the polymer-rich phase. Furthermore, transition temperatures well below the macroscopically detected values are perceptible that mark the onset of the transition. The strength of this technique in elucidating such aspects has been shown in several publications by the authors and other researchers^20–28^ (Figure 1). TEMPO (2,2,6,6)-tetramethylpiperidinyloxyl is a stable aminoxyl radical that is widely used in EPR studies. Due to its moderate solubility in water and high solubility in various organic solvents (*P*_ow_ ∼ 90),^29,30^ it serves as an amphiphilic spin probe, suitable for monitoring both water-rich (hydrophilic) and polymer-rich (hydrophobic) nanoscopic regions. Moreover, rotational dynamics of the spin probe can be monitored through the defined correlation time as a single measure (τ_c_, in nano- to picosecond, ns–ps, regime), which provides another hint regarding the presence of a spin probe in different media (like collapsed or aggregated).

**Figure 1:**
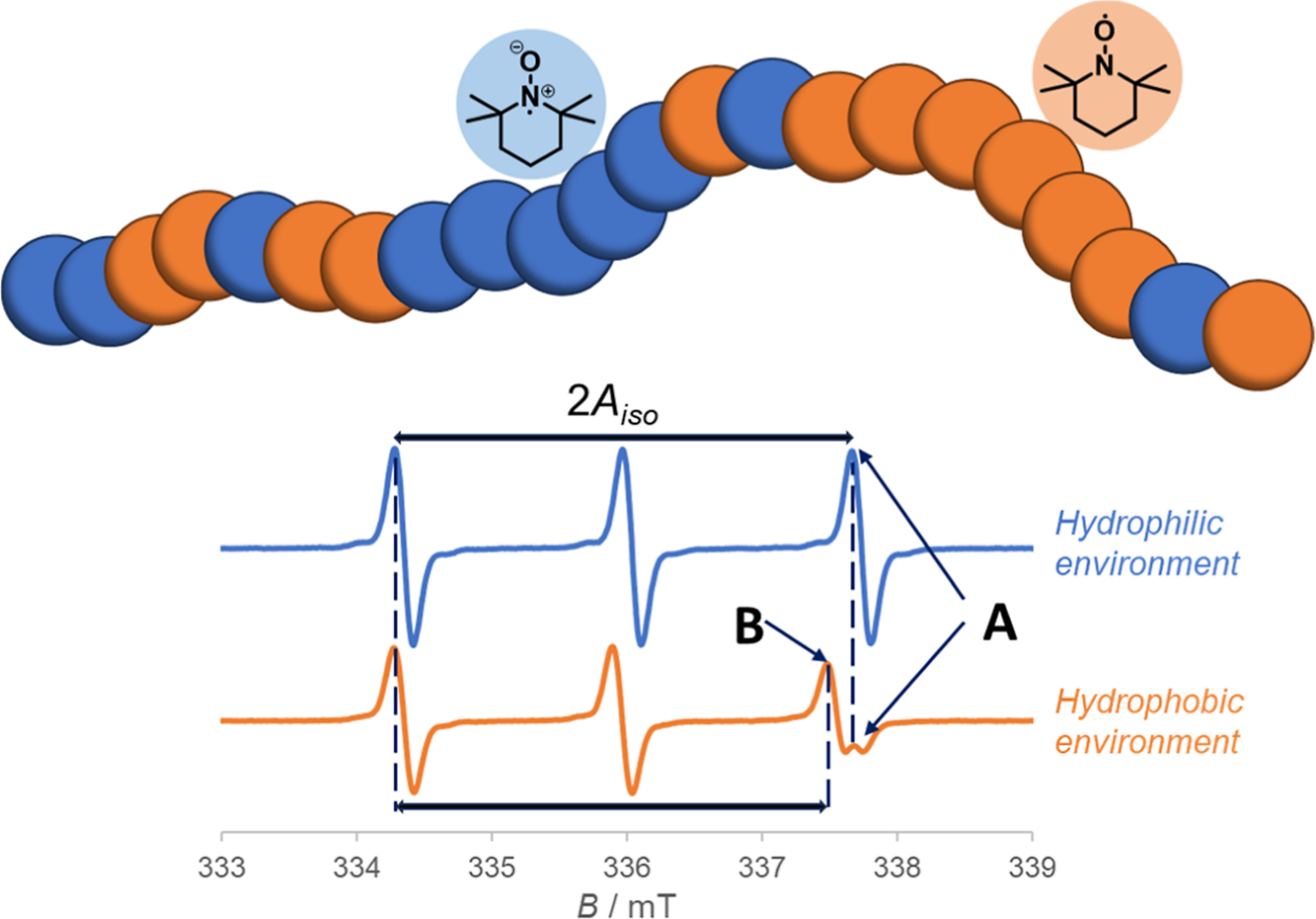
Polymer chain with more polar/hydrophilic (blue) and less polar/more hydrophobic (orange) regions and the mesomeric structures of TEMPO molecules predominantly located in those regions. The change in the polarity of the surrounding medium is best reflected in the high-field peak position (∼337.5 mT), where the two species are clearly discernible.

The electronic structure of TEMPO can be described as being between two mesomeric structures (Figure 1). While located in a polar, water-rich environment, the slightly more prevalent resonance structure has a higher spin density located on the nitrogen atom. If TEMPO is present in a water-depleted, more polymer-rich region, the other resonance structure is more populated, in which the oxygen atom bears higher spin densities. This change in spin density localization can be observed in two ways: either as a variation of the ***g***_***xx***_ element of ***g***-tensor to higher values, or as a shift in the so-called ^14^N-hyperfine coupling constant (***A***_**iso**_), a measure of which for a nitroxide is the hyperfine splitting that can be read out of from isotropic spectra as roughly half-distance between two outermost lines (see Figure 1).^23,28,31^ Therefore, collapse of the hydration shell in little hydrated regions of the copolymers can be spectroscopically observed by the impact on interacting spin probes. While the scattering of visible light for turbidity measurements requires particle sizes of several hundred nanometers, spin probes can already detect localized chain collapse behavior in the range of a few nanometers.^22,28,32^

We recently developed the concept of randomized PEG (rPEG).^33^ rPEGs are random copolymers of ethylene oxide (EO) and glycidyl methyl ether (GME) synthesized via statistical anionic ring-opening copolymerization (AROP). GME possesses a methyl­(methoxy) side chain, making it a structural isomer of two EO molecules. Consequently, this property translates to the rPEGs, which are structural isomers of PEG, regardless of their EO/GME composition (Figure 2). The methyl­(methoxy) side chains of GME units lower the overall hydrophilicity of the copolymer compared to PEG to a certain extent. Thanks to the ideally random nature of this copolymerization, the side chains are distributed randomly at the polyether backbone. By avoiding the regularity of the monomer sequence in the backbone of PEG, rPEGs can evade immune reactions caused by anti-PEG antibodies. We recently reported an in vitro study,^34^ in which we investigated antibody binding to rPEGs with varying molar GME contents. We observed that a GME content above 25 mol % significantly reduces the binding of backbone-specific anti-PEG antibodies. At approximately 50 mol % GME content, no antibody binding could be observed anymore, qualifying rPEGs as a potential PEGylation alternative to address the rising concerns regarding the prevalence of anti-PEG antibodies in the population. Furthermore, since rPEGs with a molar content of more than 26 mol % GME are amorphous at room temperature—contrary to the semicrystalline PEG—these polymers can be used to create unprecedented polyether materials for which crystallinity is undesired but a PEG-like structure is mandatory, e.g., in areas such as membrane technology^35^ or battery development.^36^ Since excellent aqueous solubility is a crucial requirement for any medical application, the aim of this work was to investigate if the incorporation of GME into the polyether backbone would lead to a change in the nanoscopic chain collapse behavior and subsequent significant decrease in the cloud point temperatures of the copolymers. Furthermore, the temperature dependence of local hydration on a nanometer scale has been studied.

**Figure 2:**
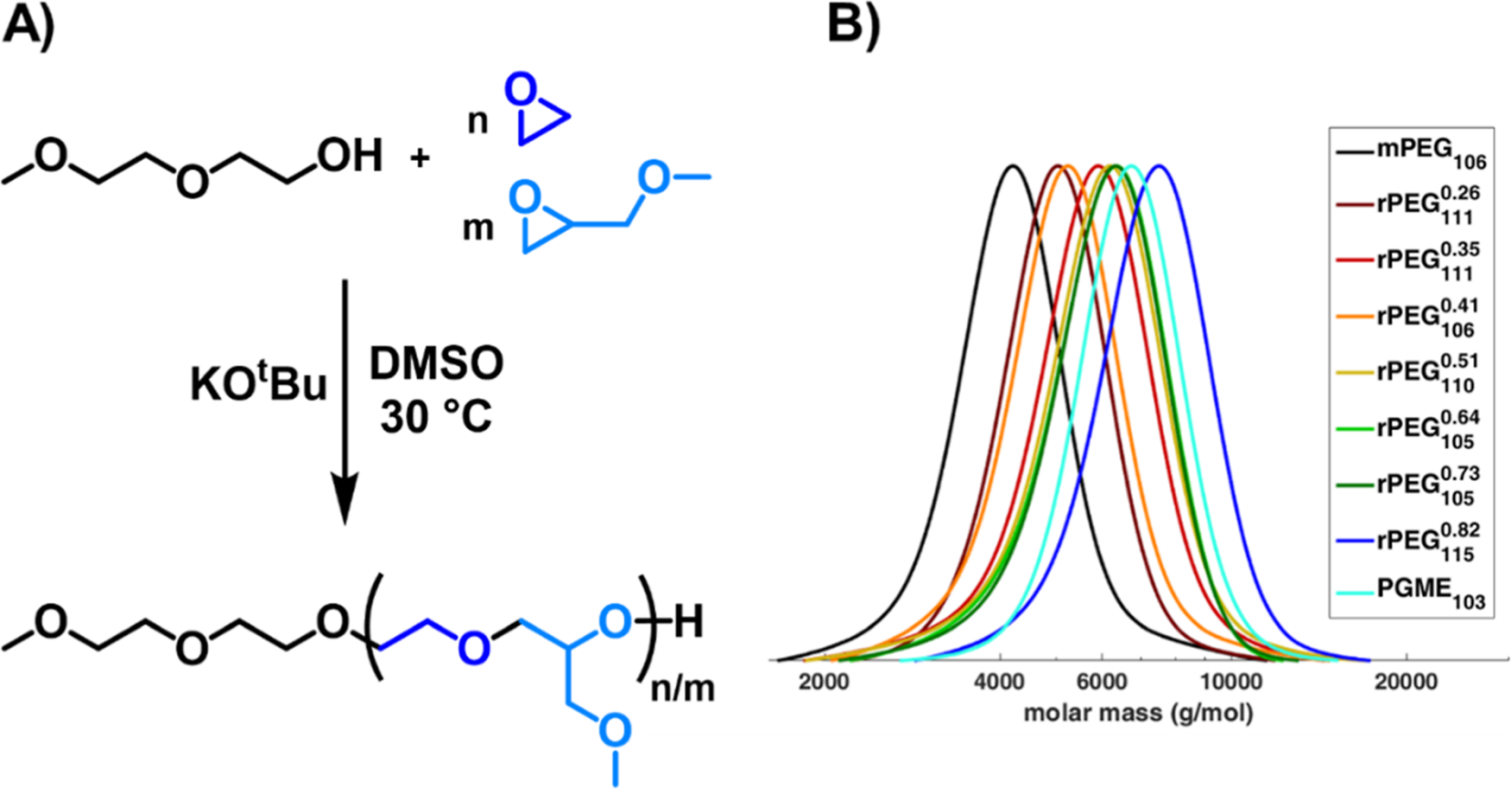
(A) General reaction scheme for the copolymerization of EO (dark blue) and GME (light blue). Diethylene glycol monomethyl ether (black) was used as an initiator in all (co)­polymerizations. (B) SEC curves of the synthesized copolymers.

## Results and Discussion

### Synthesis and Characterization of rPEG Copolymers

rPEGs of varying compositions have recently been shown to possess low cell toxicity and high aqueous solubility at physiological temperatures.^33^ The aim of this investigation is to gain detailed insight into the thermoresponsive behavior of rPEGs in aqueous solution from the nanoscale up to the macroscale. For this purpose, turbidimetry measurements were performed to assess the cloud point temperatures of rPEGs with respect to the molar GME content. The macroscopic cloud point was compared to EPR spin probe investigations, intrigued by the question of whether localized hydrophobic regions of the copolymers can collapse before the phase separation can be detected by clouding of the solution. Understanding this behavior will give further insight into the hydrophilicity of rPEGs and shed light on the temperature dependence of the nanoscopic hydration shell.

### Monomer Synthesis

GME is a common epoxide monomer that is commercially available from several vendors. Large-scale synthesis and laboratory synthesis are based on the methoxylation of epichlorohydrin (ECH).^37,38^ A significant disadvantage of this method is that in all commercial GME batches, residuals of unreacted ECH are still present (see Supporting Information, Figure S1), which causes undesired chain termination in anionic ring-opening polymerization. Therefore, in several works, PGME could only be synthesized with low molecular weights and broad molecular weight distributions via classic AROP.^39^ However, using specialized techniques like the monomer-activated anionic ring-opening polymerization (MAROP), high molecular weights were achieved.^40^ Since MAROP requires toxic additives that are not approved by the Food and Drug Administration and residues of ECH lead to undefined polymer compositions, these polymers would not be considered for biomedical applications. As reported by our group, high-purity GME without ECH contamination permits conventional AROP as used for pharma-grade PEG, allowing for higher molar masses and narrow molecular weight distributions of the resulting polymers (Supporting Information), Figure S50.^33^

### Polymer Synthesis

For an accurate comparison, we aimed at copolymers of the same chain length, which translates to the same degree of polymerization, systematically varying the molar fractions of GME in the backbone. The targeted chain length for all rPEGs was set to 113 monomer units, which is equivalent to a common PEG of 5000 g mol^–1^. As a reference point, mPEG of a similar chain length was synthesized under the same conditions. The degrees of polymerization and compositions for all synthesized polymers are given in Table 1. In the following sections and Table 1, the copolymer composition is described with ${rPEG}_{D_{p}}^{f}$ where ***f*** is the molar fraction of GME and ***D***_**p**_ is the degree of polymerization of the sample. For mPEG and poly­(GME) (PGME) homopolymer, only the ***D***_**p**_ is given.

**Table 1:** Characterization Data of rPEGs of Varying Monomer Compositions

polymer	mol % GME (target)	mol % GME^a^	*M*_n_ (target)	*M*_n_ ^b^	*M*_n_ ^c^	*D*_p_ (target)	*D*_p_ ^b^	*D̵* ^c^
**mPEG**_**106**_	0	0	5010	4724	4060	113	106	1.06
**rPEG**_**111**_^**0.26**^	25	26	6243	6217	4860	113	111	1.05
**rPEG**_**111**_^**0.35**^	33	35	6640	6617	5500	113	111	1.07
**rPEG**_**106**_^**0.41**^	40	41	6948	6629	4990	113	106	1.07
**rPEG**_**110**_^**0.51**^	50	51	7477	7384	5710	113	110	1.08
**rPEG**_**105**_^**0.64**^	60	64	7962	7593	5820	113	105	1.06
**rPEG**_**105**_^**0.73**^	70	73	8446	8007	5850	113	105	1.06
**rPEG**_**115**_^**0.82**^	80	82	8931	9292	7160	113	115	1.06
**PGME**_**103**_	100	100	9900	9085	6520	113	103	1.05

a Calculated by ^1^H NMR spectroscopy.

b Calculated by MALDI TOF mass spectrometry.

c Calculated by SEC.

Random incorporation of the comonomers during copolymerization is essential to obtain an ideally random monomer sequence in the backbone. In a previous study, we demonstrated that copolymerization of EO and GME in dimethyl sulfoxide (DMSO) indeed leads to a perfectly random incorporation of both comonomers.^33^ In situ ^1^H NMR kinetics in several different aprotic solvents showed reactivity ratios of ***r***_**EO**_ ≈ ***r***_**GME**_ ≈ 1 in DMSO-*d*_6_.^33^ Therefore, all polymerizations were performed at 30 °C in DMSO. Based on these reactivity ratios, the composition of the copolymers is determined by the composition of the monomer feed. The polymerization reaction and basic characterization data are depicted in Figures 2 and 3. Further details regarding polymer synthesis and full characterization data can be found in the Supporting Information.

**Figure 3:**
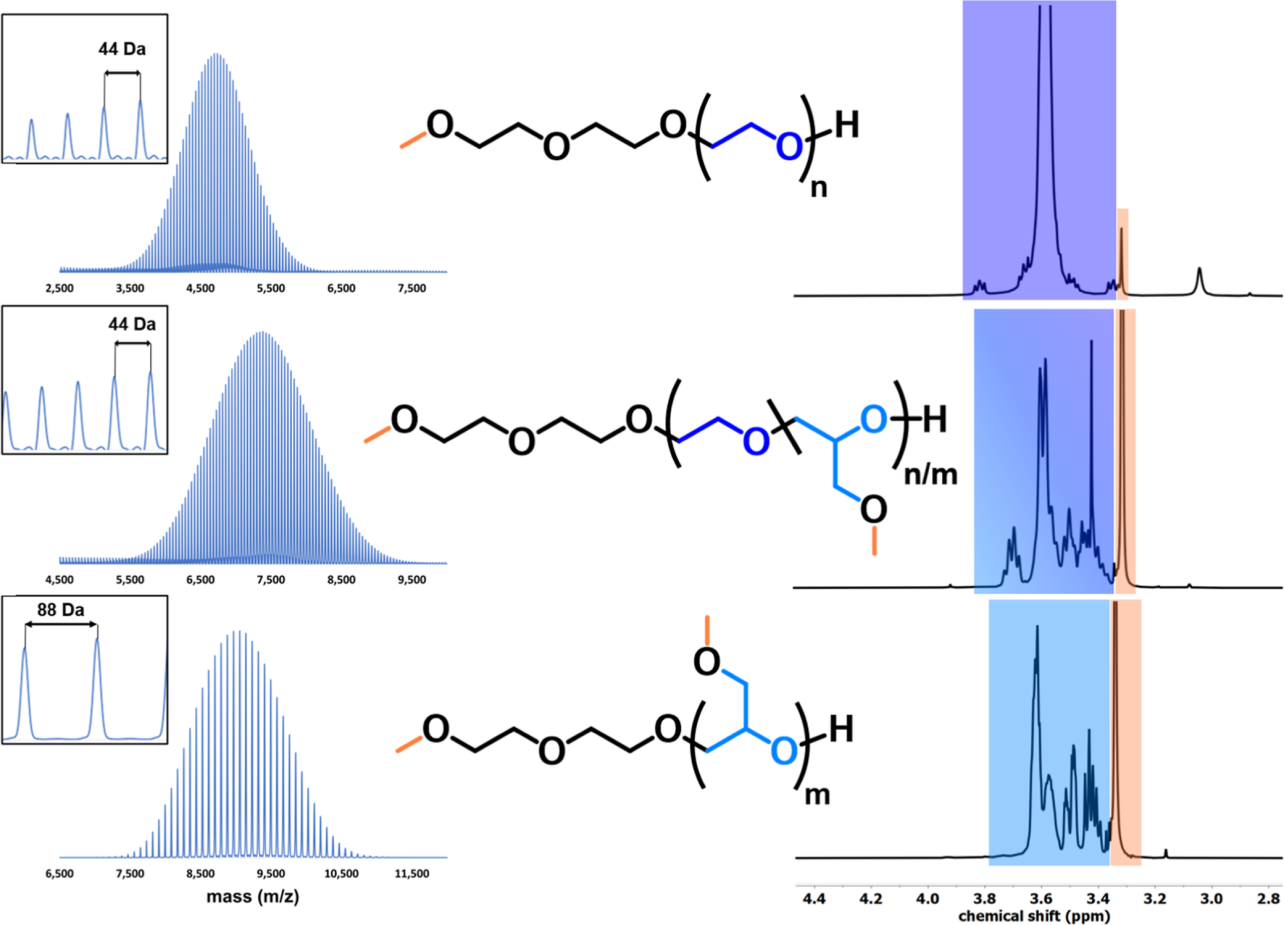
Comparison of characterization data recorded for **mPEG**_**106**_ (top), **rPEG**_**110**_^**0.5**1^ (middle), and PGM**E**_103_ (bottom). Typical MALDI TOF spectra of the polymers are shown on the left.

Most copolymers were prepared by using a single monomer addition step. For **rPEG**_**115**_^**0.82**^ and **PGME**_**103**_, two monomer additions were necessary. However, the synthesis of PGME with a degree of polymerization exceeding 100 proved challenging. Several attempts were required to obtain a PGME sample with a degree of polymerization close to the targeted 113 with narrow molecular weight distribution. A detailed discussion regarding the issues encountered is given in the Supp. Inf. **PGME**_**103**_ was found to be an outlier in the regular synthesis of PGME, which was performed with potassium *t*-butoxide as a base in DMSO at 30 °C, using two monomer additions 24 h apart—the same conditions used for the successful synthesis of **rPEG**_**115**_^**0.82**^. Other GME homopolymers of the same series synthesized under the same conditions showed a maximum of ***D***_**p**_ ≈ 85. Further improvements can potentially be made by varying the solvent composition or using a stronger base like sodium naphthalenide to deprotonate the PGME-macroinitiator.

For comprehensive characterization of the rPEG samples, a combination of ^1^H NMR, SEC, and MALDI TOF was employed. SEC shows monomodal distributions and low values for the dispersity ***D̵*** for all rPEG samples. The molar mass of the polymers shows systematically lower values than the MALDI TOF mass spectra. Since SEC measurements are based on a PEG calibration, it can be deduced that the hydrodynamic radius of the polymers does not change linearly with increasing molar ratio of GME in the backbone, while the molecular weight does. Since GME is isomeric to exactly two EO molecules, it possesses precisely twice the molar mass of EO. For this reason, the relative molar mass increase of the polymers is equal to the molar ratio of GME in the backbone. An rPEG sample, which contains 25 mol % GME, will, for example, have a 25% higher molar mass than mPEG of the same chain length, i.e., degree of polymerization. Analysis of the data from Table 1 shows an average underestimation of the molar mass of 23% when comparing SEC and MALDI TOF of the copolymers and PGME. It should be noted that these discrepancies could be caused by different calibration techniques between the analytical methods, as is evident by the molar mass difference found for **mPEG**_**106**_ between both methods.

For accurate values of the molar masses of the polymers, MALDI-TOF mass spectra were recorded for all samples. Because of the aforementioned isomeric nature of EO and GME, the mass signals of the distinct polymer chains overlap for every possible monomer ratio, and the mass signals of rPEGs therefore appear to show the same mass of the monomer unit as that of mPEG (Figure 3, top and middle). As expected, in the mass spectrum of **PGME**_**103**_, only the monomer mass of 88 Da can be observed (Figure 3, bottom). The top and middle spectra show the main distributions of the targeted polymers, with potassium as a counterion and minor distributions with sodium as a counterion. In the top spectrum, there is also a minor distribution showing water initiation, which can occur if monomer or initiator of the polymerization still contains trace amounts of water after purification. This side reaction results in polymers with 2 hydroxyl end groups, instead of a hydroxyl and a methoxy end group. Since the polymers are otherwise identical to the targeted species, the different end groups of this small impurity do not significantly influence the thermoresponsive behavior of the polymer samples. A detailed assignment of the signals for all polymers is given in the Supporting Information.

The average molar percentage of GME in every polymer sample was determined from ^1^H NMR spectra. By calculating the ratio of the methoxy signal at 3.35 ppm and the backbone signal at 3.36–3.95 ppm, the ratio of GME units and EO units in the backbone can be obtained. Since none of the signals can be used as an absolute reference, only the ratio of GME and EO can be calculated from the ^1^H NMR spectra. A detailed calculation of the EO-GME ratios of all copolymers is given in the Supporting Information.

### Turbidity Measurements and Cloud Points

rPEGs show composition-dependent thermoresponsive behavior in aqueous solution. Their cloud points can be correlated to the molar amount of GME. Incorporation of fewer hydrophilic side chains at the polymer backbone impedes the formation of the hydration shell and therefore decreases the absolute value of Δ ***H***_**mix**_ compared to PEG. Since rPEGs are structural isomers of PEG, the effect is considerably less pronounced than in the industrially relevant EO/PO copolymers.^41^ A statistical copolymer of 50 mol % EO and PO at 5100 g mol^–1^ shows a cloud point of 55 °C,^42^ whereas a statistical copolymer of 20 mol % EO and 80 mol % PO shows a cloud point of 18 °C at a concentration of 30 wt %.^43^ Poly­(ethyl glycidyl ether) (PEGE), despite only one additional methylene group in each side chain in comparison to PGME, shows cloud points below 15 °C even at a low molecular weight of 3000 g mol^–1^ (measured at 1 mg mL^–1^), resembling poly­(propylene oxide) in its solubility behavior.^44^ These observations show that incorporation of less hydrophilic side groups at an aliphatic polyether backbone can drastically reduce the aqueous solubility of the respective copolymers compared to PEG.

To assess the hydrophilicity of the rPEGs, two different analytical methods were utilized. Macroscopically, cloud points were determined by turbidimetry, observing the clouding of polymer solutions in water by monitoring the transmission of visible light. At the nanoscopic level, polymer solutions were analyzed by cw-EPR spectroscopy using TEMPO as a spin probe, enabling us to observe the onset of chain collapse with increasing temperature on a nanometer level.

For the sake of comparison with turbidimetric data from previous publications, cloud point measurements were performed at a concentration of 5 mg mL^–1^ in Millipore water. Furthermore, several selected samples were also measured at high concentrations of 100 mg mL^–1^ (10 wt %) to be comparable with those obtained by EPR. All cloud point temperatures measured by optical spectroscopy were determined at 50% transmittance. The determined cloud point temperatures and the heating curves of these measurements for both concentrations are given in Table 2 and Figure 4.

**Table 2:** Cloud Point Temperatures of the Synthesized Copolymers for Concentrations of 5 mg mL^–1^ and 100 mg mL^–1^ ^a^

polymer	*T*_cp_ (for conc. = 5 mg mL^–1^)/°C	*T*_cp_ (for conc. = 100 mg mL^–1^)/°C
**rPEG**_**106**_^**0.41**^	96.0	85.6
**rPEG**_**110**_^**0.51**^	85.8	--
**rPEG**_**105**_^**0.64**^	83.2	71.6
**rPEG**_**105**_^**0.73**^	78.6	--
**rPEG**_**115**_^**0.82**^	73.2	68.4
**PGME**_**103**_	69.9	--

a At high concentration, only three copolymers were measured.

**Figure 4:**
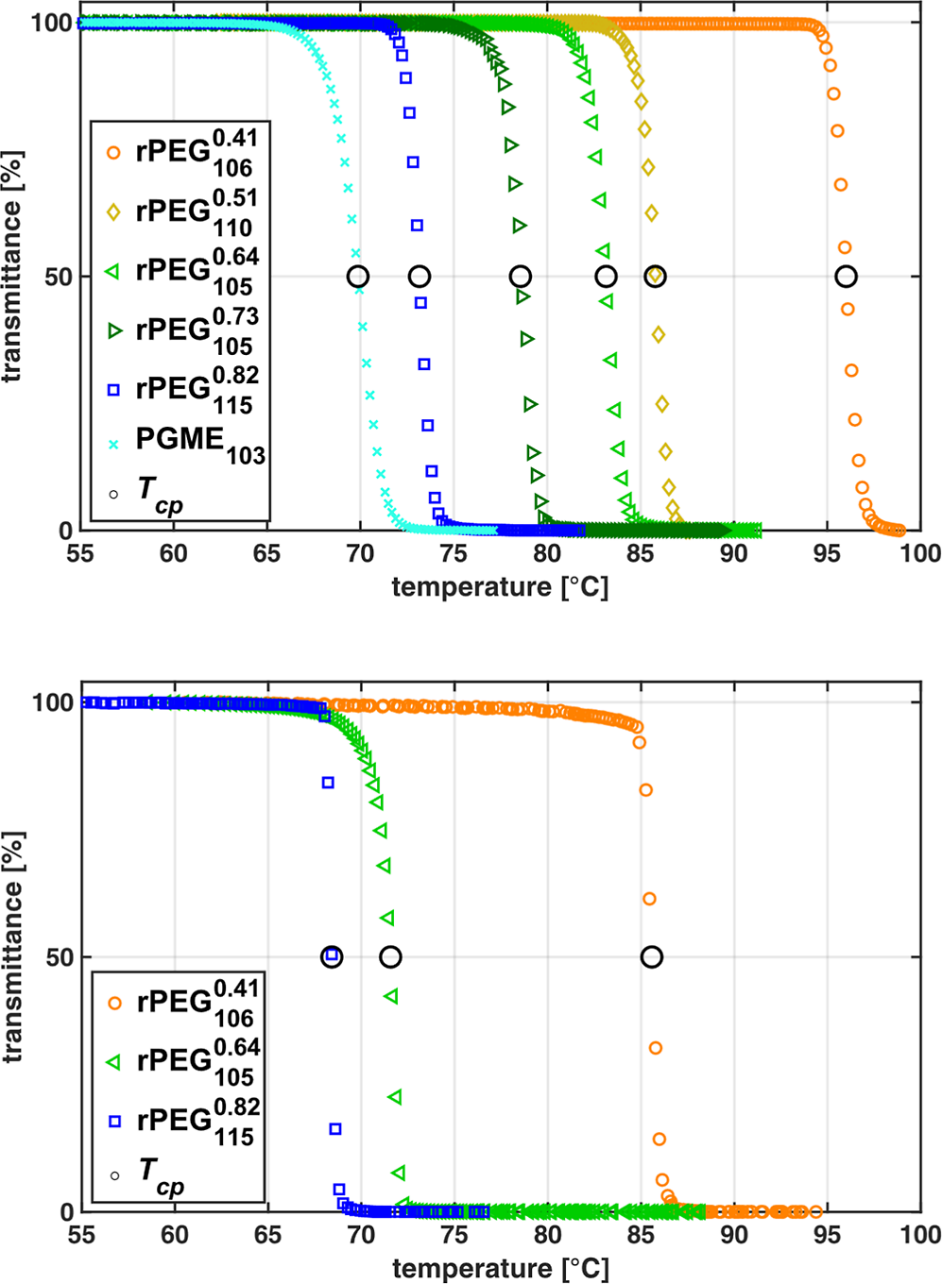
Heating curves of the cloud point measurements of rPEG samples. The respective data are summarized in Table 2. Measurements were performed at a concentration of 5 mg mL^–1^ (top) and at a concentration of 100 mg mL^–1^ (bottom) in Millipore water. Below a molar GME fraction of 41 mol %, no cloud points were observed. Data points are min–max normalized between 0 and 100% transmittance.

From the measurements, it can be deduced that an increase in the molar fraction of GME in the polymer backbone leads to a decrease in hydrophilicity. Between a GME fraction of 41 mol % and 100 mol %, a constant decrease in ***T***_**cp**_ is observed. For a GME content below 41 mol %, no ***T***_**cp**_ could be observed. Remarkably, the ***T***_**cp**_ of PGM**E**_103_ was observed at 69.9 °C, while previously published results found cloud point temperatures between 55 and 66 °C.^45,46^ The discrepancy in the values is most likely explained by the materials used, the polymerization methods by which PGME was synthesized, and the methods used for data collection.

As mentioned above, commercially available GME contains traces of ECH that are challenging to remove (see the Supporting Information). Under AROP conditions, the presence of ECH leads to chain termination reactions, broadening the molar mass distribution and limiting the achievable molar mass of the polyethers. Several publications relied on commercially available GME with a guaranteed purity of >85% for PGME synthesis,^39,45^ suggesting considerable amounts of ECH contaminant in the monomer. While all publications purify GME before use, even small amounts of ECH present in the polymers will significantly alter the properties of the copolymers such as cloud point temperatures.

In a previous work,^45^ the copolymerization of EO and GME by monomer activated ring-opening polymerization (MAROP) was reported, and a ***T***_**cp**_ of 55 °C for a PGME of 5200 g mol^–1^ was determined. Polymers in this earlier work were synthesized using tetraoctylammonium bromide and triisobutylaluminum as an initiator and catalyst, respectively. Residues of these substances could have altered solution behavior of the polymers by influencing the formation of the hydration shell, causing an earlier onset of precipitation. Furthermore, since potentially remaining ECH can be copolymerized with EO and GME by MAROP, the resulting polymer most likely shows decreased hydrophilicity because of statistically distributed ECH units that will impede the formation of the hydration shell. Besides PGME, copolymers of EO and GME were also synthesized via MAROP, exhibiting cloud point temperatures several degrees below the values found in our recent experiments^33^ and in this work. This can also be explained by the polymerization method. Since EO is incorporated preferably under MAROP conditions, gradient polymers with EO-rich and GME-rich sequences are obtained. These GME-rich sequences can potentially collapse at lower temperatures, decreasing the overall values of ***T***_**cp**_ for the copolymers.

Isono et al.^46^ observed a cloud point of 65.5 °C for a similar PGME sample of 4940 g mol^–1^ (measured at 1 wt %), which was also synthesized by MAROP. Watanabe et al.^39^ found a ***T***_**cp**_ of 57.7 °C for a PGME of 3000 g mol^–1^ in a previous work. Measurements of cloud point temperatures were also performed with 1 wt % solutions of the polymers. According to their report, ECH was removed completely before polymerization. According to our experience, distillation alone is not sufficient to effectively remove all traces of ECH, so impurities cannot be ruled out. Additionally, polymerization in this work was performed at 110 °C, which could lead to an increased amount of side reactions typical for glycidyl ethers, which in our hands were already observed at considerably lower reaction temperatures of 50 °C.

In Figure 5 (top), the measured cloud point temperatures are plotted vs the molar fraction of GME in the copolymers. Extrapolating from the measured data points listed for a concentration of 5 mg mL^–1^ in Table 3 (blue), cloud point temperatures for rPEGs with low GME content can be predicted (orange). Adding the comparable value (green) of ***T***_**cp**_ = 132.2 °C for mPEG (***M***_**w**_ = 8000 g mol^–1^, ***D̵*** = 1.6, weight fraction = 0.012) from Prausnitz et al.,^47^ a polynomial trend was found to be more accurate. Measurements were performed in a sealed sample tube in their work to keep water from evaporating. Based on this polynomial fit, cloud point temperatures for **rPEG**_**111**_^**0.35**^ (***T***_**cp**_ = 99.2 °C) and **rPEG**_**111**_^**0.26**^ (***T***_**cp**_ = 106.5 °C) could be made.

**Figure 5:**
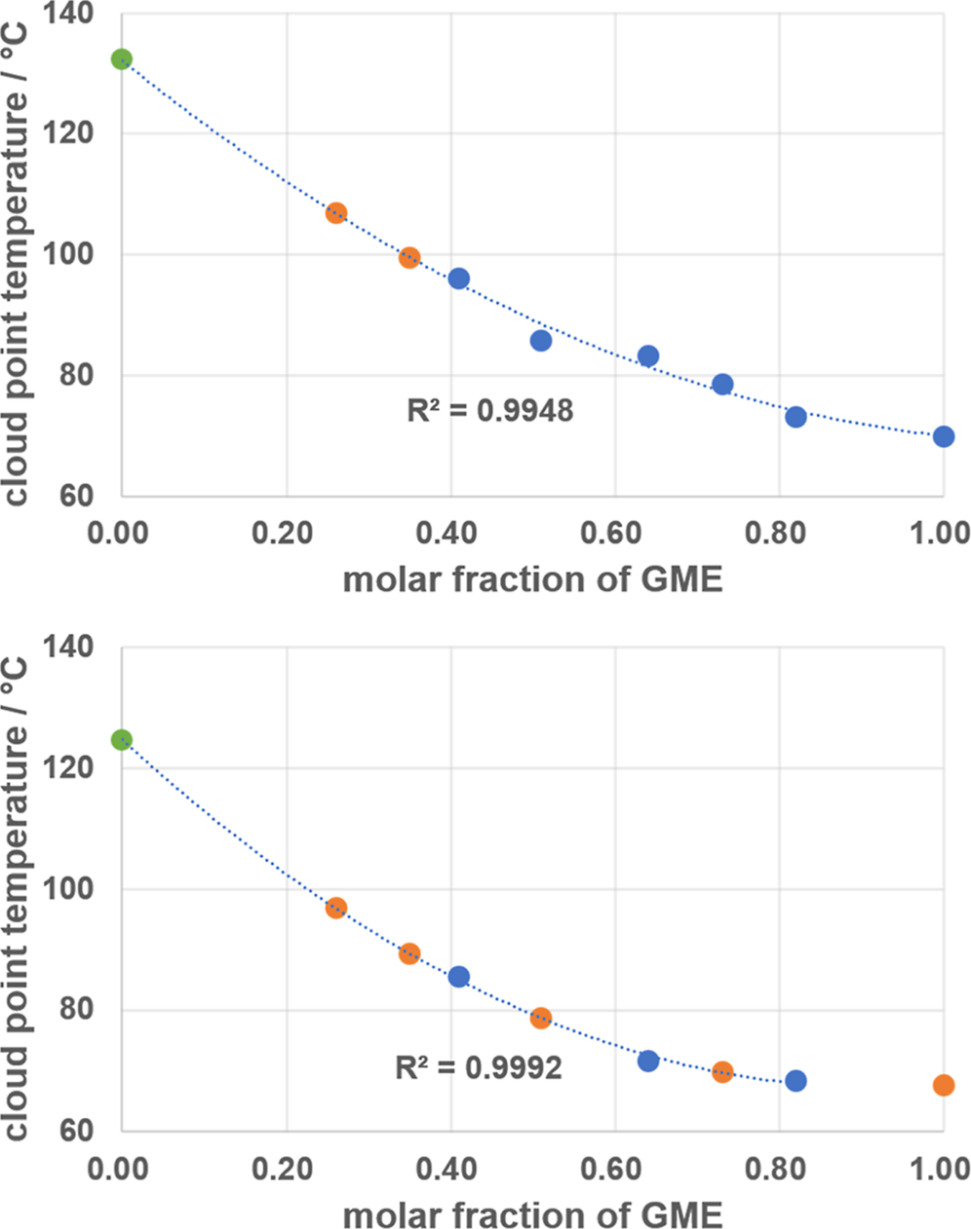
Molar fraction of GME in all rPEGs plotted against cloud point temperatures for conc. = 5 mg mL^–1^ (top) and conc. = 100 mg mL^–1^ (bottom); blue = measured values; orange = predicted values based on fit; green = literature values taken from Prausnitz et al.;^47^ polynomial fit of measured cloud points including literature value for mPEG and predicted values based on this fit.

**Table 3:** Predicted Cloud Point temperatures (Orange) Based on a Polynomial Fit of Measured Values (Blue) at Conc. = 5 mg mL^–1^ (Middle Column) and Conc. = 100 mg mL^–1^ (Right Column)^a^

polymer	***T***_**cp**_ (for conc. = 5 mg mL^–1^)/°C	***T***_**cp**_ (for conc. = 100 mg mL^–1^)/°C
**mPEG_106_**	132.2	124.6
**rPEG**_**111**_^**0.26**^	106.5	96.8
**rPEG**_**111**_^**0.35**^	99.2	89.3
**rPEG**_**106**_^**0.41**^	96.0	85.6
**rPEG**_**110**_^**0.51**^	85.8	78.7
**rPEG**_**105**_^**0.64**^	83.2	71.6
**rPEG**_**105**_^**0.73**^	78.6	69.7
**rPEG**_**115**_^**0.82**^	73.2	68.4
**PGME**_**103**_	69.9	67.7

a The cloud point temperatures of **mPEG**_**106**_ were taken from a comparable sample described in the literature^47^ (green).

### Concentration Effect on Turbidity Measurements

As can be seen in the work of Prausnitz et al.,^47^ cloud points of PEG solutions generally decrease with increasing polymer concentrations for weight fractions up to 100 mg mL^–1^ (10 wt %). This behavior is also known for other hydrophilic polymers like poly­(*N*-isopropylacrylamide) (PNIPAM)^48^ and various poly­(oxazoline)­s (POx).^49^ Therefore, similar behavior was expected for rPEG. To investigate the effect of polymer concentration on the cloud points and to gain better comparability to the EPR data, cloud point temperatures of some exemplary samples were measured at higher concentrations, as used for EPR (100 mg mL^–1^). The heating curves of these measurements are also depicted in Figure 4 (bottom). Again, a complementary ***T***_**cp**_ = 124.6 °C (***M***_**w**_ = 8000 g mol^–1^, ***D̵*** = 1.6, weight fraction = 0.100) from Prausnitz et al.^47^ was included in the fit. A polynomial fit was applied here as well. The results can be seen in Table 3 and Figure 5 (bottom). Even though a similar curve for both concentrations is obtained, further assessment will be necessary to verify the fit for high-concentration predictions since only three data points and one literature value were available for calculations. Nevertheless, similar thermoresponsive behavior of low and high polymer concentrations can be deduced from the available data.

The polynomial fit equation of cloud points ***T***_**cp**_ depending on the molar fraction of GME (***f***_**GME**_) measured at 5 mg mL^–1^ was calculated to be

1 $$T_{cp}\left(f_{GME}\right)=47.44\cdot f_{GME}^{2}-109.53{\cdot f}_{GME}+132.10$$

The polynomial equation calculated for this fit for polymer concentrations of 100 mg mL^–1^ is

2 $$T_{cp}\left(f_{GME}\right)=67.97\cdot f_{GME}^{2}-124.95{\cdot f}_{GME}+124.72$$

### EPR Observation of Cloud Points

EPR measurements on thermoresponsive polymer materials have been reported for relative polymer concentrations ranging from 1 to 15 wt %.^25,27,32,50^ This is based on the fact that the amount of polymeric nanoparticles used in pharmaceutical applications is typically higher than 10 wt %.^51,52^ Therefore, we selected a polymer content of 10 wt % to enable direct comparison of our results with previous studies on thermoresponsive polymers and still be able to model the potential applications of the newly synthesized rPEGs in therapeutic contexts such as drug delivery.

We used TEMPO at different concentrations (200 μM and 500 μM) to check for possible concentration effects in the temperature series from 10 to 90 °C used for heating and cooling cycles. We use TEMPO alone in **rPEG**_**105**_^**0.64**^ sample and of **mPEG**_**106**_. As we observed no significant change in the spectral shape and onset of transition, we used 200 μM TEMPO concentration for all other samples to avoid possible line broadening (Heisenberg spin exchange).

All measured EPR spectra and their corresponding spectral simulations are summarized in Figures S37–S46 and Tables S3–S12. The error of the spectral simulation is calculated by the RMS deviations from experimental data. For all samples, it was in a range between 10 and 15% errors, which can be considered to be in the marginal error range. The representative spectra at temperatures below, at, and above the transition temperature were chosen for spectral simulations to get a better view of variation in hydration and possible trends. In the following, we describe variation in transition temperature within three classes of polymers with low, medium, and high GME contents.

### Polymer Samples with Low Content GME (0–35 mol %: **mPEG**_**106**_, **rPEG**_**111**_^**0.26**^ and **rPEG**_**111**_^**0.35**^)

The **mPEG**_**106**_ homopolymer showed no change in polarity or spin probe dynamics during warming or cooling, regardless of TEMPO concentration (0.5–1 mM), indicating no detectable transition below or at 90 °C.^39,53^ Copolymerization of 26 mol % GME (**rPEG**_**111**_^**0.26**^) led to line broadening and reduced spectral intensity, suggesting slightly less hydrated regions; spectral simulations revealed two components with minor polarity differences (∼1 MHz in **Δ*A***_**iso**_) from 70 to 90 °C. These features, however, were not visually resolvable as splittings but were still detectable during cooling from 90 to 60 °C. No hysteresis was observed in the thermal cycles. Increasing GME to 35 mol % (**rPEG**_**111**_^**0.35**^) did not lower the cloud point; spectral simulations indicated a minor (25%) dehydrated, polymer-rich component around 80 °C, which translates to the formation of a small hydrophobic domain. Throughout cooling to 10 °C, the system remained predominantly water-rich (***A***_**iso**_ ≈ 48 MHz), with line broadening visible at around 80 °C, as shown by the spectral simulations (see Supporting Information). In summary, all samples with lower GME content remain soluble across the entire temperature range. As a representative example polymer in this series, the spectral changes in EPR spectra of **rPEG**_**111**_^**0.35**^ are shown in Figure 6. Although a decrease in the high-field line intensity is noticeable, there is no significant change in the ***A***_**iso**_ value.

**Figure 6:**
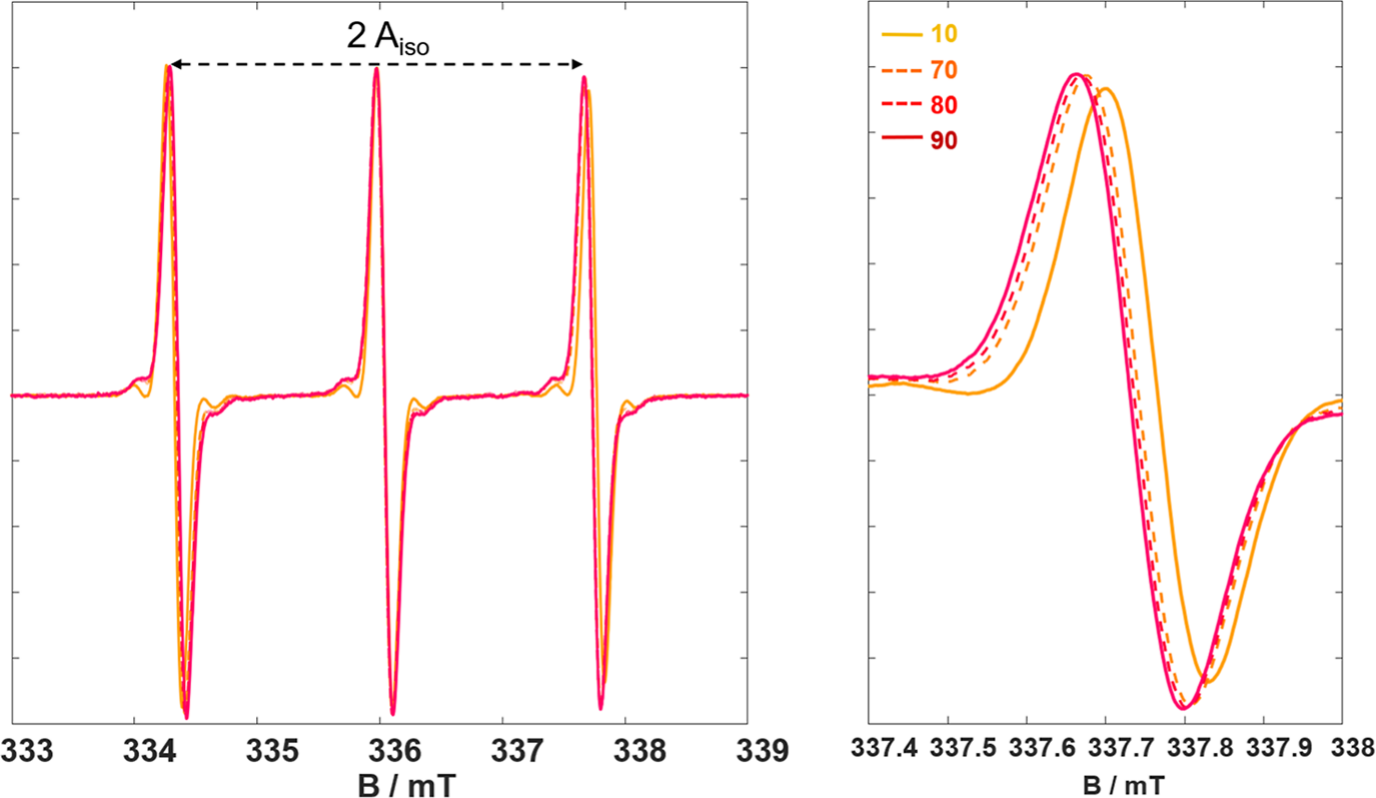
Representative EPR spectra of polymer samples with a 35 mol % GME content (**rPEG**_**111**_^**0.35**^) (left) and magnified high-field peak (right). For simplicity, only temperatures with profound changes in intensity or splitting of high filed line are shown. Temperatures before the onset of cloud points are shown in the solid line. The onset temperatures and those during transition are shown by dashed lines. The final heating temperature of 90 °C is depicted as a solid line.

### Polymer Samples with Medium GME Content (∼40–60 mol %: **rPE**G_**106**_^**0.41**^, **rPE**G_**110**_^**0.51**^, and **rPEG**_**105**_^**0.64**^)

At 41 mol % GME, no clear spectral splitting is observed. Simulations, however, indicate a two-component system at 86 °C (**Δ*A***_**iso**_ ≈ 2 MHz), marking this as the EPR transition temperature, with only 10% dehydration. Increasing the temperature to 88 °C raises the dehydrated (hydrophobic) component to about 40%, without changing spectral parameters (***A***_**iso**_ or ***g***_**iso**_). At 90 °C, the polymer loses even more bound water and forms distinct water-rich and water-poor areas, evidenced by differences in **Δ*A***_**iso**_ ≈ 1.5 MHz and slower tumbling rates, suggesting that the spin probe is located between these regions. Upon cooling, the polymer sample remains water-soluble down to 10 °C. No hysteresis is observed in the thermal cycles of this sample. At 51 mol % GME, the cloud point shifts ∼4 °C lower. EPR spectra show a gradual increase of splitting with temperature, dominated by a water-rich component (**Δ*A***_**iso**_ ≈ 3 MHz) and three times faster tumbling. By 86 °C, the polymer-rich component dominates (∼60%), and at 90 °C, both components are fully distinguishable. The samples remained water-soluble during cooling cycles. No hysteresis behavior is observed. At 64 mol % GME, increasing of local hydrophobicity showed up around 70 °C, accompanied by a decrease in ***A***_**iso**_ and intensity drop. From 86 °C onward, spectral separation increases, with a dehydrated component at 90 °C. During the cooling-down cycle, TEMPO remains in water-rich areas, indicating the polymer remains water-soluble.

To conclude, in this group of samples, solubility is lowered around 41 mol % GME, but even at 90 °C, these samples remain predominantly water-soluble (∼60% of TEMPO in water-rich regions). Increasing GME content to 51 mol % and 64 mol % resulted in a slight decrease in solubility (∼10%), above 80 °C. Alteration in the local polarity of polymer in water-rich and polymer-rich areas according to changes in EPR spectra for the polymer sample with 64 mol % is depicted in Figure 7.

**Figure 7:**
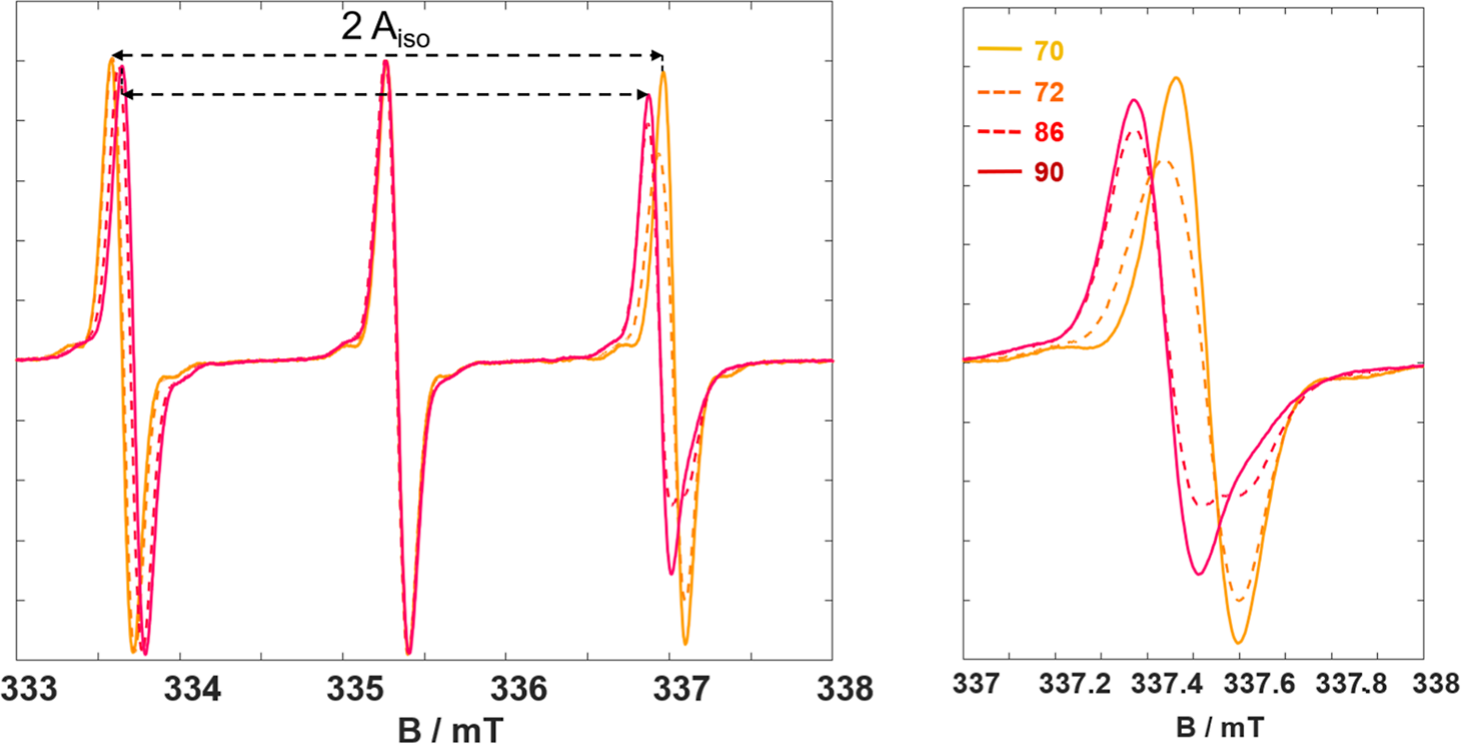
Representative EPR spectra of polymer sample **rPEG**_**105**_^**0.64**^ at selected temperatures (left) and magnified high-field peak (right). For this sample, distribution of polymer sample in water-rich and polymer-rich areas is reflected by the two different Aiso values. See Figure 6 for temperature legend descriptions.

### Polymer Samples with High GME Content (∼70–100 mol %: **rPEG**_**105**_^**0.73**^, **rPEG**_**115**_^**0.82**^, and **PGME**_**103**_)

In case of the 73 mol % GME sample, initial changes occurred around 68 °C, marked by a drop in the high-field line intensity and line broadening at 70 °C. At 74 °C, two detectable species emerged, evidenced by an ***A***_**iso**_ difference of ∼3 MHz and a change in ***g***_**iso**_ from 2.0059 to 2.0057. Also, the more hydrophobic component (43%) showed 10-fold slower rotational dynamics. These observations indicated the beginning of polymer collapse and TEMPO localization in polymer-rich areas. This partly dehydrated (collapsed) state persists up to 90 °C and during cooling to 74 °C. Subsequently, the samples remained in a water-rich, single-component system.

These observations show that solubility declines sharply at 70–78 °C, as hydrophobic interactions become dominant due to changes in the structure of aggregated (formed at transition temperature) and local polarity alterations. When GME exceeds 70 mol %, increased concentration of methyl groups promotes hydrophobic hydration^54,55^ and aggregation. During the hydrophobic hydration process, water molecules assemble into cage-like structures around a hydrophobic molecule, forming hydrogen bonds between these aqueous domains. Around the transition temperature, these structures are disrupted, and the hydrophobic nature of the solvated polymer is more pronounced. Therefore, a decrease in the water-rich component fraction and consequently an increase in hydrophobicity are observed. This is evidenced by high-field line splitting (∼3 MHz difference in ***A***_**iso**_) in EPR spectra, indicating two distinct hydration environments.

At 82 mol % GME, spectral broadening and intensity drop occurred at 68 °C with about 60–63% dehydration. From 68 to 90 °C, the spectra indicate a growing fraction of polymer-rich component (up to 60%), while collapsed regions remain partially hydrated. Cooling of the sample to 72 °C retrieved hydration partly and afterward completely.

For the homopolymer **PGME**_**103**_, spectral changes started at 60 °C, in accordance with macroscopic turbidimetry measurements reported in the literature.^39,56^ Spectral simulations showed the dominance of a water-rich component (∼92%) with faster tumbling rates, indicating full water solubility at 60 °C. Heating from 60 to 90 °C increased the collapsed (dehydrated) fraction from 8% to 68%, with a gradual decrease in ***A***_**iso**_ (∼2 MHz). At higher temperatures, the polymer-rich and hydrated components are well separated by differences in both polarity and tumbling rates. The full separation between water-rich and polymer-rich areas of the polymer sample **PGME**_**103**_ is illustrated in Figure 8.

**Figure 8:**
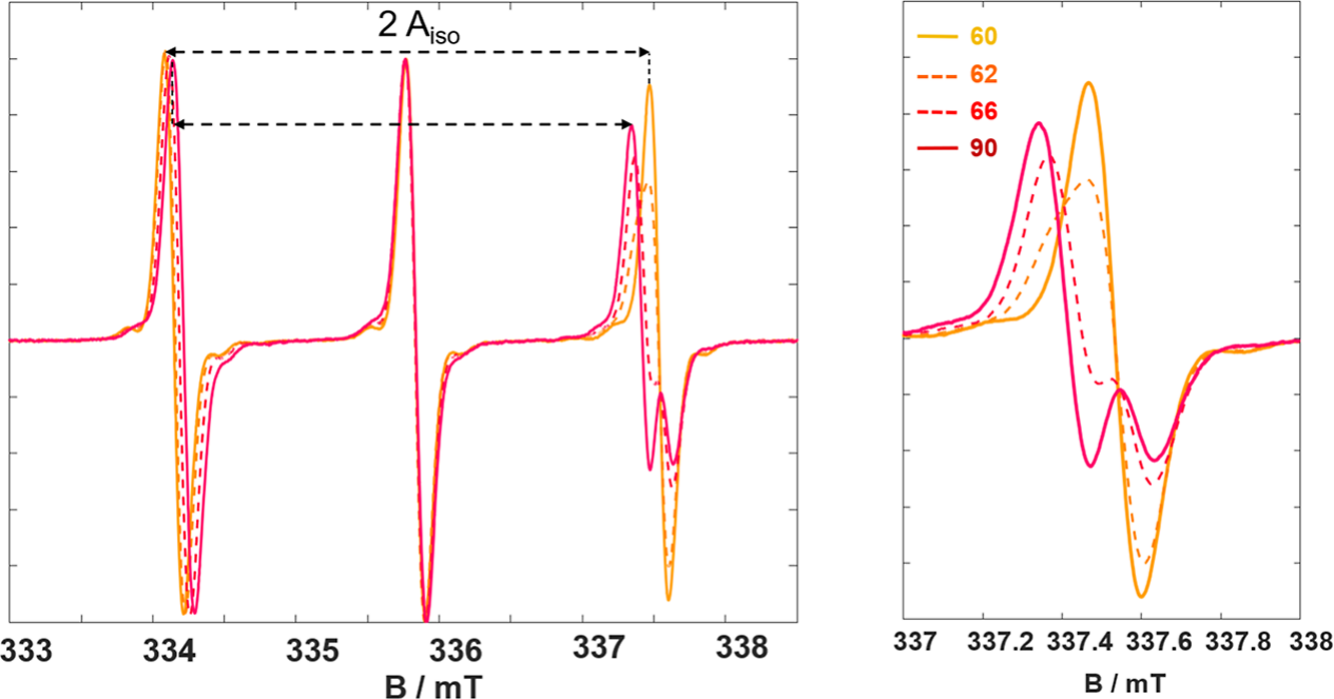
Representative EPR spectra of polymer sample **PGME**_**103**_ at selected temperatures (left) and magnified high-field peak (right). For this sample, presence of a collapsed polymer structure at high temperatures is obvious through a clearly damped intensity and splitting in the high-field line of EPR spectra.

In short, at higher GME contents (82 and 100 mol %), an unexpected increase in solubility (∼50%) is observed. This may result from steric effects of methoxy groups preventing ordered packing, which alters polymer conformation. The solubility trends reflect the balance between hydrophilic and hydrophobic segments, similar as observed in elastin-like polypeptides,^57^ where the formation of aggregate structure is directed by the length of hydrophilic block at a certain ratio of hydrophilic and hydrophobic regions. Above a certain threshold (∼30% hydrophilic content), the polymer forms hydrophobic cores with hydrated peripheries, influencing the overall aggregation and solubility. The temperature dependence of the water-rich component, derived from spectral simulations, illustrates these effects.

Spectral simulations clarify TEMPO distribution between water- and polymer-rich regions, enabling the plotting of ***A***_**iso**_ of water-rich component fractions versus temperature. These plots offer a clear representation of sample solubility behavior and transition points, as illustrated for the two samples **rPEG**_**106**_^**0.41**^ and **PGME**_**103**_, where the onset of phase transition is marked by the separation of ***A***_**iso**_ values corresponding to different polarities (see Figure 9). The temperature dependence of ***A***_**iso**_ for the rest of polymer samples is given in Figure S47.

**Figure 9:**
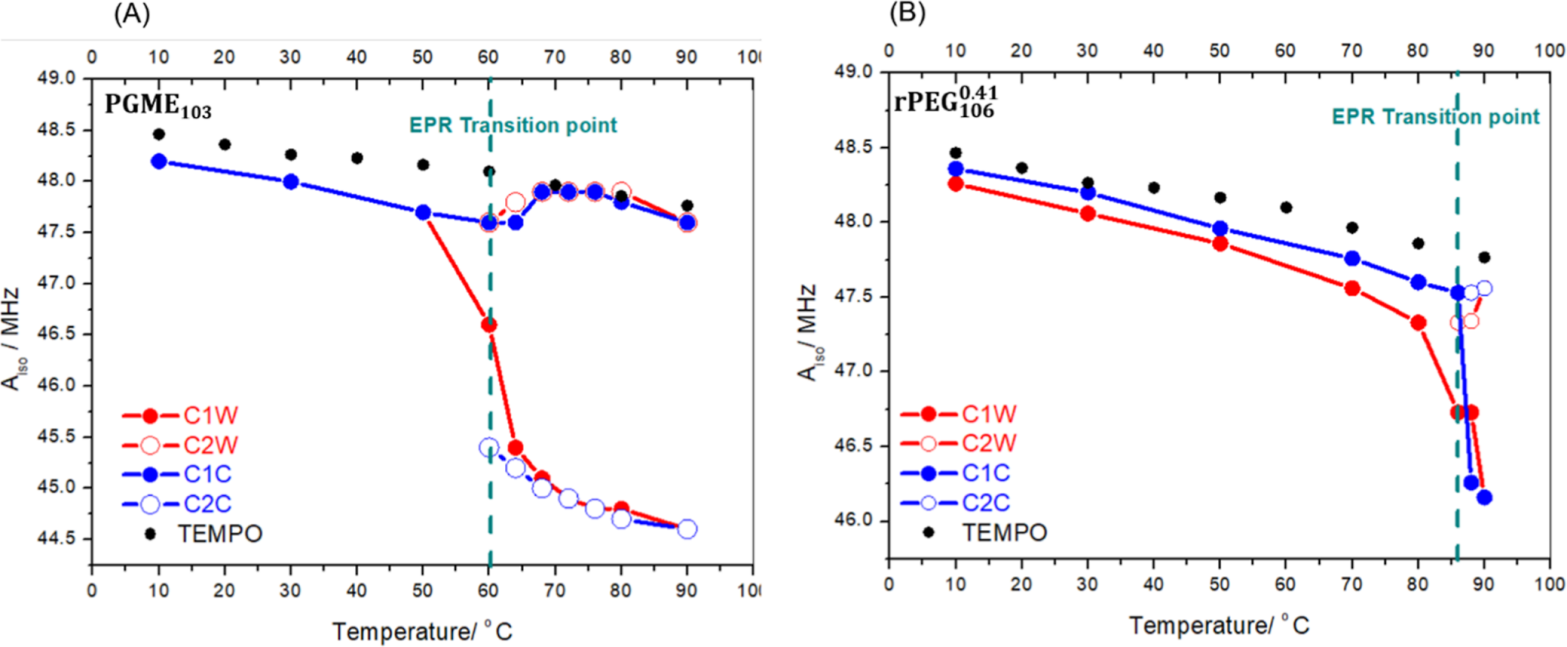
Change in local hydration (***A***_**iso**_/MHz) of polymer samples (A) **PGME**_**103**_ and (B) **rPEG**_**106**_^**0.41**^ due to temperature variation, as detected by EPR spectroscopy. The first and second components are abbreviated as C1 and C2, respectively. Simulated data for warming up (w) and cooling-down (c) cycles are shown in red and blue curves, respectively. For the sake of comparison, isotropic hyperfine coupling of the spin probe (TEMPO) is shown as black points. EPR-based transition temperatures are indicated by dashed lines.

The observed solubility trend from the EPR data is shown as the temperature dependence of the water-rich component obtained through spectral simulation (see Figure 10A). We can also represent the polymer behavior in terms of overall hydrophobicity. Since TEMPO is an amphiphilic spin probe, it can be partitioned in media of different polarities or hydrophilicity/hydrophobicity.

**Figure 10:**
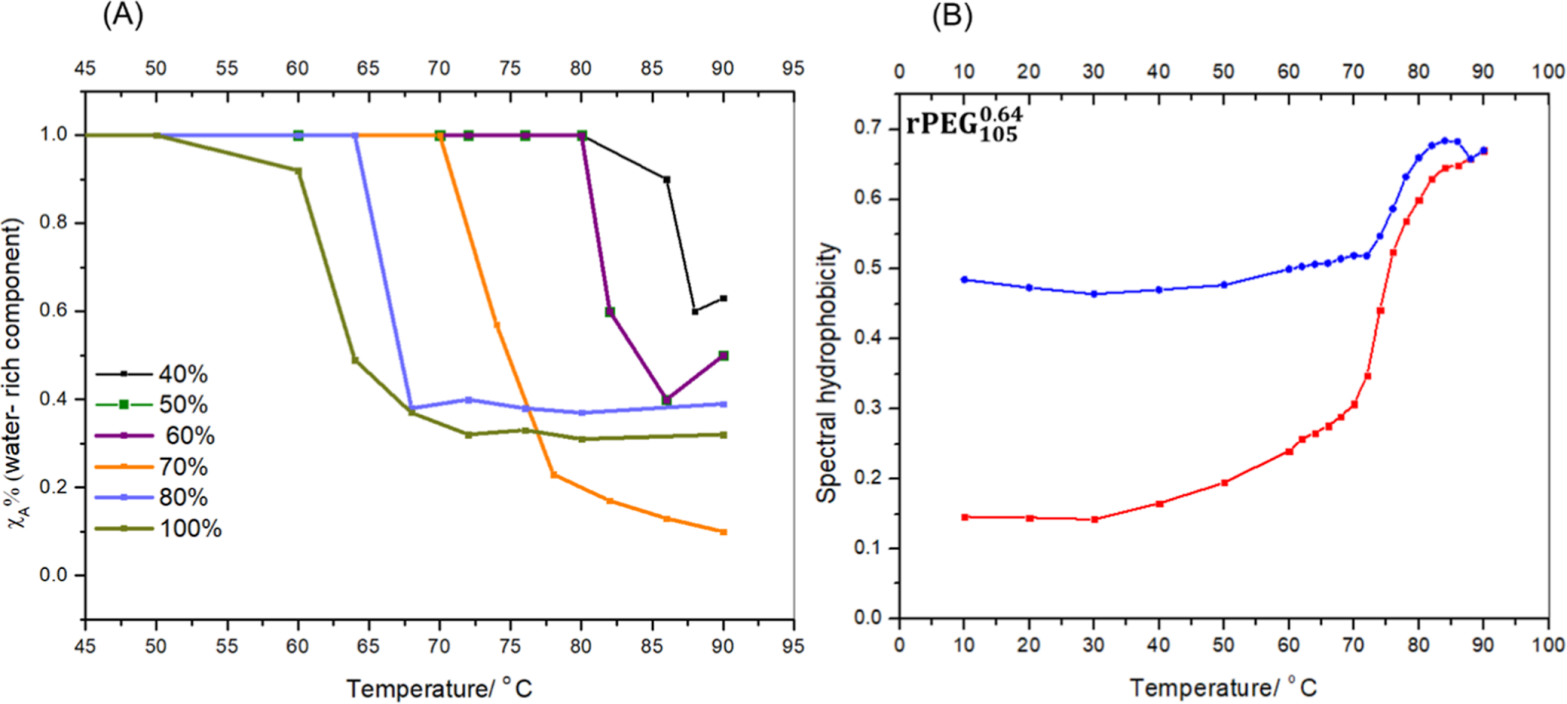
Solubility and spectral hydrophobicity of polymer samples, as detected by EPR spectroscopy. (A) Temperature dependency of the fraction of TEMPO in the aqueous phase for samples with medium to high (40–100 mol %) content GME. (B) Spectral hydrophobicity of the polymer sample with a 64% GME content that increases by the increase of temperature. Data points during heating and cooling cycles are shown in red and blue, respectively.

We derive a simple measure, the spectral hydrophobicity, to quantify the change in the polymer samples’ hydrophobicities using the high-field line intensity of TEMPO. Through spectral hydrophobicity, we detect the local contrast between water-rich and polymer-rich areas^58^

3 $$spectral\;hydrophobicity=1-\frac{I_{baseline}-I_{\min,T}}{I_{\max,90}-I_{baseline}}$$

***I***_**max,90**_ represents the intensity at the maximum of the high-field peak measured at 90 °C, and ***I***_**min,T**_ is the intensity of the minimum of the “first component” (the hydrophilic component) in the high-field peak at the measured temperature ***T***. The latter is subtracted from the baseline in the numerator, while the baseline intensity was subtracted from the denominator. This type of normalization ensures that the resulting intensity has a positive absolute value. The maximum of this absolute value corresponds to the maximum of the high-field peak at 90 °C and is therefore defined as “100%”, so that all resulting values range between 0 and 1. The intensity of the more hydrophobic peak in the equation is normalized by dividing it by ***I***_**max,90**_ since the parameter reflects hydrophobicity (see Figure S48). The smaller ***I***_**min,T**_, the higher is the hydrophobicity value, i.e., the larger the weighted fraction of the hydrophobic spectral component. If the height above or below the baseline of both extremes is the same, a hydrophobicity value of 0 is determined. If the respective minimum is greater than the maximum at 90 °C, negative spectral hydrophobicity values are obtained, which can be translated as less hydrophobic environment.

For rPEGs with a GME content of less than 35 mol %, this measure is not relevant, since these samples remain water-soluble at all measured temperature ranges. For all other measured polymer samples, the spectral hydrophobicity is given in Figure S49. For example, in the case of medium-content GME polymers (e.g., **rPEG**_**105**_^**0.64**^), there is gradual increase in spectral hydrophobicity with increasing temperature (see Figure 10B). This is indicative of the TEMPO exchange between both hydrated (aqueous) and dehydrated (collapsed, aggregated segments) nanodomains in the sample. As soon as the first hydrophobic domains form (for example, start of polymer collapse at sites with more GME monomers present in the random sequence), TEMPO diffuses to these domains as it prefers them to the aqueous medium.^22^ When increasing temperature, such dehydrated regions form larger hydrophobic patches (due to physical cross-linking) and finally TEMPO cannot, on the relevant EPR time scale of several nanoseconds, exchange anymore and resides only in the now dominant dehydrated polymer segments. We termed this “dynamic inhomogeneities” that occur during temperature variation of thermoresponsive polymers.^23^ It should be noted that the choice of comonomer and its hydrophobicity are crucial in determining whether dynamic or static inhomogeneities are ultimately present.^21,22^ It is also noteworthy that there is an apparent thermal hysteresis, such that the system does not reach the original values before the heating cycle. This is indicative of long-term changes in local hydration induced through the collapse that do not reach the initial level on the time scale of our experiments (several hours).

For the maximum GME content sample, the homopolymer **PGME**_**103**_, a high water content (reflected in negative hydrophobicity) is observed up to 60 °C, and then a sharp increase in the sample hydrophobicity up to 90 °C is observed, indicative of a clear and distinct hydration behavior upon temperature increase above a certain threshold (here 60 °C). The reverse trend is observed during the cooling cycle.

The cloud point temperatures for all polymer samples, as measured by the two techniques in our study, are illustrated in Figure 11. The plot clearly indicates that EPR spectroscopy can be used as an early detector of the transition temperature range, well before it becomes apparent by optical methods at conventional concentrations. This is due to temperature-induced changes on the nanoscopic level that take place with a local equilibrium between the hydrophilic and hydrophobic parts within the polymer solution. The volume fraction of these initial collapse regions is so small that they cannot be detected via optical methods. The numerical values of the cloud points obtained by both methods are collected in Table 4.

**Figure 11:**
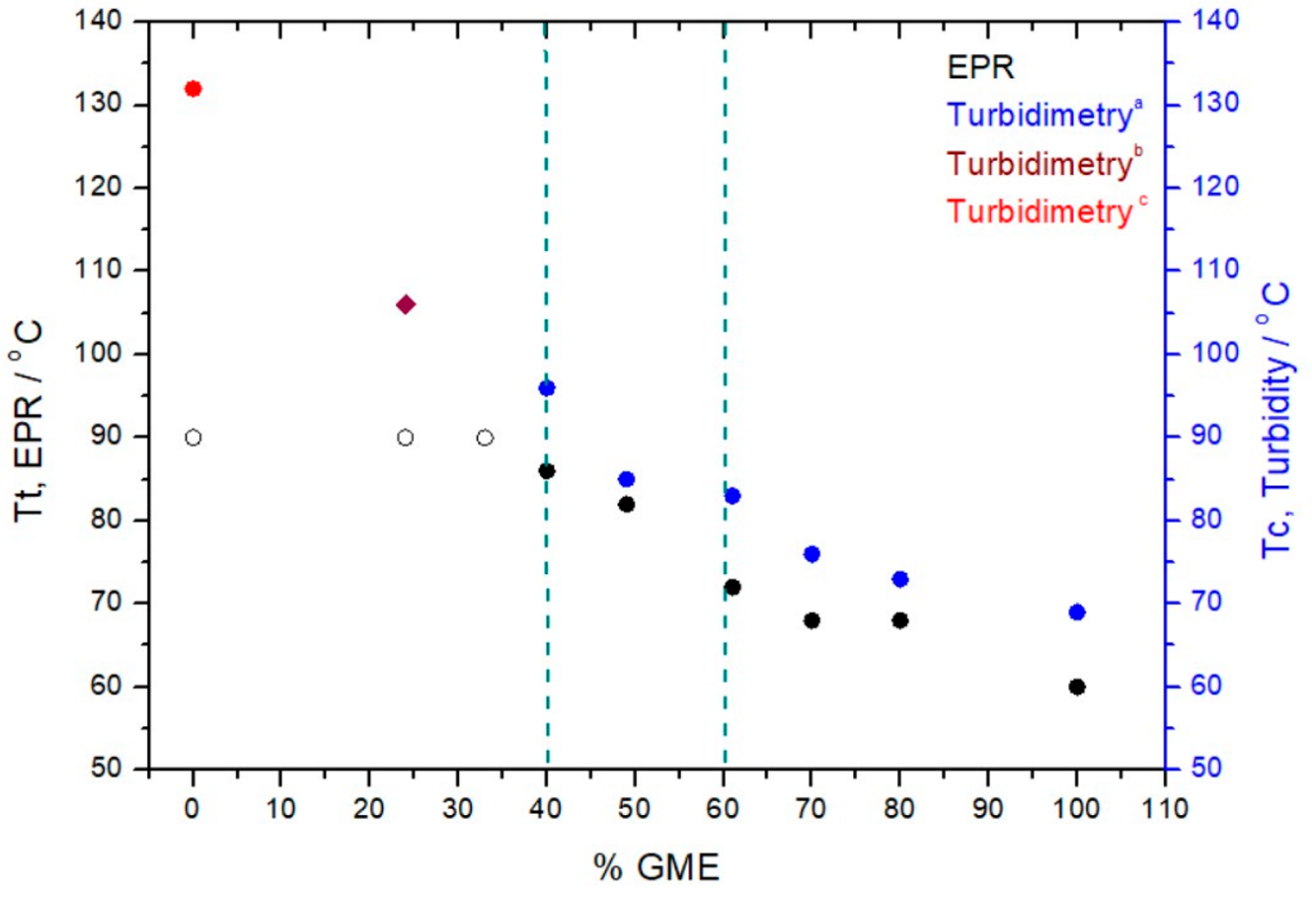
Transition temperatures in °C, obtained by EPR spectroscopy (*T*_t_) and cloud points by turbidity (*T*_c_), as a function of GME mole fraction. Turbidity data (a) from this work, (b) predicted by linear fits (c) taken from Prausnitz et al.^47^ The polymers with medium content GME (shown as the area between two dashed lines) experience a sharp drop of a transition temperature of ∼14 °C. Note that the EPR-based transition temperatures of the low-GME-content samples (0 mol %, 26 mol %, 35 mol %) can only be given as a lower limit of 90 °C and are shown as open symbols.

**Table 4:** Cloud Point Temperatures of rPEGs Measured by Optical and EPR Spectroscopies

Polymer	*T*_cp_ (turbidity)/°C (100mg mL^–1^)	*T*_on_ (onset EPR^b^)/°C (100mg mL^–1^)	*T*_cp_ (prediction^c^)/°C (100mg mL^–1^)	T_cp_ (turbidity)/°C (5mg mL^–1^)	*T*_cp_ (prediction^d^)/°C (5mg mL^–1^)
**mPEG**_**106**_	124.6^a^	>90	--	132.2^a^	--
rPEG_**111**_^**0.26**^	--	>90	96.8	--	106.5
rPEG_**111**_^**0.35**^	--	>90	89.3	--	99.2
rPEG_**106**_^**0.41**^	85.6	86	--	96.0	94.8
rPEG_**110**_^**0.51**^	--	82	78.7	85.8	--
rPEG_**105**_^**0.64**^	71.6	72	--	83.2	--
rPEG_**105**_^**0.73**^	--	68	69.7	76.6	--
rPEG_**115**_^**0.82**^	68.4	68	--	73.2	--
**PGME**_**103**_	--	60	67.7	69.9	--

a lit. value taken from Prausnitz et al.^47^

b Temperature at which the beginning of chain collapse can be observed.

c Values predicted by polynomial function (eq 2).

d Values predicted by polynomial function (eq 1).

## Conclusion

rPEGs are structural isomers of PEG, regardless of their composition based on the EO and GME. Since high hydrophilicity is imperative for application of rPEGs as alternative PEGylation agents for nanomedicine, their behavior in aqueous solution has to be assessed in detail. Therefore, cloud point behavior and nanoscopic processes involving small probe molecules that can be seen as models for, e.g., amphiphilic drug molecules, of rPEGs have been investigated by turbidity and EPR measurements. Turbidity measurements were performed at low concentration (5 mg mL^–1^) and high concentration (100 mg mL^–1^) in Millipore water, while EPR measurements were performed for high concentrations.

The data show that ***T***_**cp**_ strongly depends on the molar fraction of GME in the polymer backbone. As expected, higher contents of GME lower the hydrophilicity of the polymers also on the nanoscale and in interaction with amphiphilic small molecules compared to mPEG and therefore lead to a decrease in ***T***_**cp**_. This is due to the rather hydrophobic nature of the methyl groups of the GME. Therefore, by increasing the GME content, the phase separation between hydrophilic and hydrophobic components becomes more pronounced.

The cloud points measured by both methods are in good agreement. For molar GME contents below 41 mol %, no cloud point temperatures were observed up to 96 °C. The biggest drop in transition temperature is observed for samples with medium content GME, where an increase of ∼20 mol % of GME results in ∼14 °C lower transition temperatures. For PGM**E**_103_, transition temperatures obtained by EPR are 5–10 °C lower than the values measured by turbidimetry and polynomial prediction (even at 100 mg mL^–1^). These findings suggest a comparably large drop in hydrophilicity between 82 and 100 mol % GME contents. This observation demonstrates the importance of even small amounts of EO units in the backbone for stabilization of the hydration shell. Predicted values exhibit slight overestimation compared to values found by EPR but still provide a good approximation of cloud point temperatures for rPEGs up to 82 mol % GME.

Finally, all determined cloud points for rPEG and PGME samples were found to be in a range between 60 and 95 °C. Even at a concentration of 100 mg mL^–1^—magnitudes above concentrations used in medical applications—no ***T***_**cp**_ below 60 °C was observed. Additionally, the onset of chain collapse determined by EPR demonstrates that no partial collapse of the polymer chain, even on the nanometer scale, is to be expected below the apparent precipitation of the copolymers, which would decrease hydrophilicity and potentially trigger an immune response. Overall, high hydrophilicity of the copolymers in the temperature range below 60 °C could be confirmed for molar GME contents between 26 mol % and 82 mol % as well as for PGME, providing no indication of safety issues for rPEGs in biomedical applications with respect to their solubility in aqueous solution.

## Supplementary Material



## Notes

Materials and methods; polymer synthesis; ^1^H NMR spectra; MALDI TOF spectra; size exclusion chromatography; cloud point measurements (turbidity curves); DSC curves; EPR spectroscopy; gas chromatography; and supporting references (PDF)
