# Supplementary material for: Nanoscopic Characterization of the Thermal Phase Behavior of Random Poly(ethylene glycol-glycidyl methyl ether) Copolymers (rPEGs)
Source: Macromolecules. 2026 Jul 16;59(14):8252–65. doi: 10.1021/acs.macromol.6c01114 (PMC13421994; doi:10.1021/acs.macromol.6c01114)
Supplement: Supplementary file 1 [file ma6c01114_si_001.pdf]

Supporting Information

Nanoscopic characterization of the thermal phase behavior of random poly(ethylene glycol – glycidyl methyl ether) copolymers (rPEGs)

Dominik Schulz[1], Elena Berger-Nicoletti[1], Johannes Wingert[2], Julian Gatzki[2], Haleh Hashemi Haeri[2], Dariush Hinderberger[*,2], Holger Frey*[*,1]*

Table of contents

**1.**	**Materials and Methods**		p. 3 – 8
	1.1.	Materials	p. 5
	1.2.	^{1}H NMR (nuclear magnetic resonance)	p. 6
	1.3.	Matrix-assisted laser desorption ionization using time-of-flight (MALDI TOF) spectrometry	p. 6
	1.4.	Size exclusion chromatography (SEC)	p. 6 – 7
	1.5.	Cloud point measurements	p. 7
	1.6.	Differential scanning calorimetry (DSC)	p. 7 – 8
	1.7.	Electron paramagnetic resonance (EPR) Spectroscopy	p. 8
	1.8.	Gas chromatography	p. 9
**2.**	**Polymer synthesis**		p. 10 – 15
	2.1.	Monomer synthesis	p. 12
	2.2.	Polymerization procedure	p. 13 – 15
**3.**	**^{1}H NMR spectra**		p. 16 – 22
**4.**	**MALDI TOF mass spectra**		p. 23 – 27
**5.**	**Size Exclusion Chromatography**		p. 27
**6.**	**Cloud point measurements (turbidity curves)**		p. 28 – 33
**7.**	**DSC curves**		p. 34
**8.**	**EPR Spectroscopy**		p. 35 – 56
	8.1.	Experimental and simulated spectra	p. 35 – 46
	8.2.	Spin Hamiltonian parameters derived from simulations	p. 47 – 56
**9.**	**Gas chromatography**		p. 57
**10.**	**Supporting References**		p. 58

1. Materials and Methods

The calculation of the GME content and total degree of polymerization ($D_p = D_{p,GME} + D_{p,EO}$) in the rPEG samples is not feasible *via* end group analysis from the respective ^{1}H NMR spectra. Because of the signal overlap of the methoxy group deriving from the initiator and the methoxy methylene side groups, no reference signal can be chosen in the spectrum. Therefore, the GME content and the degree of polymerization were determined by a combination of MALDI TOF MS and ^{1}H NMR spectroscopy.

For calculation of the molar fraction of GME f_{GME}, the ratio of the methoxy signal between 3.36 – 3.10 ppm (I_{MeO}) and the backbone signal between 4.00 – 3.37 ppm ($I_{backbone}$) has to be determined.

I_{MeO} is derived by the signals of the methoxy groups of the GME units and the methoxy signal of the initiator.

$$I_{MeO} = 3 \cdot D_{p,GME} + 3 \qquad \text{(eq S4)}$$

The integral of the backbone signal is composed of the methylene and methine groups of the GME units as well as the methylene groups of the EO units.

$$I_{backbone} = 5 \cdot D_{p,GME} + 4 \cdot D_{p,EO} \qquad \text{(eq S5)}$$

$$\left(\frac{I_{backbone}}{I_{MeO}}\right) = \frac{5 \cdot D_{p,GME} + 4 \cdot D_{p,EO}}{3 \cdot D_{p,GME} + 3} \qquad \text{(eq S6)}$$

Since only the relative ratio of monomers has to be calculated, a theoretical value for $D_{p,GME}$ can be chosen arbitrarily for equation S6. To minimize errors, the targeted value for a polymer of $D_p^{target} = 113$ with the respective targeted molar fraction of GME f_{GME}^{target} is chosen.

$$D(theo)_{p,GME} = D_p^{target} \cdot f_{GME}^{target} \qquad \text{(eq S7)}$$

With the value of $\left(\frac{I_{backbone}}{I_{MeO}}\right)$ derived from the NMR spectrum, $D(theo)_{p,EO}$ can be determined by equation S6. The real molar fractions of EO and GME in the backbone can then be calculated by:

$$f_{EO} = \frac{D(theo)_{p,EO}}{D(theo)_{p,EO} + D(theo)_{p,GME}} \qquad \text{(eq S8)}$$

and

$$f_{GME} = \frac{D(theo)_{p,GME}}{D(theo)_{p,EO} + D(theo)_{p,GME}} \qquad \text{(eq S9)}$$

To obtain the real degree of polymerization D_p, the molar mass determined by MALDI TOF MS $M_{n,MALDI}$ is used.

To determine the molar mass of the monomer units, the mass of the initiator methoxy M_{MeO} group is subtracted from $M_{n,MALDI}$. The degree of polymerization is then calculated by:

$$D_p = D_{p,GME} + D_{p,EO} = \frac{M_{n,MALDI} - M_{MeO}}{M_{EO} \cdot f_{EO} + M_{GME} \cdot f_{GME}} \qquad \text{(eq S10)}$$

Since

$$M_{GME} = 2 \cdot M_{EO} \qquad \text{(eq S11)}$$

and

$$f_{GME} = 1 - f_{EO} \qquad \text{(eq S12)}$$

equation S10 can be simplified to:

$$D_p = \frac{M_{n,MALDI} - M_{MeO}}{M_{EO}} (2 - f_{EO})^{-1} \qquad \text{(eq S13)}$$

To give an example, the calculations are performed for $rPEG_{106}^{0.41}$ (**Figure S10** and **Figure S19**).

$$D(theo)_{p,GME} = 113 \cdot 0.40 \approx 45$$

$$\left(\frac{I_{backbone}}{I_{MeO}}\right) = 3.53$$

Using equation (S6) results in

$$D(theo)_{p,EO} \approx 66$$

and with equations (S8) and (S9): $f_{EO} = 0.59 \;\; ; \;\; f_{GME} = 0.41$

The real degree of polymerization is then calculated using the values of f_{EO} and $M_{n,MALDI}$ in equation S13:

$$D_p = \frac{6{,}629\,\frac{g}{mol} - 31.03\,\frac{g}{mol}}{44.05\,\frac{g}{mol}} \, (2 - 0.59)^{-1} \approx 106$$

All values obtained by these calculations are given in Table 1 of the main manuscript. The integrals of the NMR spectra of the copolymers (**Figure S7** to **Figure S14**) are adjusted for the calculated real values of f_{EO}, f_{GME} and D_p. It is important to note that the 2 EO repeating units of the initiator are included in D_p.

1.1. Materials

Ethylene oxide was purchased from *Air Liquide Deutschland*. All solvents were purchased from *Thermo Fisher Scientific*. Other substances were obtained from *TCI Chemicals*, *Sigma-Aldrich*, and *Thermo Fisher Scientific*. All compounds were used as received.

1.2. ^{1}H NMR (nuclear magnetic resonance)

^{1}H and ^{13}C NMR spectra were recorded on a *Bruker Avance III HD 400* spectrometer with 400 and 100 MHz, respectively, and referenced internally to residual proton signals of the deuterated solvent. All spectra were acquired at 23 °C. Spectra were processed and analyzed utilizing the *MestReNova 14.3.3-33362* software. Deuterated chloroform was purchased from *Deutero GmbH.*

1.3. Matrix-assisted laser desorption ionization using time-of-flight (MALDI TOF) spectrometry

MALDI TOF MS measurements were carried out at *a Bruker autoflex maX MALDI-TOF/TOF* using a Smartbeam-II solid-state laser with a wavelength of 337 nm. Spectra were recorded using the software *Bruker flexControl 3.4* and analyzed using *Bruker flexAnalysis 3.4* and *Bruker polytools 1.31*. The potassium salt of triflouroacetic acid (KTFA) and trans-2-[3-(4-tert-Butylphenyl)-2-methyl-2-propenylidene]malononitrile (DCTB) were utilized as ionization salt and matrix, respectively. For sample preparation, the polymers were dissolved in chloroform at 10 mg mL^{-1}. 20 µL of this solution was combined with 20 µL of a 10 mg mL^{-1} solution of the matrix in chloroform. 5 µL of a 0.1 M solution of the salt in methanol were added and 1 µL of the resulting mixture was spotted onto a *MTP 384 ground steel target plate*. The solvents were allowed to evaporate completely before the measurement.

1.4. Size exclusion chromatography (SEC)

Measurements were conducted using an *Agilent 1100 series* HPLC system, which included a degasser, isocratic pump (G1310A), autosampler (G1313A), column oven (G1316A), and detectors for refractive index (RI) (G1310A) and variable wavelength (VWD) (G1314A). Separations were carried out employing a four-column set-up (*MZ-Analysentechnik GmbH*) connected sequentially:

i) HEMA-40 guard column (40 Å pore size, 10 µm particle size, 50 x 8.0 mm)

ii) HEMA-40 analytical column (40 Å pore size, 10 µm particle size, 300 x 8.0 mm)

iii) HEMA-100 analytical column (100 Å pore size, 10 µm particle size, 300 x 8.0 mm)

iv) HEMA-300 analytical column (300 Å pore size, 10 µm particle size, 300 x 8.0 mm)

The eluent consisted of DMF (*Fisher Chemical*) with 1 mg mL^{-1} anhydrous LiBr (*Acros Organics*), delivered at a flow rate of 1 mL min^{-1}. Both the column oven and RI detector cell were maintained at 50 °C. Calibration was performed using well-defined poly(ethylene glycol)s from PSS (*PSS Standards Kit*) with molar mass values (M_p) ranging from 106 to 42700 g mol^{-1}. Samples were dissolved in DMF (with 1 mg mL^{-1} anhydrous LiBr) at a concentration of 1 mg mL^{-1} with the addition of 1 drop of toluene. The injection of 100 µL of the stock solutions was carried out via the autosampler, with a measurement duration of 45 min. Elution times were referenced using toluene as an internal standard. RI traces were analyzed using the *PSS WinGPC Unichrom V8.31* software.

1.5. Cloud point measurements

Turbidimetric measurements were performed with a *JASCO* UV-Vis Spectrometer (V730) at a light wavelength of 600 nm and a heating rate of 1 K min^{-1} using the software *JASCO Spectra Manager Ver.2*. Polymers were dissolved in Millipore water at various concentrations. Pure Millipore water was utilized as a reference value of 100% transmittance and was measured prior to each experiment. All measurements were performed in a quartz glass cuvette from *Hellma Analytics* with a light path of 10 mm. Cloud point temperatures (T_{cp}) were determined at a transmittance of 50%. Raw data was normalized to maximum and minimum values of the respective heating curves.

1.6. Differential scanning calorimetry (DSC)

DSC measurements were performed on a *DSC250* device from *TA Instruments* using indium and *n*-octane as calibrations standards. 3 – 5 mg of the polymer samples were weighed in *Tzero* aluminum pans and measured under nitrogen atmosphere against an empty pan as a reference point. The following steps were performed for all samples:

i) Heating from room temperature to 100 °C at a heating rate of 10 °C min^{-1}

ii) Cooling from 100 °C to – 90 °C at a cooling rate of 10 °C min^{-1}

iii) Temperature kept isothermally at – 90 °C for 10 min

iv) Heating from – 90 °C to 180 °C at a heating rate of 10 °C min^{-1}

v) Cooling from 180 °C to – 90 °C at a cooling rate of 10 °C min^{-1}

vi) Heating from – 90 °C to 180 °C at a heating rate of 10 °C min^{-1}

Melting temperatures ($\boldsymbol{T_g}$) and glass transition temperatures ($\boldsymbol{T_g}$) were calculated from the last heating cycle to ensure prior removal of solvent residues as well as thermal history of the sample.

1.7. EPR Spectroscopy

A solution of 10 wt% of each sample was prepared by dissolving the polymer in a freshly prepared 0.2 mM aqueous solution of TEMPO (2,2,6,6-Tetramethylpiperidine-1-oxyl) if not mentioned otherwise.

The effect of the heating rate on the phase transition was minimized by employing a constant heating rate of 1 K min^{-1}.[1–3] According to previous studies[4], the cloud point temperature is not significantly influenced when a consistent heating rate is applied. Temperature-dependent CW EPR spectra at X-band frequencies ($\nu \approx 9.4$ GHz) were recorded using a *MiniScope MS400* (*Magnettech/Freiberg Instruments*, now *Bruker BioSpin*) equipped with the *MS400 temperature control unit, TC H02*. EPR spectra were measured at 10 K steps, ranging from 10 °C to 90 °C. To see if there is any change between warmed up polymer with the cooled down ones, the procedure was repeated by measurements from 90 °C to 10 °C. Then they finely screened at temperature intervals of 2.0 ± 0.2 °C, around transition point temperature. Reference sample measurements on TEMPO solutions were performed accordingly. Each spectrum was acquired with a sweep width of 15 mT, a modulation amplitude of 500 mG (0.05 mT), and a microwave attenuation of 20 dB (0.3 mW). Each spectrum was the result of three accumulated scans. EPR spectral simulations were performed using home written *Matlab* scripts (version 2022b) using the *EasySpin* software package[5] (version 6.0.5) and “chili” function. This function reflects slow, non-isotropic tumbling of nitroxide radicals based on the Schneider-Freed theory.[6]

1.8. Gas chromatography

Gas chromatography was performed on an *Agilent 8890 GC* using a *HP 5MS UI column* (30 m x 0.25 mm x 0.25 µm) and a flow of 1.2 mL min^{-1} helium. The flow was split to an FID and an *Agilent 5977 GC/MSD. Injector* temperature 250 °C, MS source temperature 250 °C, transfer line temperature 230 °C, quadrupole temperature 150 °C. Oven temperature program: Hold 40 °C for 2 min, heat at 16 °C min^{-1} to 200°C and hold for 5 min.

2. Polymer synthesis

We recently utilized an alternative method for the synthesis of GME, which does not rely on a base-catalyzed one step substitution but instead a reaction of epichlorohydrin under acid-catalyzed hydrolysis of the epoxide ring followed by ring closure via an intramolecular nucleophilic substitution to reform the epoxide ring. The reaction of 1-chloro-3-methoxypropan-2-ol to GME was originally published by Nakatsuji *et al.*[7] The mechanism is given in **Scheme S1**.

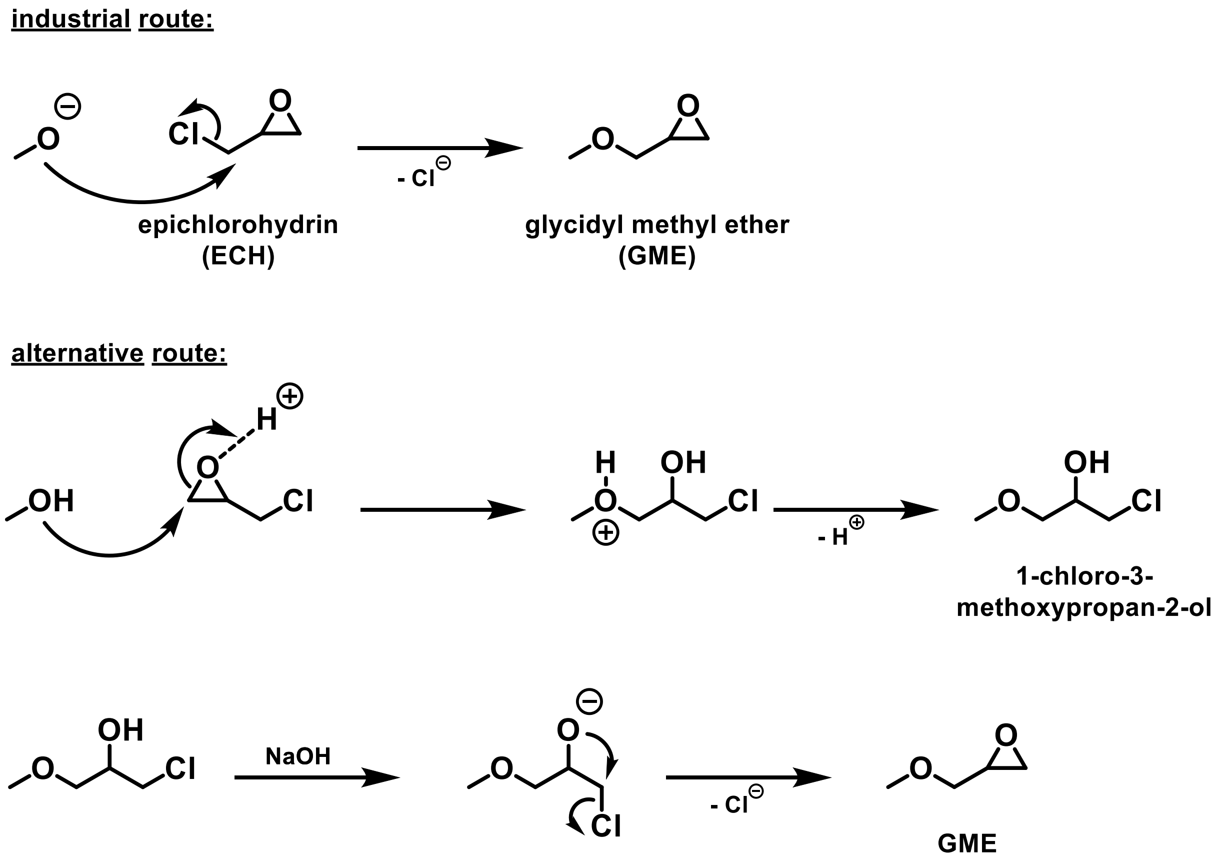

Scheme S1. Mechanism of commercial production of GME and the alternative route via intramolecular nucleophilic substitution.

The 2-step synthesis ensures complete hydrolysis of epichlorohydrin. Additionally, purification by distillation of 1-chloro-3-methoxypropan-2-ol is much more efficient, since the boiling points of the product (170 – 172 °C at normal pressure[8]) and ECH (115 °C at normal pressure[9]) are considerably more distinct than ECH and GME (105 – 107 °C at normal pressure[7]). According to our characterization, fractional distillation over CaH_2 and purification over *t*-BuOK as described in the literature[10] were insufficient to remove all residuals of ECH. A comparison of the NMR spectra excerpted from our previous work can be seen in **Figure S1**.

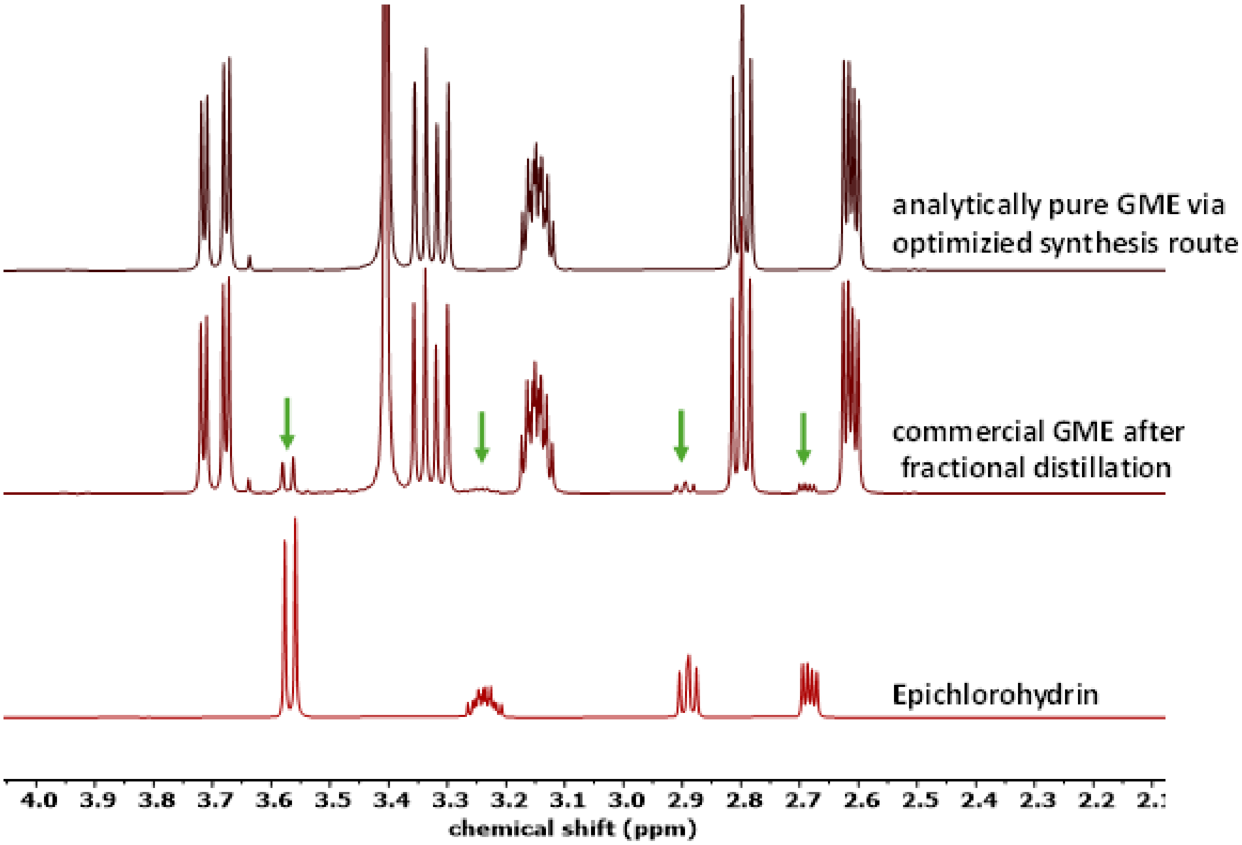

Figure S1. Comparison of ^{1}H-NMR spectra of GME synthesized by the optimized 2-step synthesis route (top), commercially available GME (middle) and epichlorohydrin (bottom). Residuals of ECH in commercial GME are highlighted (green arrows). Taken from our previous work (Published by American Chemical Society, licensed under **CC-BY 4.0**).[11]

2.1. Monomer synthesis

1-Chloro-3-methoxypropan-2-ol In a flame-dried 1000 mL three neck round bottom flask equipped with a magnetic stirring bar, a dropping funnel and a Dimroth condenser, 233 mL (183.9 g, 5.74 mol, 3.00 eq) of methanol were combined with 4 mL (7.5 g, 0.08 mol, 0.04 eq) conc. sulfuric acid (98 %). Under rigorous stirring and argon atmosphere 150 mL (177.0 g, 1.91 mol, 1.00 eq) of epichlorohydrin were added dropwise to the mixture. After the addition was finished the mixture was stirred under reflux overnight. After cooling to room temperature, 22.65 g (0.11 mol, 0.06 eq) of barium carbonate were added and the suspension was stirred for 1 h. Finally, methanol is removed at normal pressure before the product is purified at reduced pressure (Bp.: 100 – 102 °C, 60 mbar) in a fractional distillation. 191 g (80 %) of product were received as a clear, colorless liquid.

^{1}H NMR ($CDCl_3$, 400 MHz) δ: 3.99 – 3.95 (dd; J = 5.32 Hz, 5.26 Hz; 1H), 3.65 – 3.55 (qd; J = 11.13 Hz, 5.56 Hz; 2H), 3.50 – 3.48 (d; J = 5.01 Hz; 2H), 3.39 (s; 3H), 2.65 – 2.64 (d; J = 5.50 Hz; 1H).

Glycidyl methyl ether In a 500 mL three neck round bottom flask equipped with an electric overhead stirrer, a Dimroth condenser and a thermometer, 172 mL (200.0 g, 1.61 mol, 1.00 eq) of 1-chloro-3-methoxypropan-2-ol were combined with 400 mL of diethyl ether. The mixture was cooled to 0 °C in an ice bath and 77.07 g (1.93 mol, 1.20 eq) of sodium hydroxide were added in small portions (over a timespan of appr. 2 h) while the temperature was kept under 15 °C. Under constant stirring, the mixture was allowed to reach room temperature after 5 h. The residue was filtered off and flask and filter were washed 4 times with diethyl ether. The combined organic phases were dried over magnesium sulfate and diethyl ether was removed by distillation at normal pressure. Afterwards, the product was purified by fractional distillation at reduced pressure (Bp.: 70 – 72 °C, 150 mbar) to receive 95.6 g (68 %) of a clear, colorless liquid.

^{1}H NMR ($CDCl_3$, 400 MHz) δ: 3.70 – 3.66 (dd; J = 11.37 Hz, 5.26 Hz; 1H), 3.39 (s; 3H), 3.34 – 3.28 (dd; J = 11.32 Hz, 5.82 Hz; 1H), 3.15 – 3.10 (m; 2H), 2.80 – 2.77 (dd; J = 4.62 Hz, 4.57 Hz; 1H), 2.61 – 2.58 (dd; J = 5.00 Hz, 2.71 Hz; 1H).

^{13}C NMR ($CDCl_3$, 101 MHz) δ [ppm]: 73.09, 59.10, 50.63, 44.05.

2.2. Polymerization procedure

The ideally random copolymer composition of rPEGs is generally described with $rPEG_{D_p}^{f_{GME}}$, where f_{GME} is the relative molar content of GME in the polymer backbone and D_p is the total degree of polymerization. For mPEG and poly (glycidyl methyl ether) (PGME), only the D_p is given. All samples carry a methoxy and hydroxy group at the α- and ω-group, respectively.

Polymer synthesis was performed with identical procedures for most copolymers, varying only the ratio of monomers in the reaction mixture. It is exemplary described for the synthesis of $rPEG_{110}^{0.51}$ in the following paragraph.

GME was freshly dried before every polymerization by stirring over calcium hydride overnight in a flame-dried flask and subsequently cryo-transferred immediately before use.

Diethylene glycol monomethyl ether (38.7 mg, 322 μmol, 1.0 eq) was dissolved in benzene (5 mL) and transferred via syringe into a flame-dried flask equipped with a stirrer bar, stop cock, and septum. Potassium *tert*-butoxide (32.5 mg, 290 μmol, 0.9 eq) was dissolved in freshly distilled THF (3 mL) and added to the solution in the flask via syringe. The mixture was stirred for 20 min before the solvents were slowly removed under vacuum. The resulting initiator salt was dried at 60 °C under high vacuum overnight. The flask was sealed and the initiator salt was dissolved in dry DMSO (6 mL). The flask was subsequently cooled to – 90 °C with an ethanol/nitrogen cooling bath. GME (1589 mL, 18.0 mmol, 56 eq) was added via syringe into the cooled flask. Ethylene oxide (750 μL, 17.7 mmol, 55 eq) was condensed into a graduated ampule at – 90 °C and then transferred to the flask. After sealing the flask, the cooling bath was removed and the mixture was allowed to reach room temperature. After stirring for 48 h at 30 °C the polymerization was terminated by flushing the flask with argon and adding a mixture of 1M HCl and methanol (1:1, 1mL). The reaction mixture was taken up in chloroform (20 mL) and extracted twice with deionized water and subsequently with brine. The organic phase was dried with magnesium sulfate, filtered, and the solvent was removed under reduced pressure. The polymer was dialyzed against methanol (regenerated cellulose, MWCO = 2000 g mol^{-1}) for 24 h, affording a pale-yellow viscous liquid (2,073 mg, 87%).

For $mPEG_{106}$ and $PGME_{103}$, only 1 type of monomer was used. Additionally, $rPEG_{115}^{0.82}$ and $PGME_{103}$ were synthesized using 2 consecutive monomer additions of 50 % of the required monomers each. The second addition in these cases was made by cooling the reaction mixture 24 h after initiation again to – 90 °C and adding the second portion of the monomer(s). Afterwards, the mixture was thawed at room temperature and polymerization was continued at 30 °C.

Synthesis of PGME homopolymers proved challenging. Many polymerizations only resulted in PGME with a degree of polymerization of $\boldsymbol{D_p} \approx 80 - 85$, even after prolonged reaction times of several days. The reaction time was expanded from 48 h – the usual reaction time for the synthesis of rPEGs – to more than one week. Still, no increase in chain length was observed and the degree of polymerization remained at about 80 – 85. Possibly the solubility of the propagating species of PGME in DMSO decreases and the reaction solution separates into a polymer-rich and a monomer-rich phase so that chain growth is interrupted. Further attempts at increasing the monomer consumption by altering the degree of deprotonation between 90 – 98 % and increasing the reaction temperature from room temperature to up to 50 °C also proved unsuccessful. At the same time, the transfer-to-monomer side reaction (**Scheme S2**) was observed to increase when the reaction temperature was elevated. Deprotonation was then performed with cesium hydroxide instead of potassium *t*-butoxide, but only a $\boldsymbol{D_p}$ of 70 was obtained and increased transfer-to-monomer was observed as well. Similar results were found when potassium *t*-butoxide was used in combination with two equivalents of 18-crown-6 to bind the potassium ions and increase the reactivity of the active chain end. In this case, a mixture of DMSO and toluene (3:1) was employed as solvent for the polymerization to increase the solubility of the crown ether.

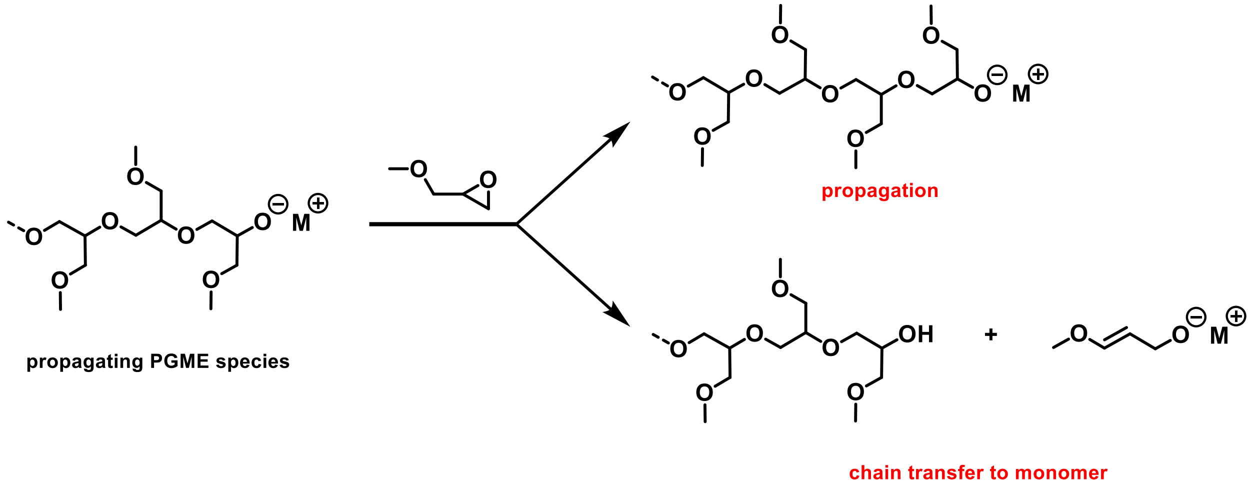

Scheme S2. Chain propagation of the active PGME species vs transfer-to-monomer side reaction.

Another attempt was made by using a previously synthesized **PGME$_{55}$** as a macroinitiator to add more monomer to the chain, but no polymerization was achieved – presumably because potassium *t*-butoxide was not able to deprotonate the macroinitiator.

3. ^{1}H NMR spectra

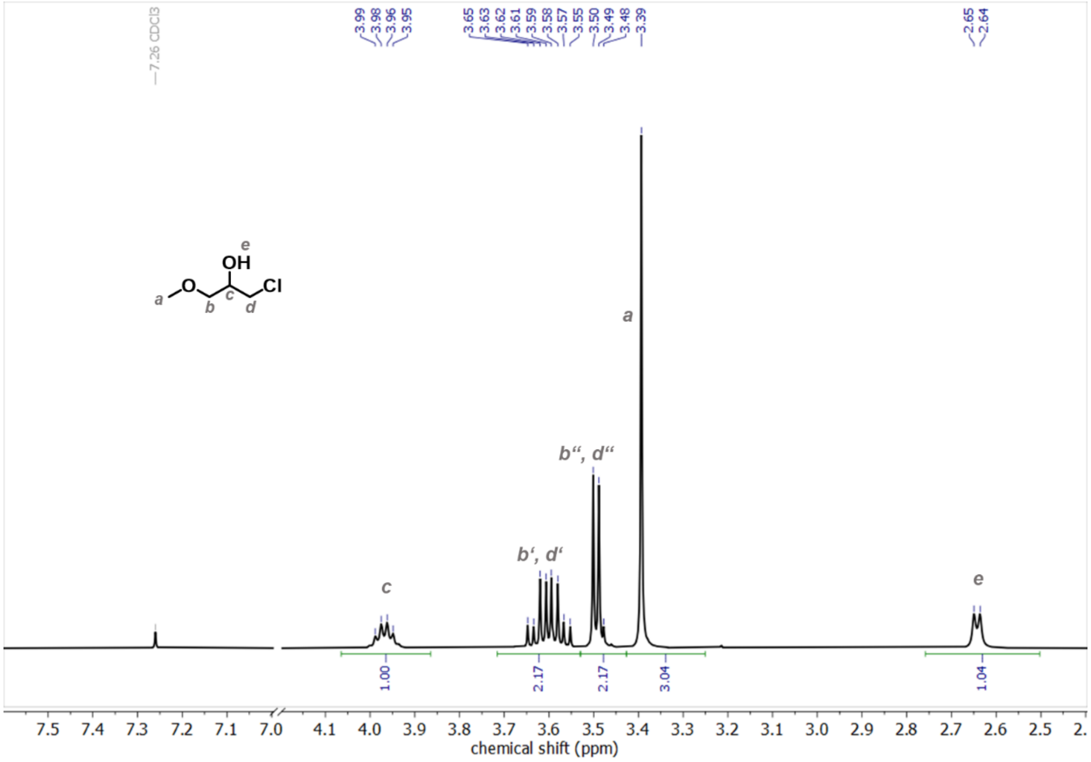

Figure S2. ^{1}H NMR spectrum ($CDCl_3$, 400 MHz) of 1-chloro-3-methoxypropan-2-ol.

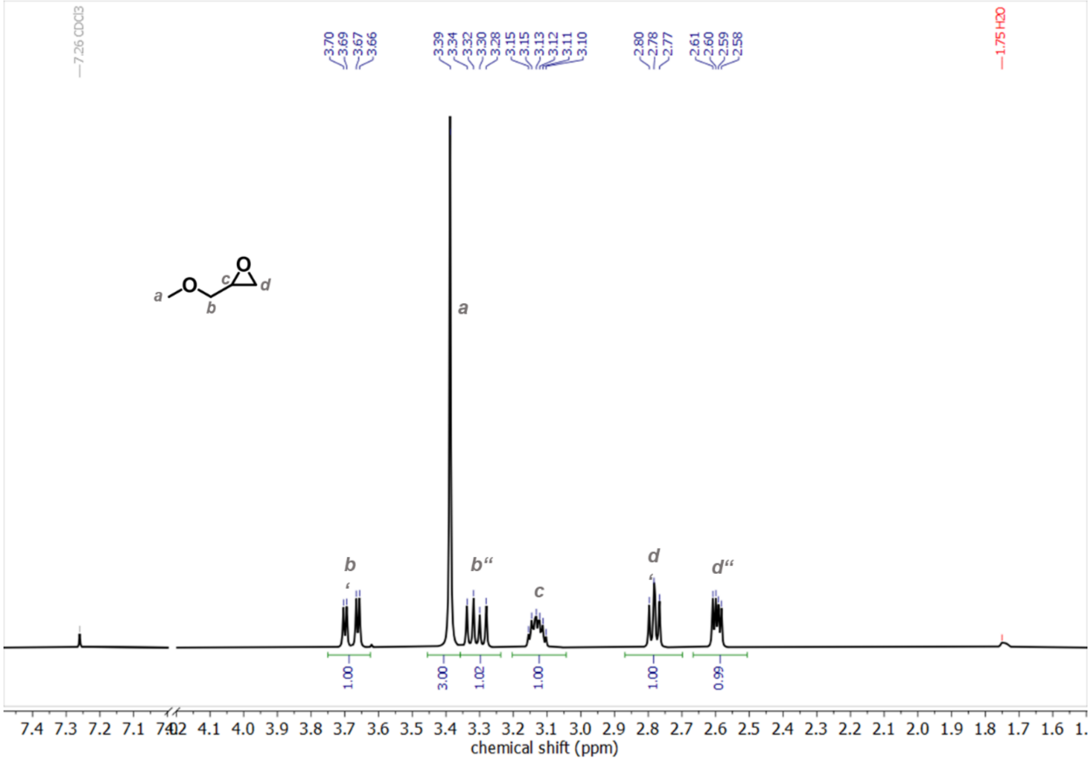

Figure S3. ^{1}H NMR spectrum ($CDCl_3$, 400 MHz) of glycidyl methyl ether.

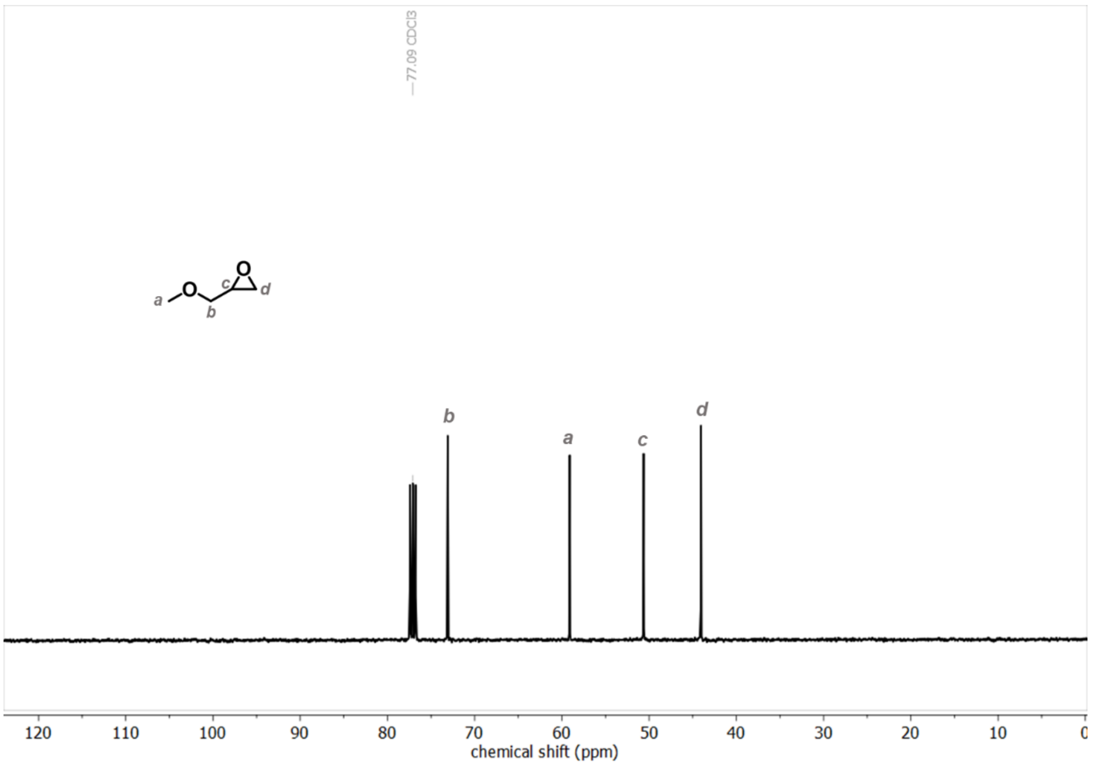

Figure S4. ^{13}H NMR spectrum ($CDCl_3$, 101 MHz) of glycidyl methyl ether.

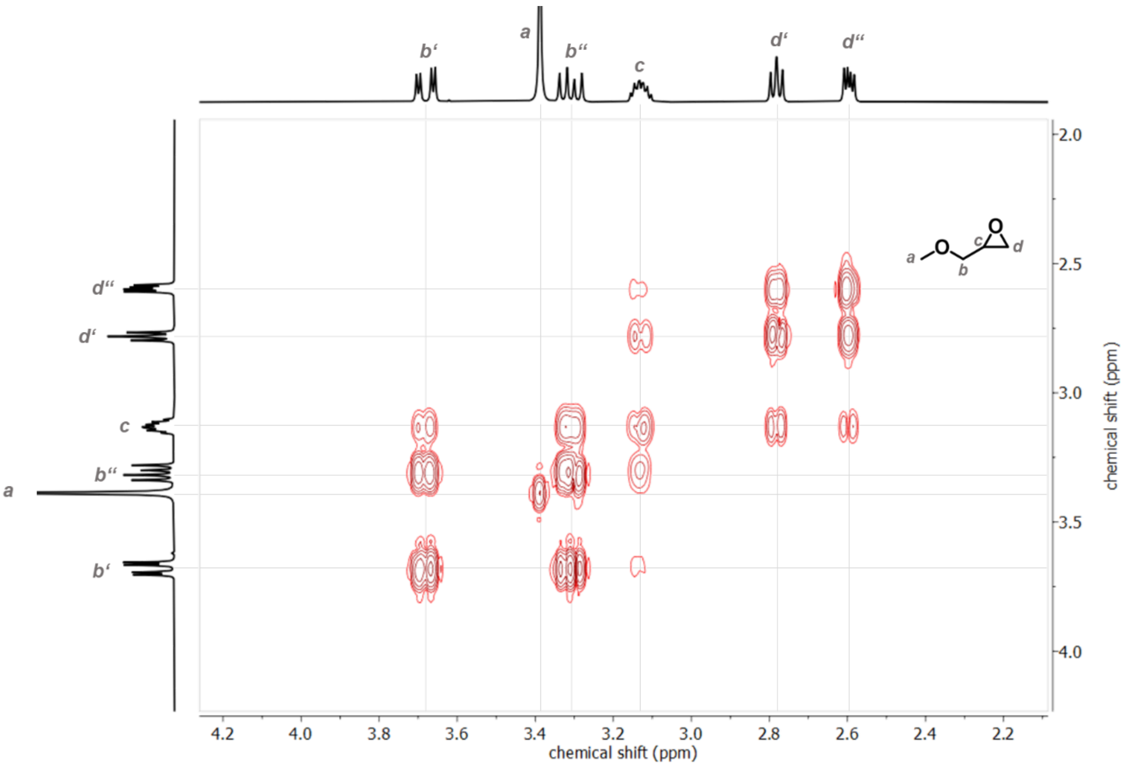

Figure S5. 2D [^{1}H, ^{1}H] COSY NMR spectrum of glycidyl methyl ether.

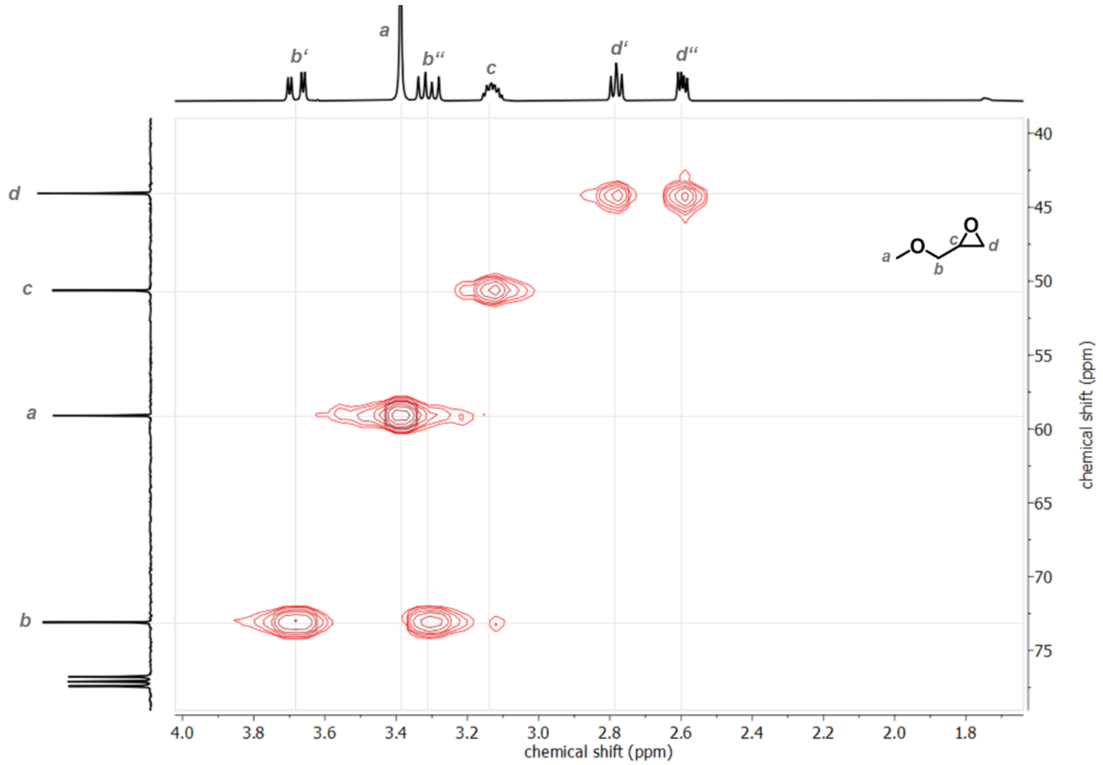

Figure S6. 2D [^{1}H, ^{13}C] HSQC NMR spectrum of glycidyl methyl ether.

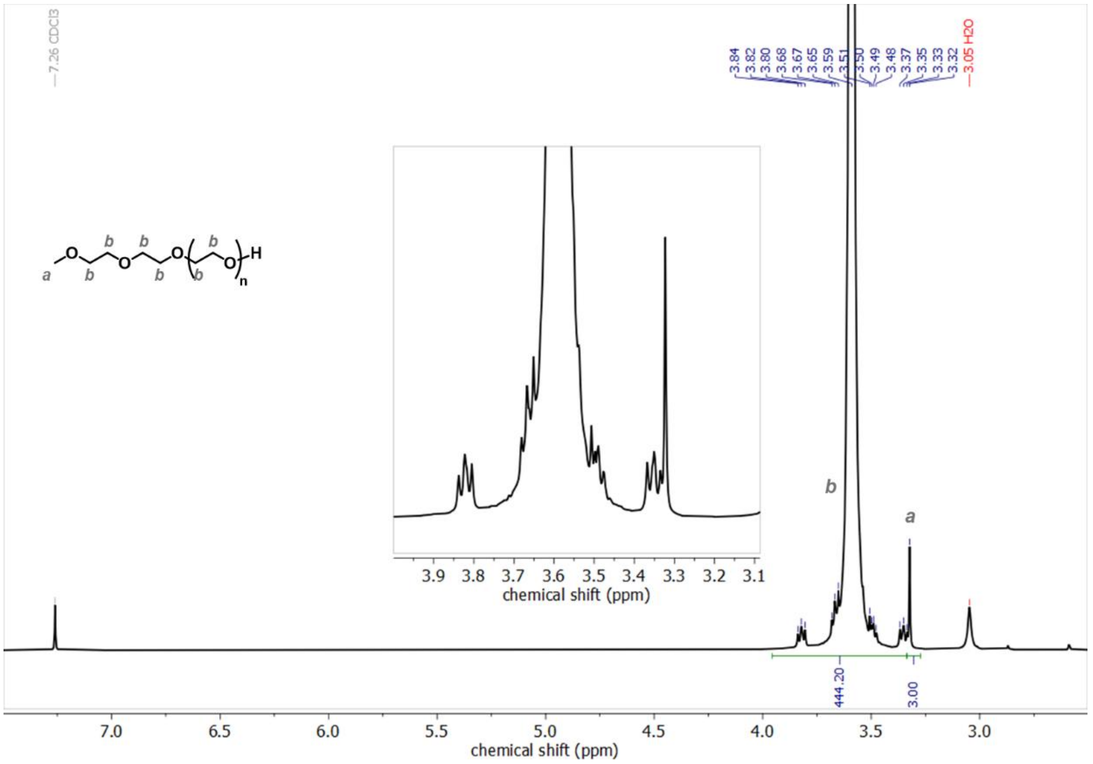

Figure S7. ^{1}H NMR spectrum ($CDCl_3$, 400 MHz) of $mPEG_{106}$.

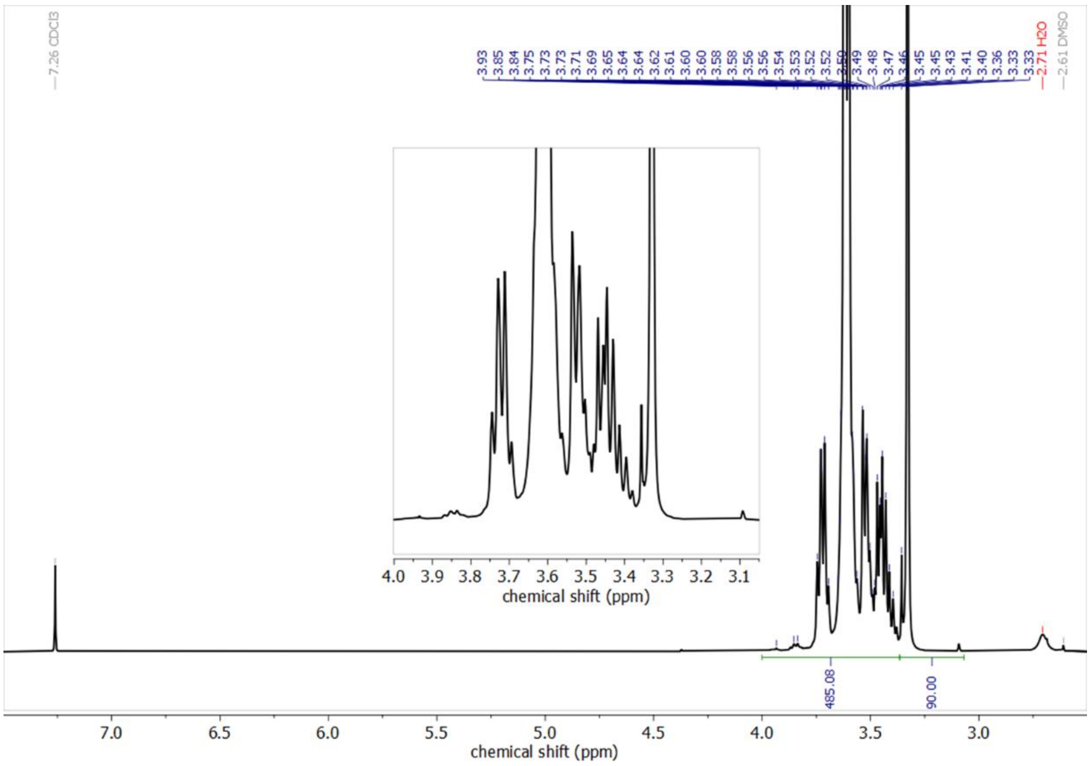

Figure S8. ^{1}H NMR spectrum ($CDCl_3$, 400 MHz) of $rPEG^{0.26}_{111}$.

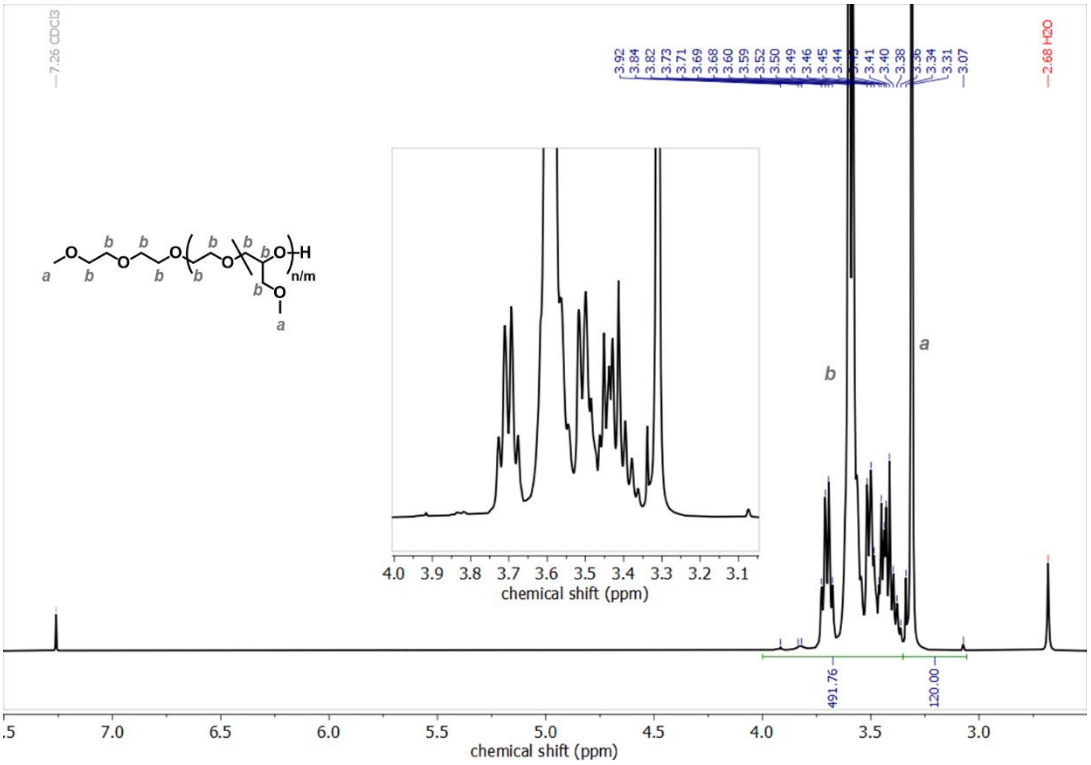

Figure S9. ^{1}H NMR spectrum ($CDCl_3$, 400 MHz) of $rPEG^{0.35}_{111}$.

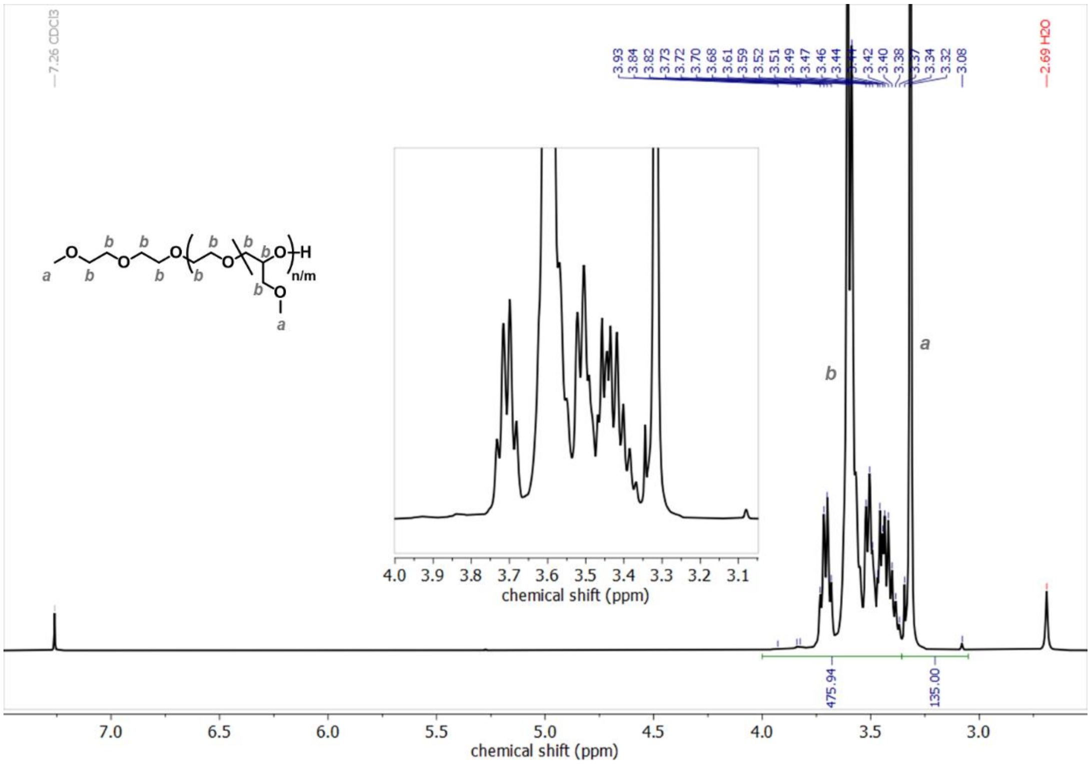

Figure S10. ^{1}H NMR spectrum ($CDCl_3$, 400 MHz) of $rPEG^{0.41}_{106}$.

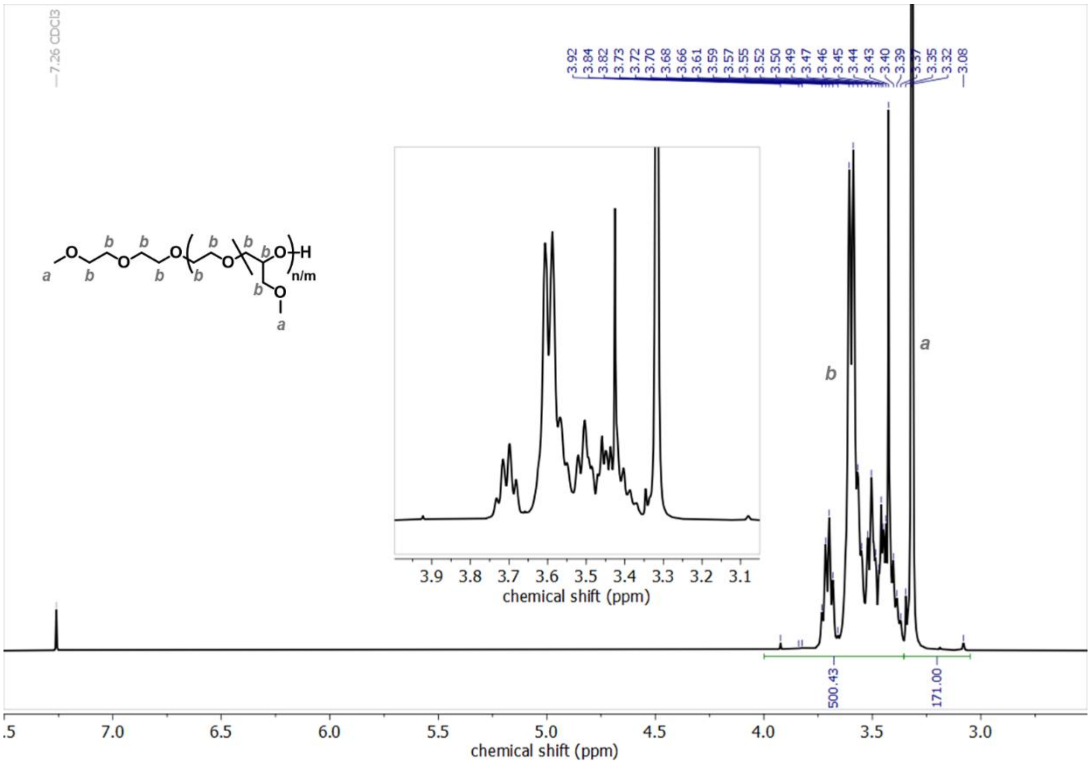

Figure S11. ^{1}H NMR spectrum ($CDCl_3$, 400 MHz) of $rPEG^{0.51}_{110}$.

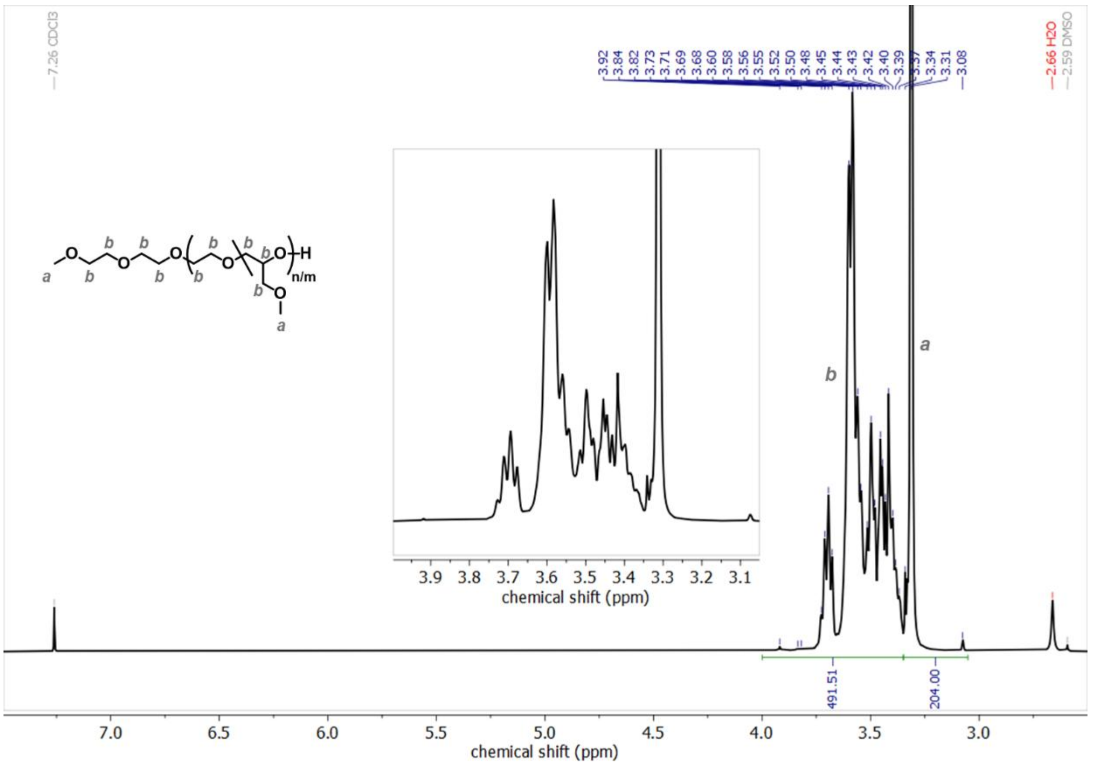

Figure S12. ^{1}H NMR spectrum ($CDCl_3$, 400 MHz) of $rPEG_{105}^{0.64}$.

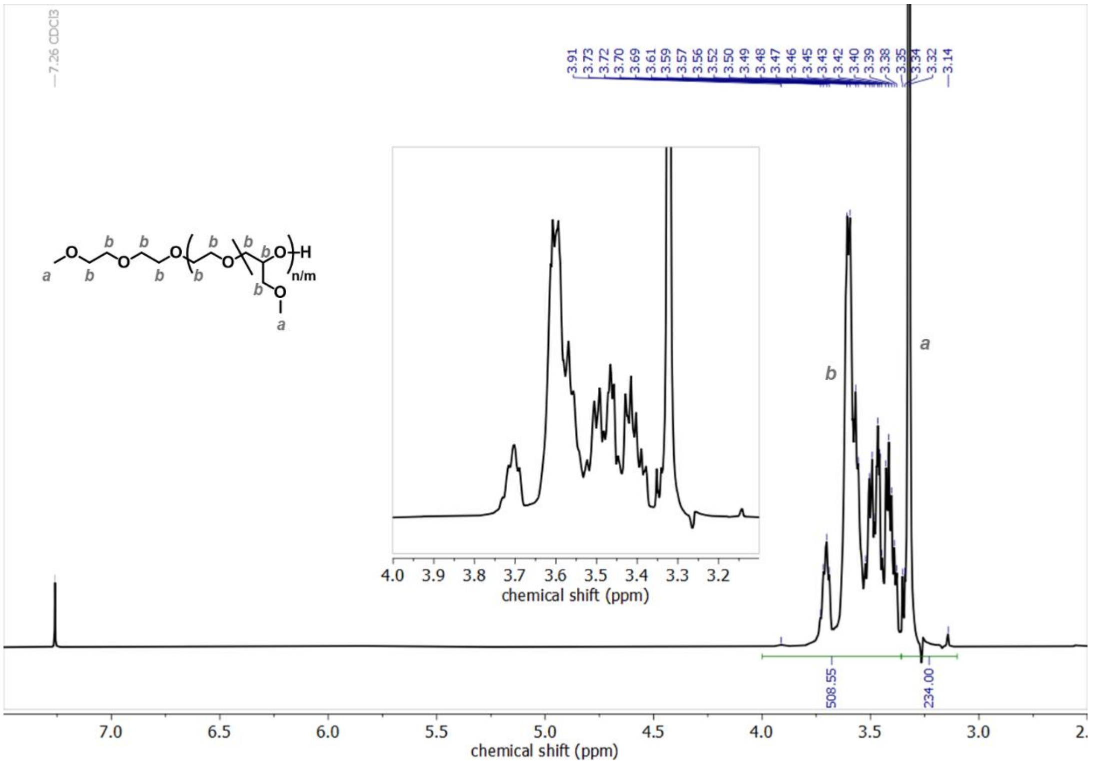

Figure S13. ^{1}H NMR spectrum ($CDCl_3$, 400 MHz) of $rPEG_{105}^{0.73}$.

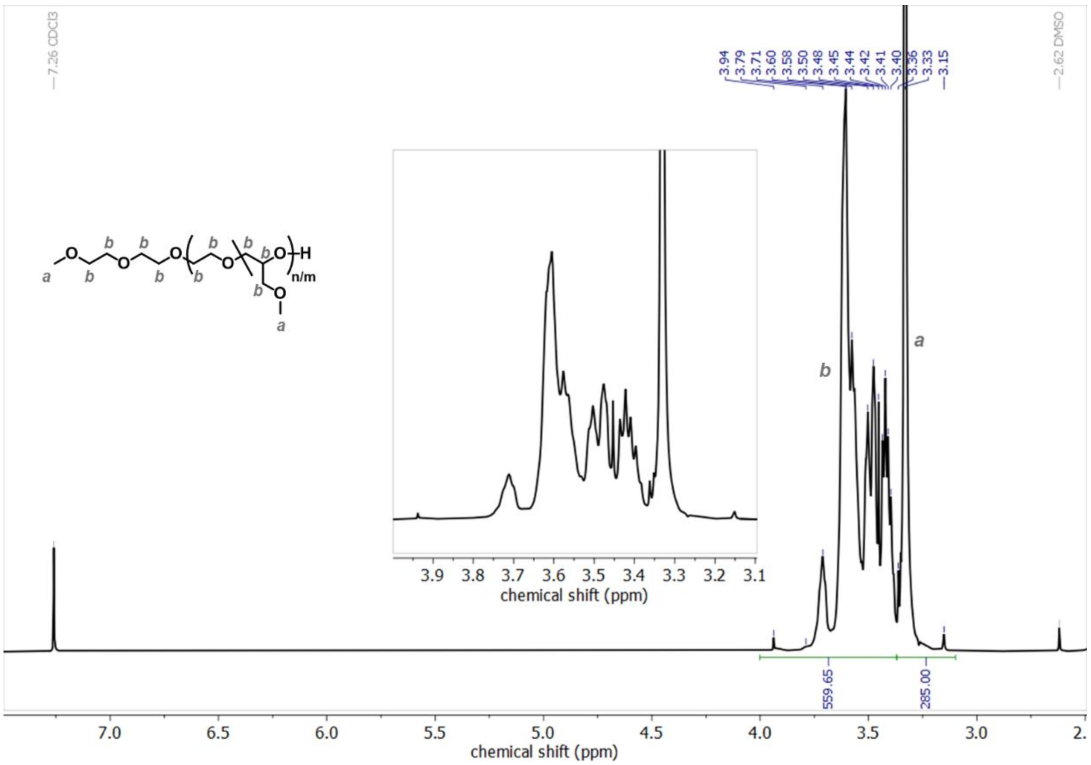

Figure S14. ^{1}H NMR spectrum ($CDCl_3$, 400 MHz) of $rPEG_{115}^{0.82}$.

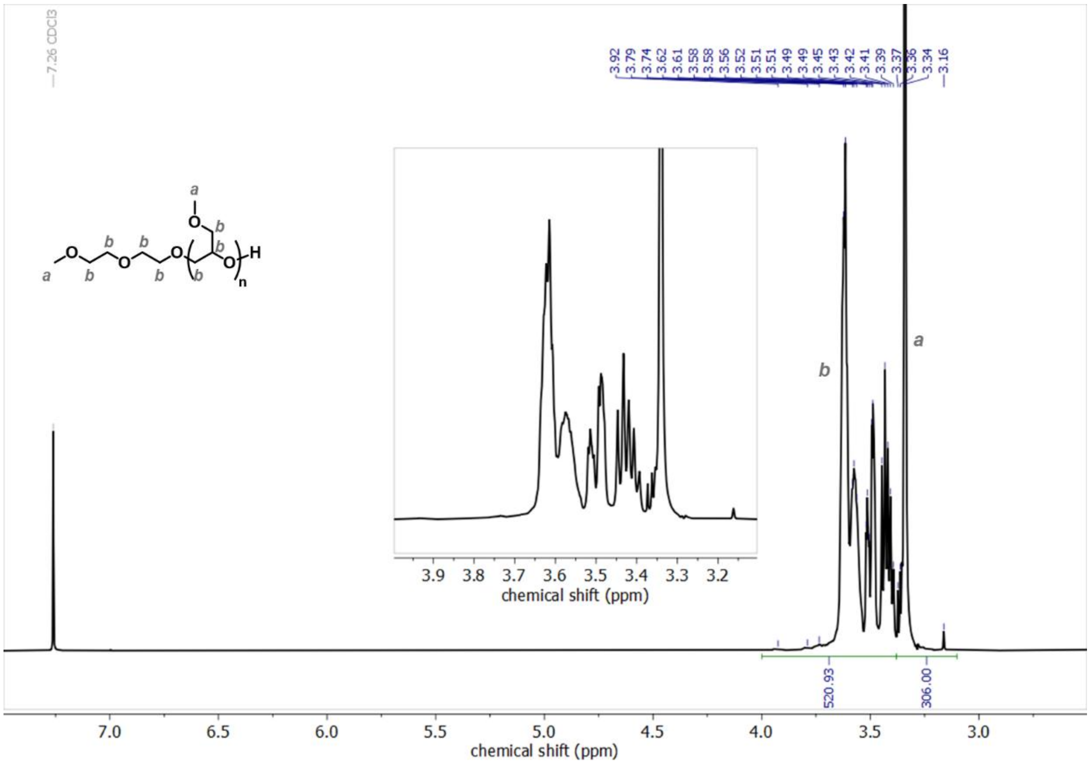

Figure S15. ^{1}H NMR spectrum ($CDCl_3$, 400 MHz) of $PGME_{103}$.

4. MALDI TOF mass spectra

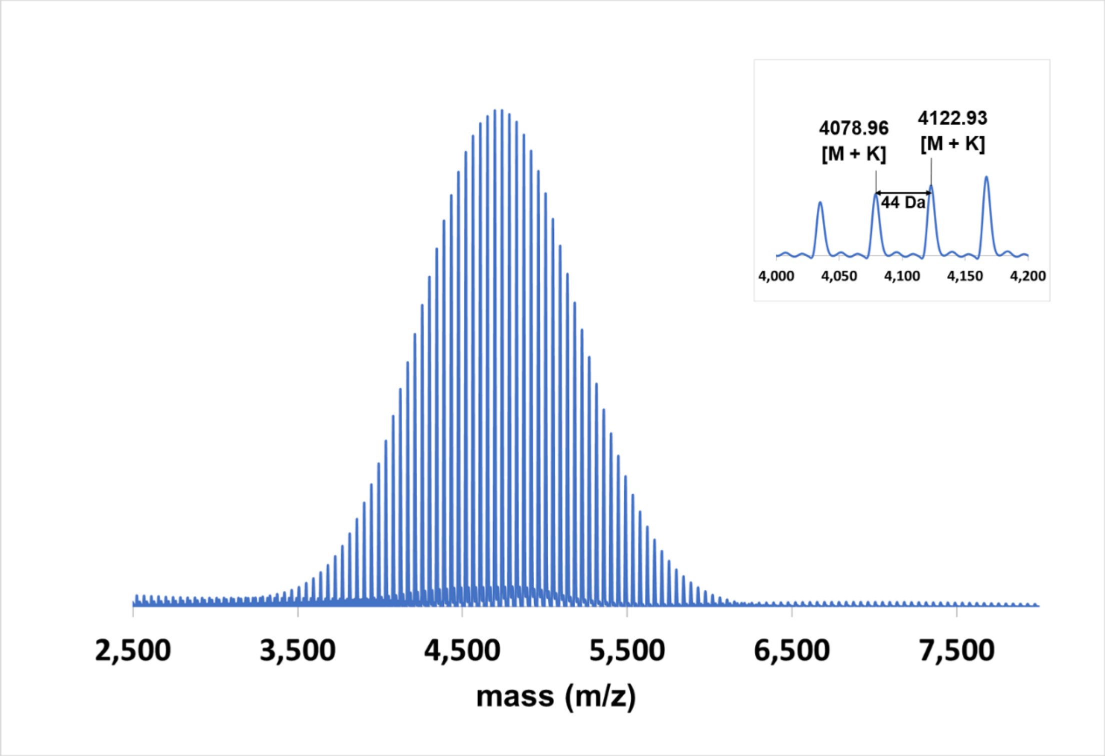

Figure S16. MALDI TOF mass spectrum of $mPEG_{106}$.

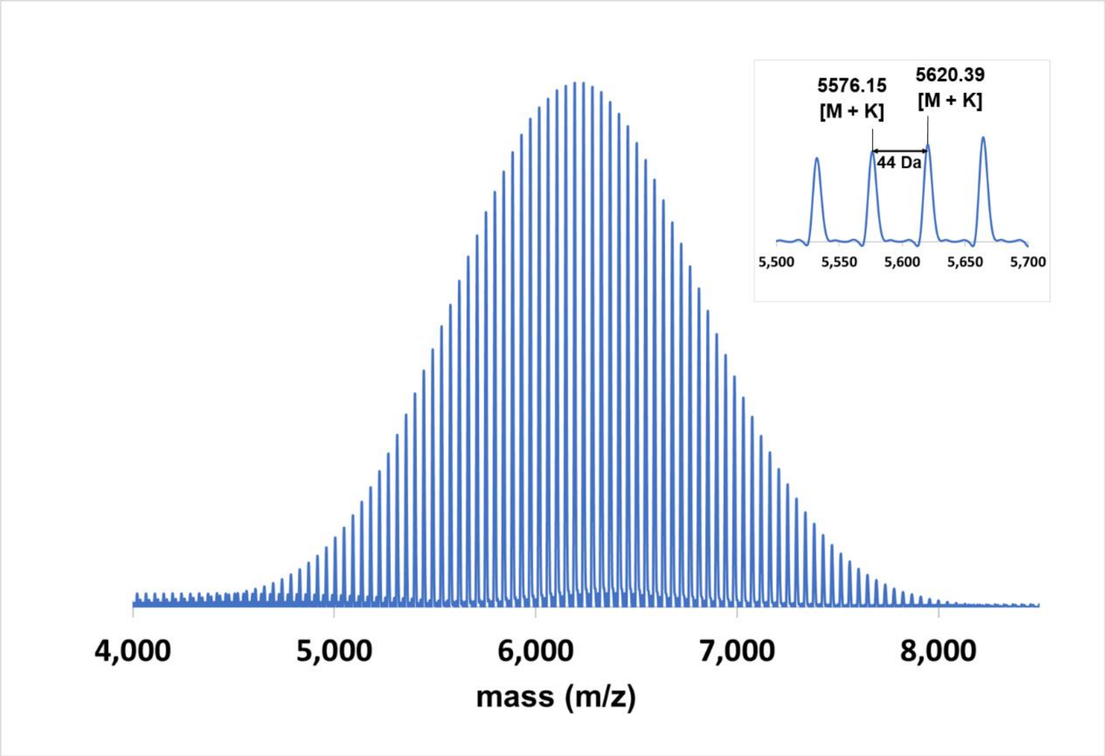

Figure S17. MALDI TOF mass spectrum of $rPEG_{111}^{0.26}$.

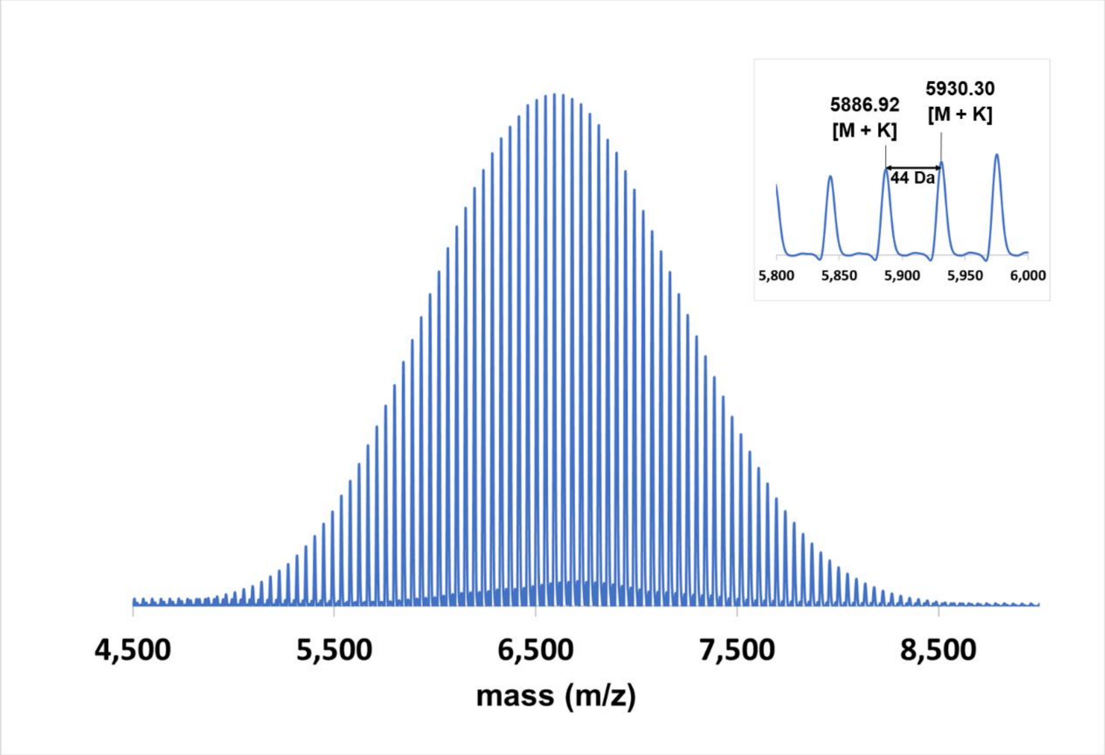

Figure S18. MALDI TOF mass spectrum of $rPEG_{111}^{0.35}$.

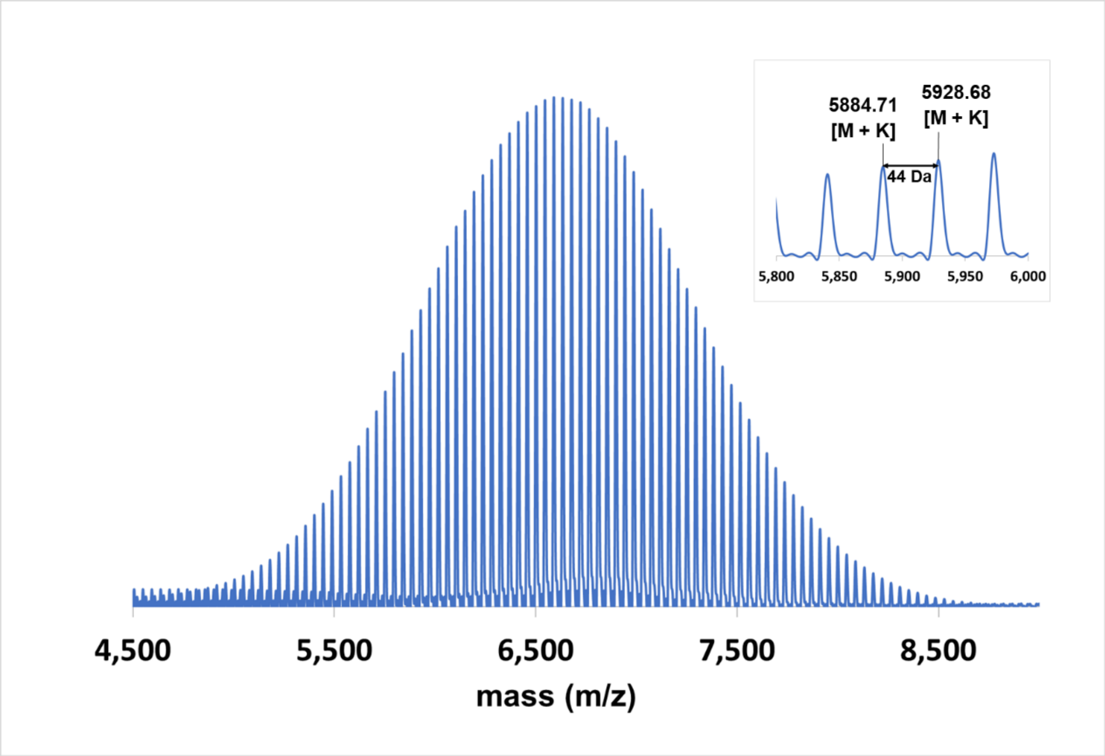

Figure S19. MALDI TOF mass spectrum of $rPEG_{106}^{0.41}$.

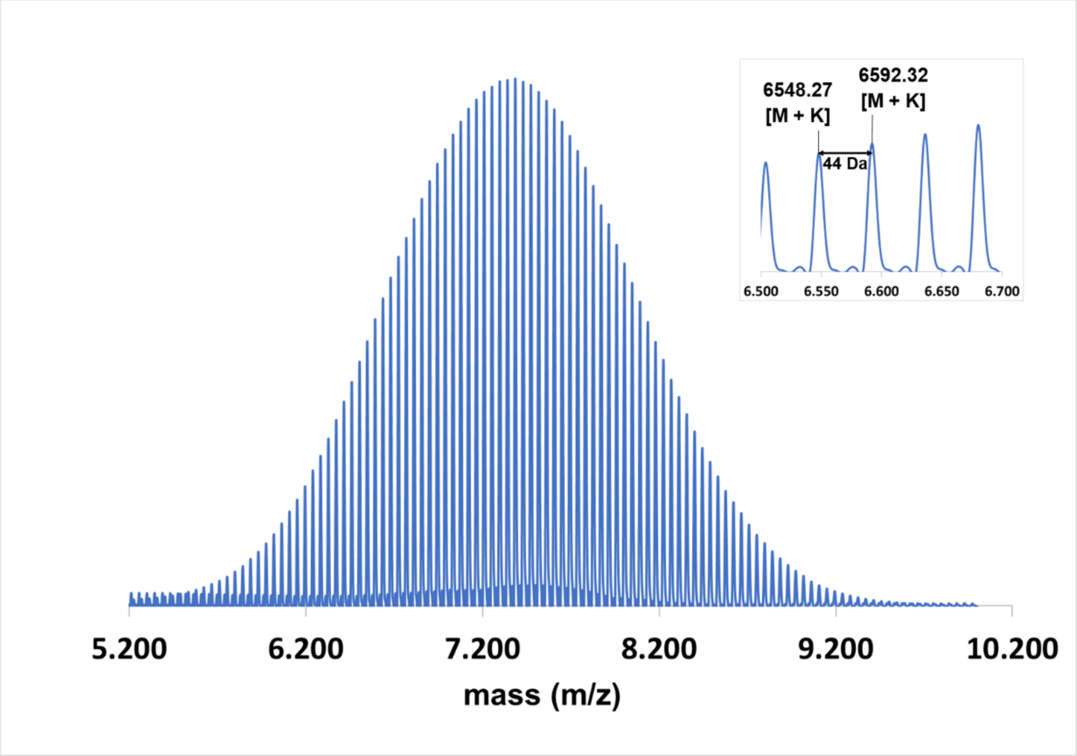

Figure S20. MALDI TOF mass spectrum of $\mathrm{rPEG}_{110}^{0.51}$.

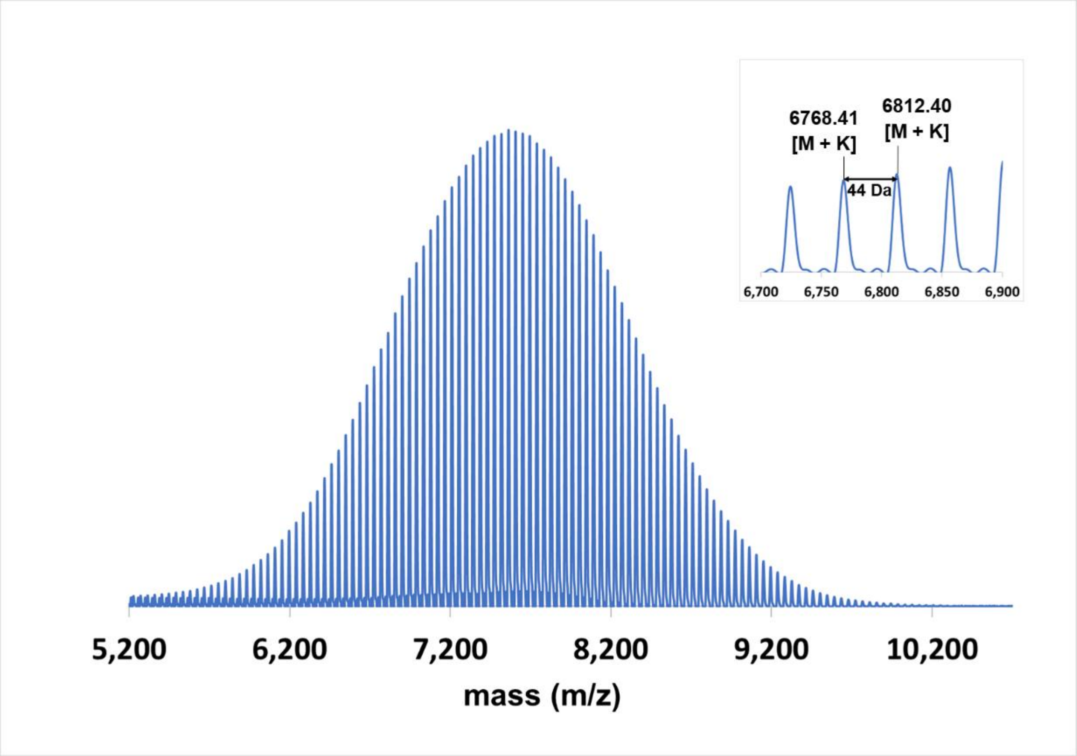

Figure S21. MALDI TOF mass spectrum of $\mathrm{rPEG}_{105}^{0.64}$.

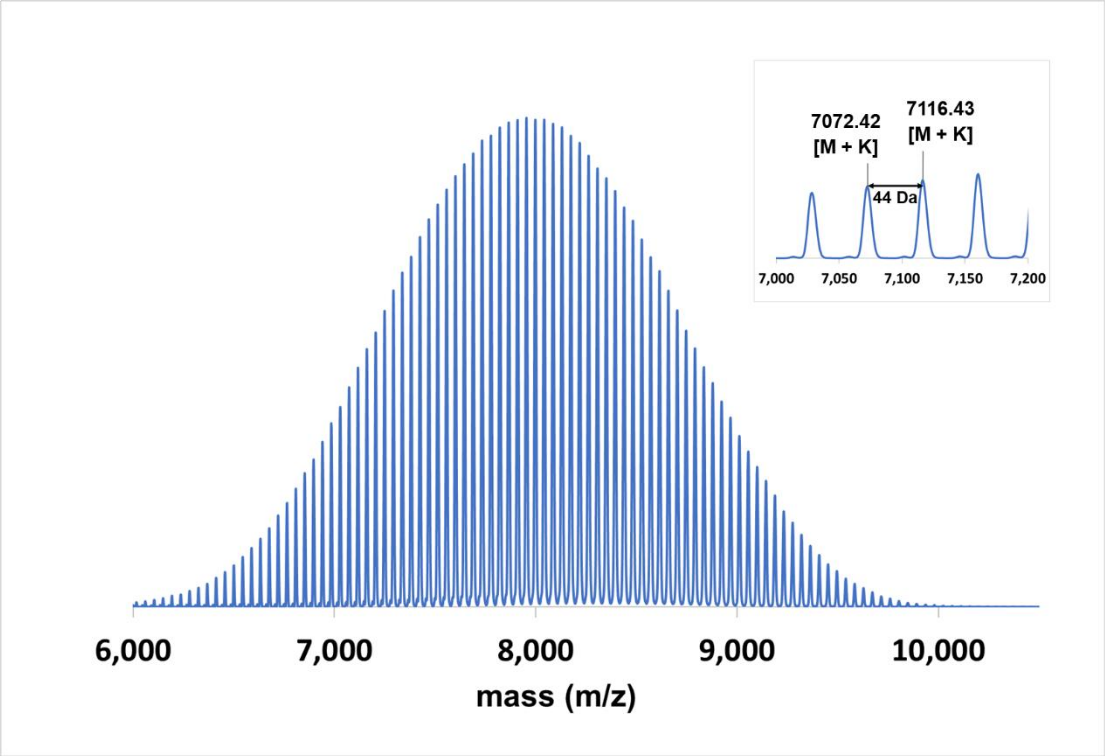

Figure S22. MALDI TOF mass spectrum of rPEG$_{105}^{0.73}$.

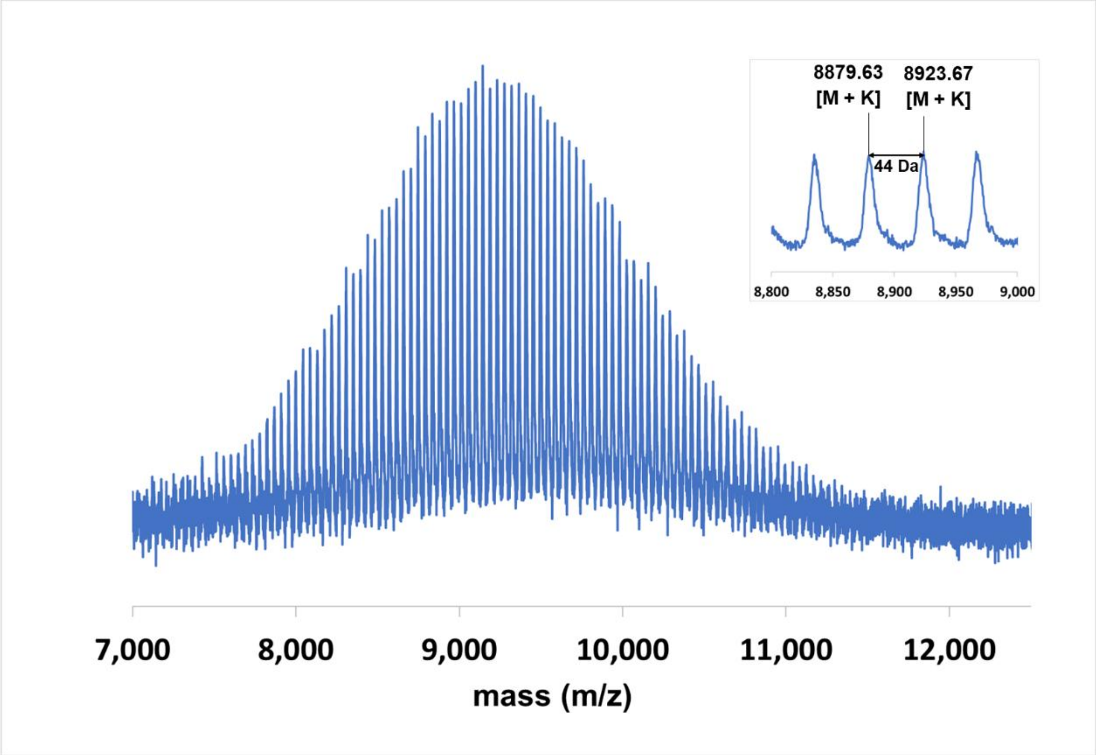

Figure S23. MALDI TOF mass spectrum of rPEG$_{115}^{0.82}$.

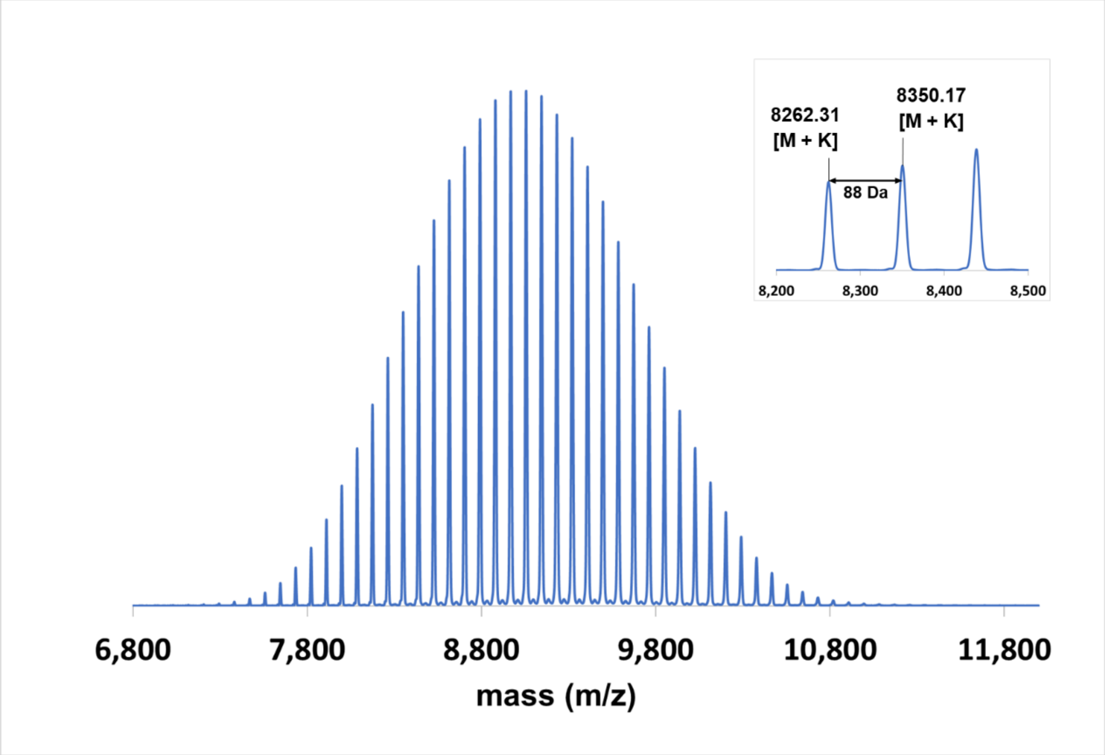

Figure S24. MALDI TOF mass spectrum of $PGME_{103}$.

5. Size Exclusion Chromatography (SEC)

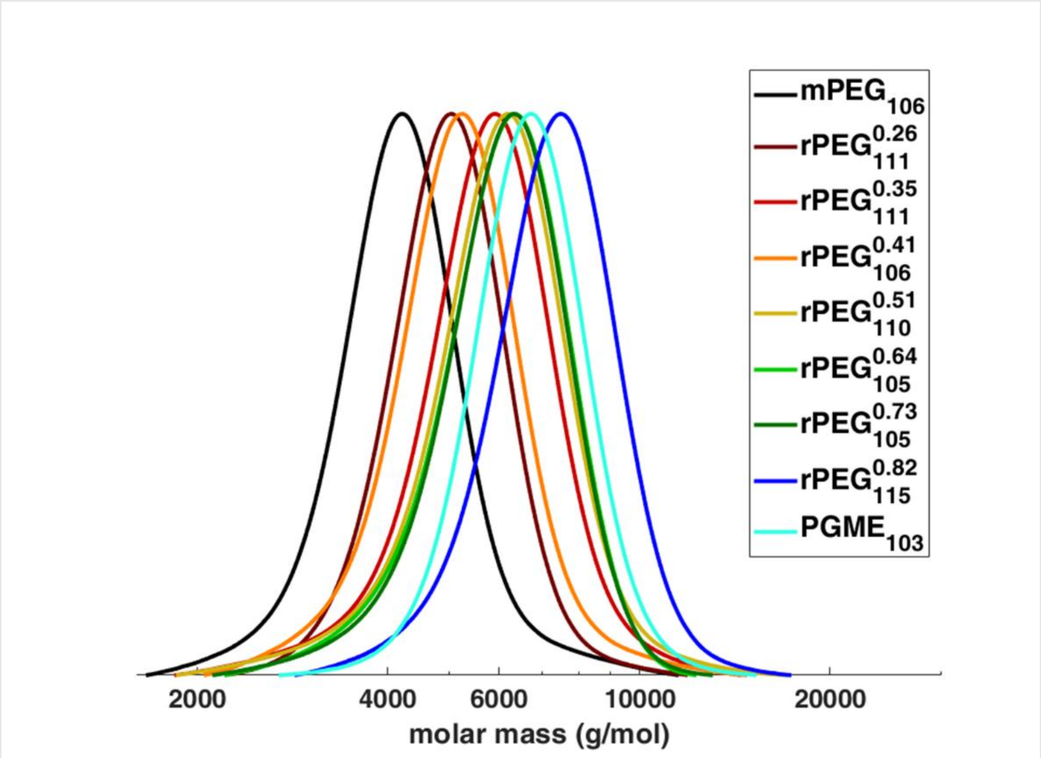

Figure S25. SEC curves (solvent: DMF; calibration: PEG) of the synthesized copolymers.

6. Cloud point measurements (turbidity curves)

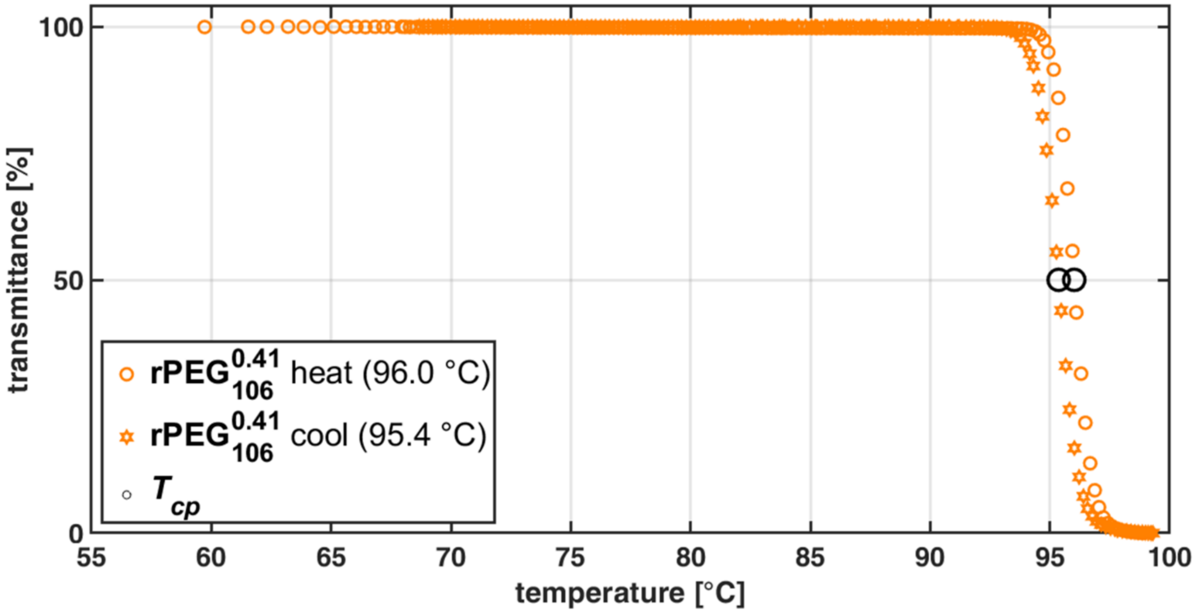

Figure S26. Turbidity curves (heating and cooling) of rPEG$^{0.41}_{106}$ measured at 5 mg mL^{-1} in Millipore water. Cloud points at 50 % transmittance are marked in the graph.

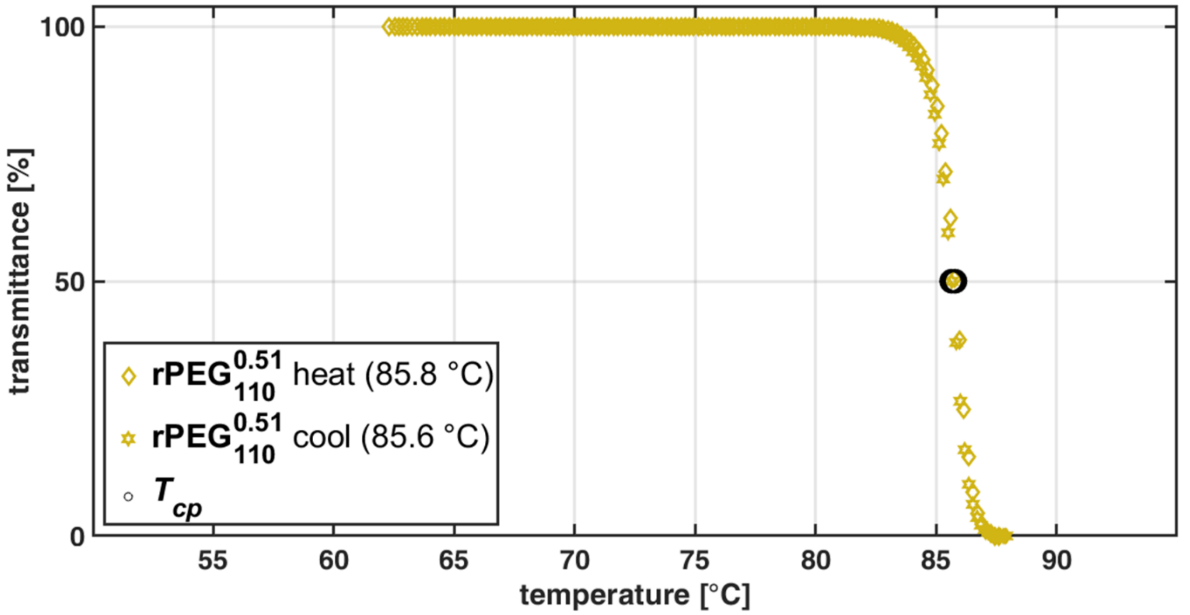

Figure S27. Turbidity curves (heating and cooling) of rPEG$^{0.51}_{110}$ measured at 5 mg mL^{-1} in Millipore water. Cloud points at 50 % transmittance are marked in the graph.

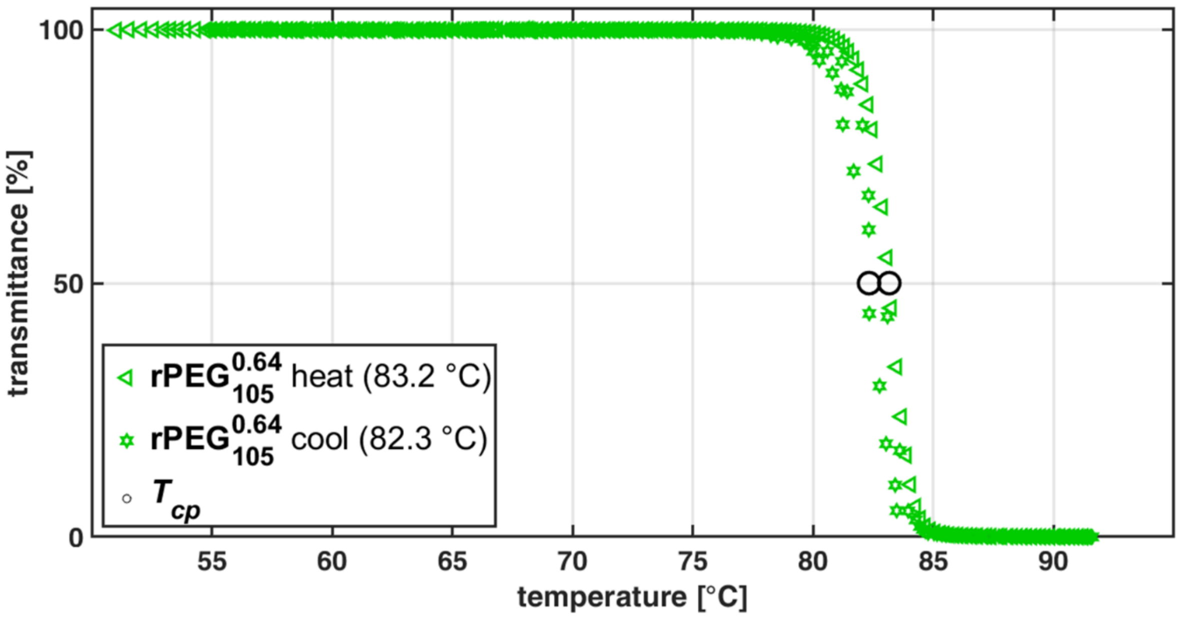

Figure S28. Turbidity curves (heating and cooling) of rPEG$^{0.64}_{105}$ measured at 5 mg mL^{-1} in Millipore water. Cloud points at 50 % transmittance are marked in the graph.

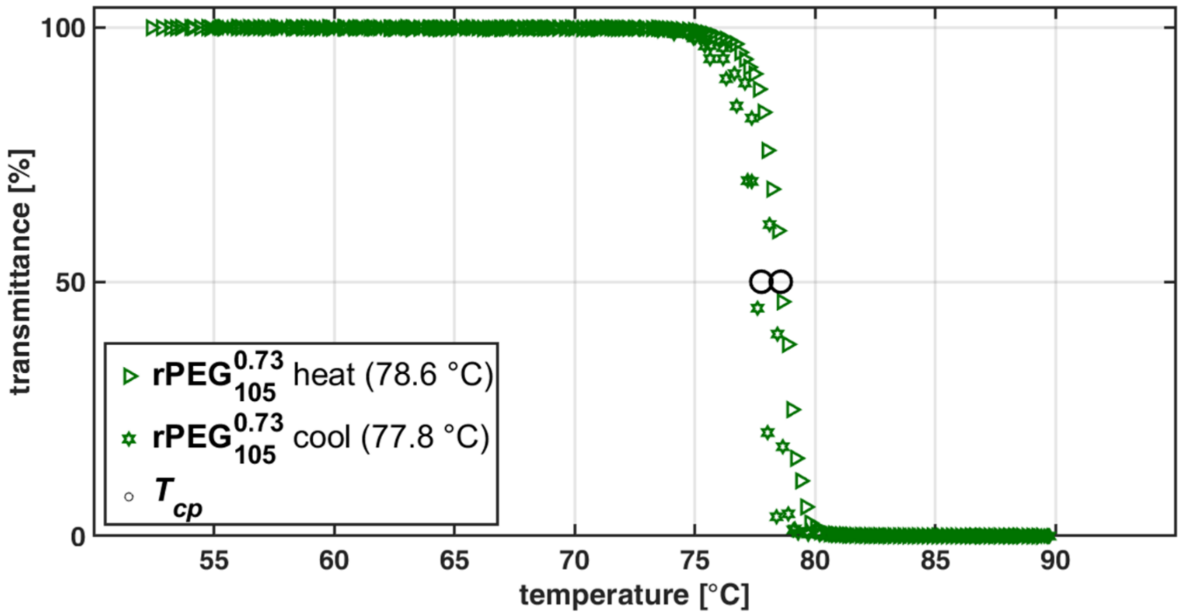

Figure S29. Turbidity curves (heating and cooling) of rPEG$^{0.73}_{105}$ measured at 5 mg mL^{-1} in Millipore water. Cloud points at 50 % transmittance are marked in the graph.

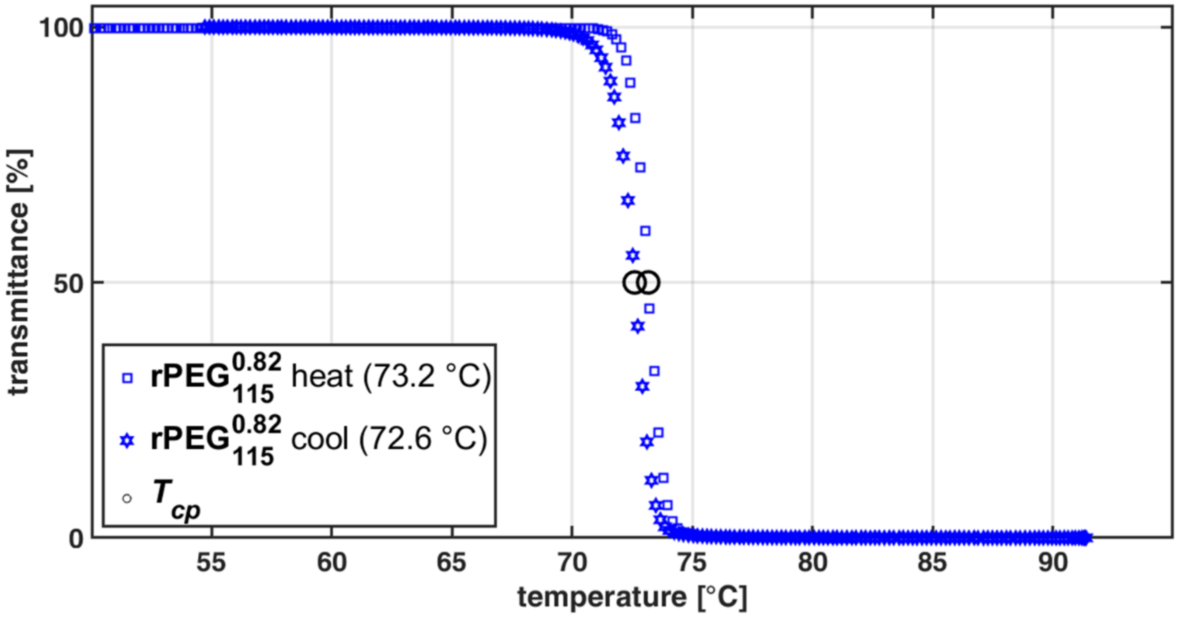

Figure S30. Turbidity curves (heating and cooling) of rPEG$^{0.82}_{115}$ measured at 5 mg mL^{-1} in Millipore water. Cloud points at 50 % transmittance are marked in the graph.

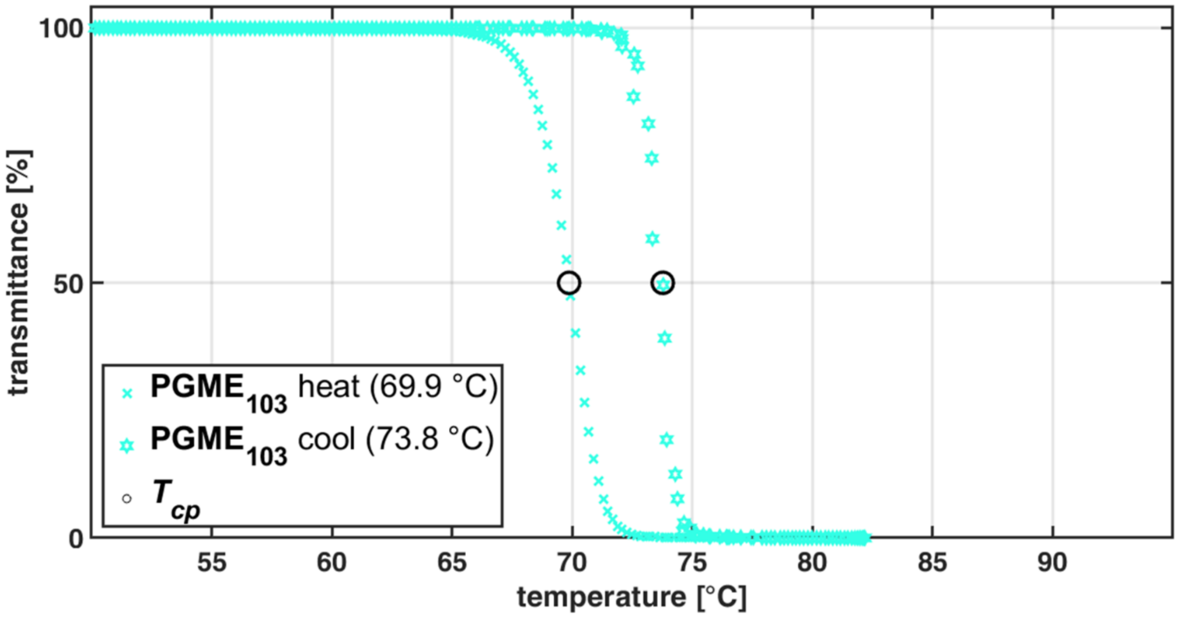

Figure S31. Turbidity curves (heating and cooling) of PGME$_{103}$ measured at 5 mg mL^{-1} in Millipore water. Cloud points at 50 % transmittance are marked in the graph.

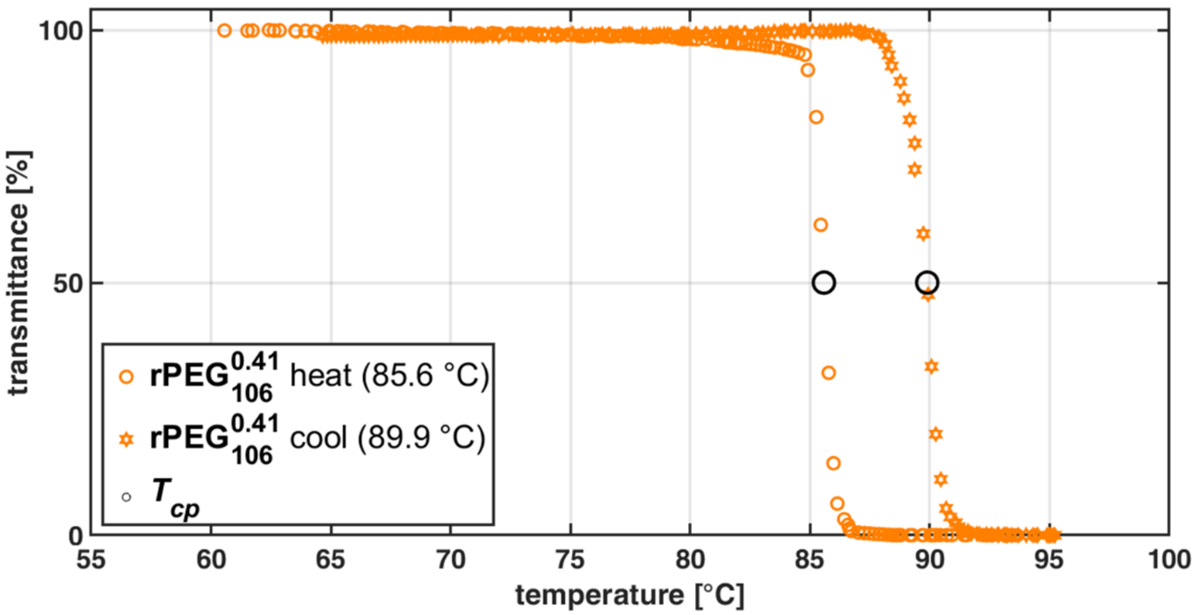

Figure S32. Turbidity curves (heating and cooling) of rPEG$^{0.41}_{106}$ measured at 100 mg mL^{-1} in Millipore water. Cloud points at 50 % transmittance are marked in the graph.

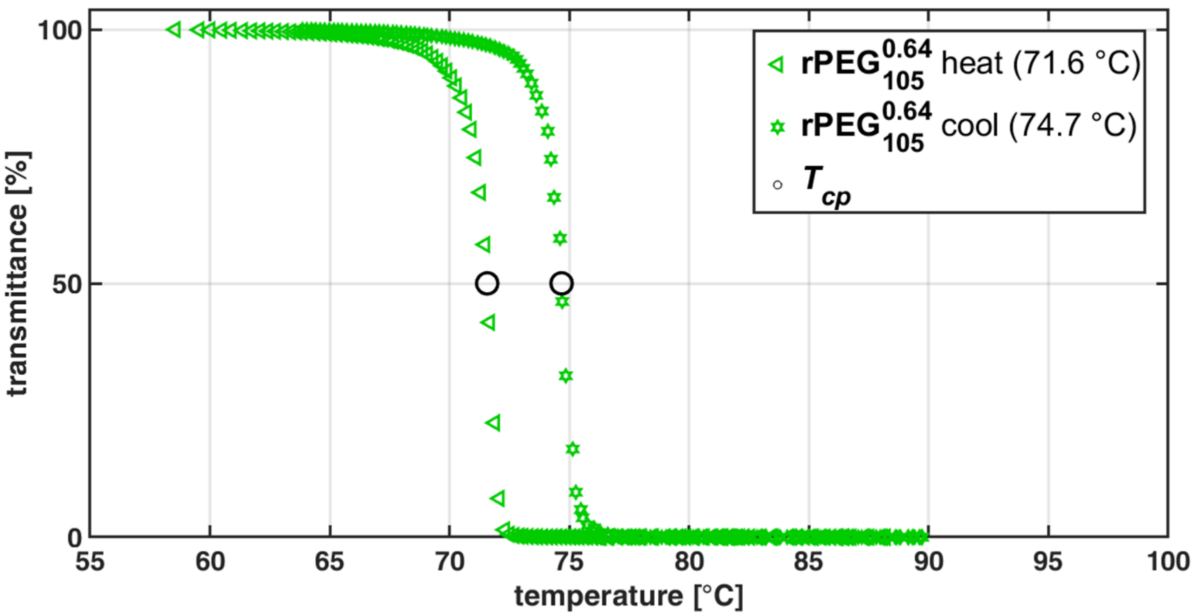

Figure S 33. Turbidity curves (heating and cooling) of rPEG$^{0.64}_{105}$ measured at 100 mg mL^{-1} in Millipore water. Cloud points at 50 % transmittance are marked in the graph.

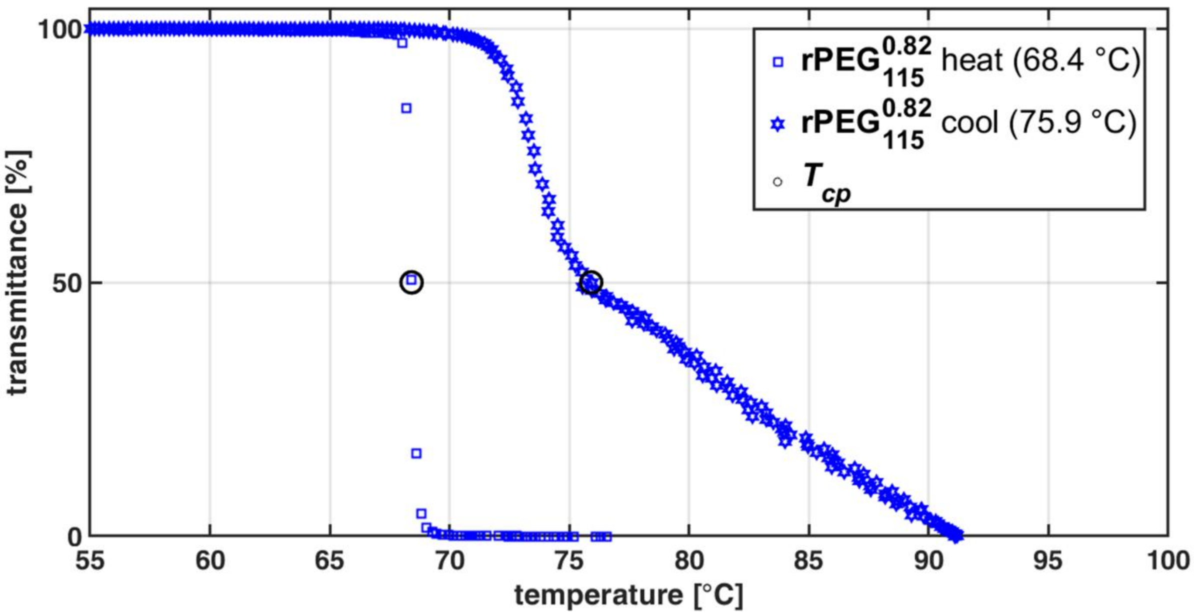

Figure S 34. Turbidity curves (heating and cooling) of $rPEG^{0.82}_{115}$ measured at 100 mg mL^{-1} in Millipore water. Cloud points at 50 % transmittance are marked in the graph.

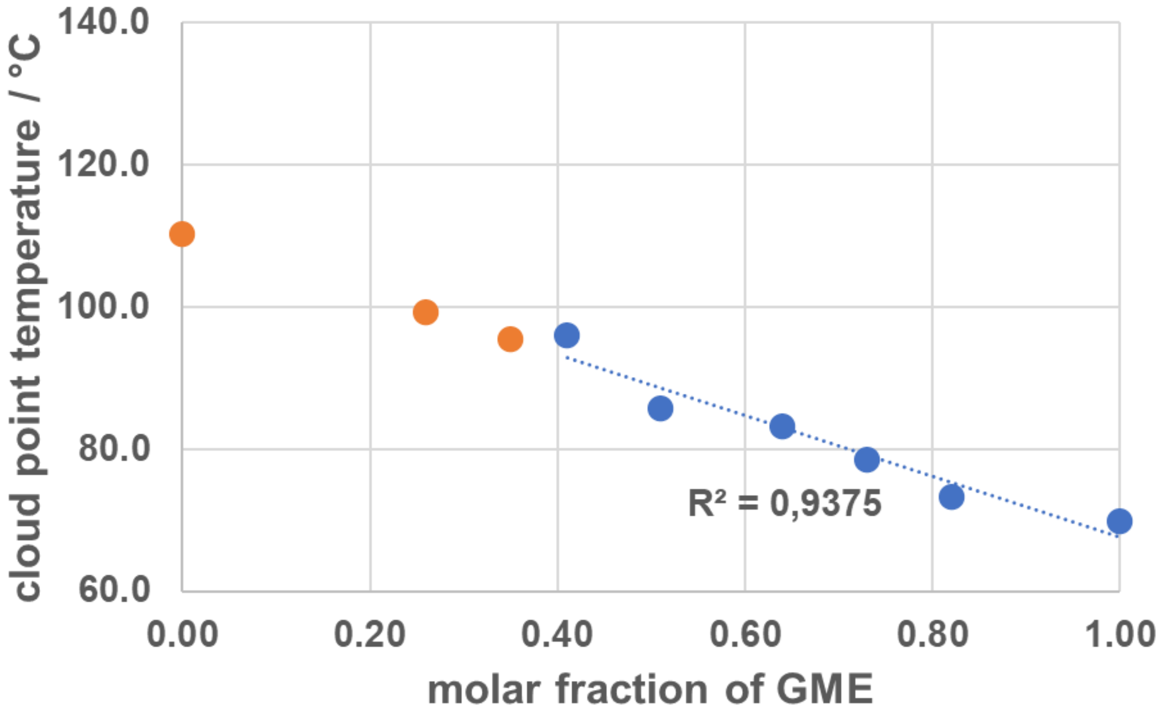

Figure S35. Molar fraction of GME in rPEGs plotted against cloud point temperatures for conc. = 5 mg mL^{-1} (blue = measured values; orange = predicted values based on fit); linear fit of measured cloud points and predicted values based on this fit. Linear fit equation: $\boldsymbol{T_{cp}}(\boldsymbol{f_{GME}}) = -42.69 \cdot \boldsymbol{f_{GME}} + 110.36$.

Table S1. Predicted cloud point temperatures (orange) based on a linear fit of measured values (blue) at conc. = 5 mg mL^{-1}.

**Polymer**	T_{cp} (for conc. = 5 mg mL^{-1}) / °C
**mPEG$_{106}$**	110.4
**rPEG$^{0.26}_{111}$**	99.3
**rPEG$^{0.35}_{111}$**	95.4
**rPEG$^{0.41}_{106}$**	96.0
**rPEG$^{0.51}_{110}$**	85.8
**rPEG$^{0.64}_{105}$**	83.2
**rPEG$^{0.73}_{105}$**	78.6
**rPEG$^{0.82}_{115}$**	73.2
**PGME$_{103}$**	69.9

Following a linear trend, the T_{cp} of mPEG was predicted to be 110.4 °C. This is in contrast to previous publications where the T_{cp} of mPEG of similar molar mass and concentration was found at approximately 130 °C.[12] Therefore, a polynomial fit was applied which fit well for all measured cloud points – including the one taken from literature (see main manuscript).

7. DSC curves

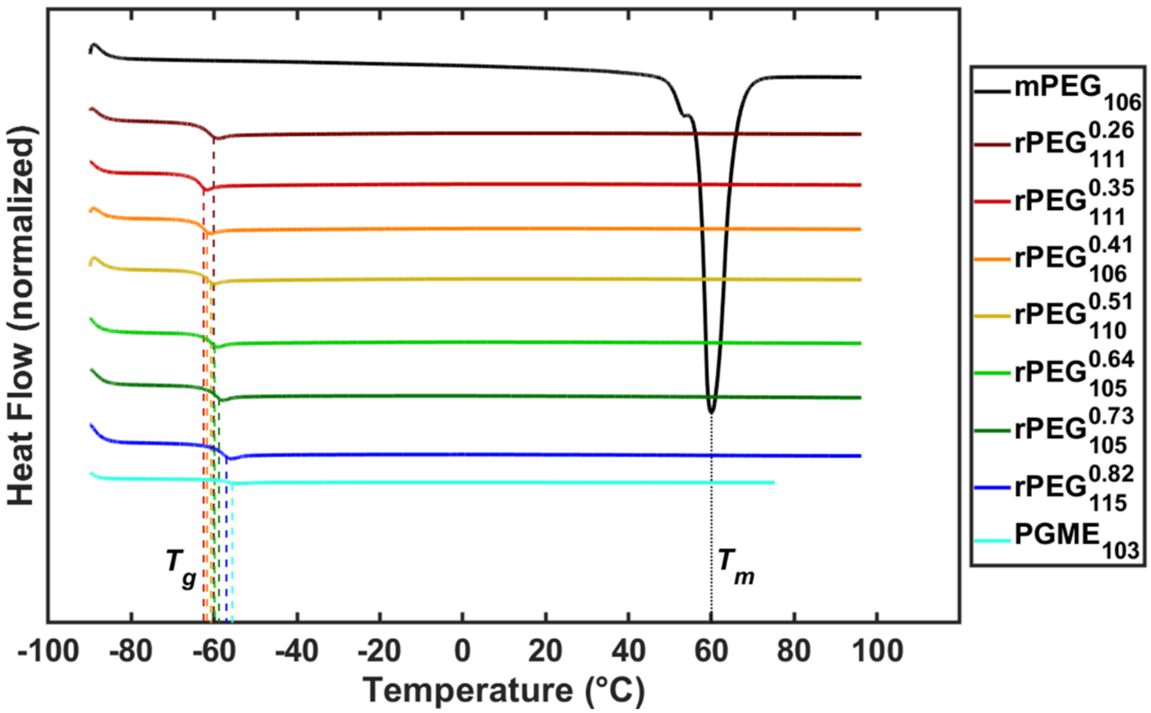

Figure S36. DSC curves of the synthesized copolymers. Glass transition temperatures T_g and melting temperatures T_m are marked in the graph.

Table S2. Glass transition temperatures T_g and melting temperatures T_m of the synthesized copolymers.

Polymer	M_n (MALDI TOF) / g mol^{-1}	T_g / ° C	T_m / ° C	ΔH_m / J g^{-1}
**mPEG$_{106}$**	4,724	--	60	169.1
**rPEG$^{0.26}_{111}$**	6,217	– 60	--	--
**rPEG$^{0.35}_{111}$**	6,617	– 63	--	--
**rPEG$^{0.41}_{106}$**	6,629	– 62	--	--
**rPEG$^{0.51}_{110}$**	7,384	– 61	--	--
**rPEG$^{0.64}_{105}$**	7,593	– 60	--	--
**rPEG$^{0.73}_{105}$**	8,007	– 59	--	--
**rPEG$^{0.82}_{115}$**	9,292	– 57	--	--
**PGME$_{103}$**	9,085	– 56	--	--

8. EPR Spectroscopy

8.1. Experimental and simulated spectra

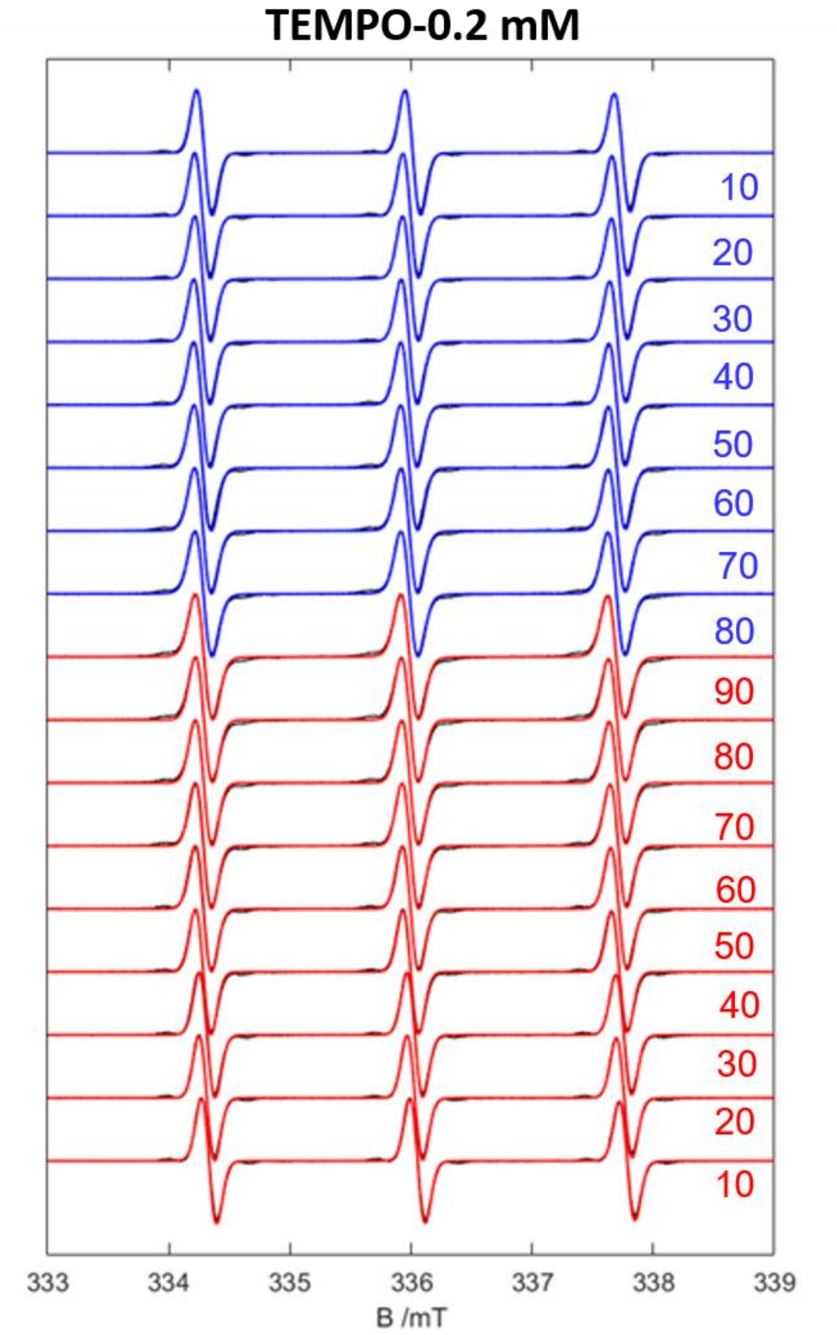

Figure S37. Experimental data (black) and corresponding simulation of EPR spectra of spin probe TEMPO (0.02 mM) for varying temperatures. Simulated spectra in heating and cooling cycles are shown in red and blue. For the purpose of clarity, only a selection of spectra is shown.

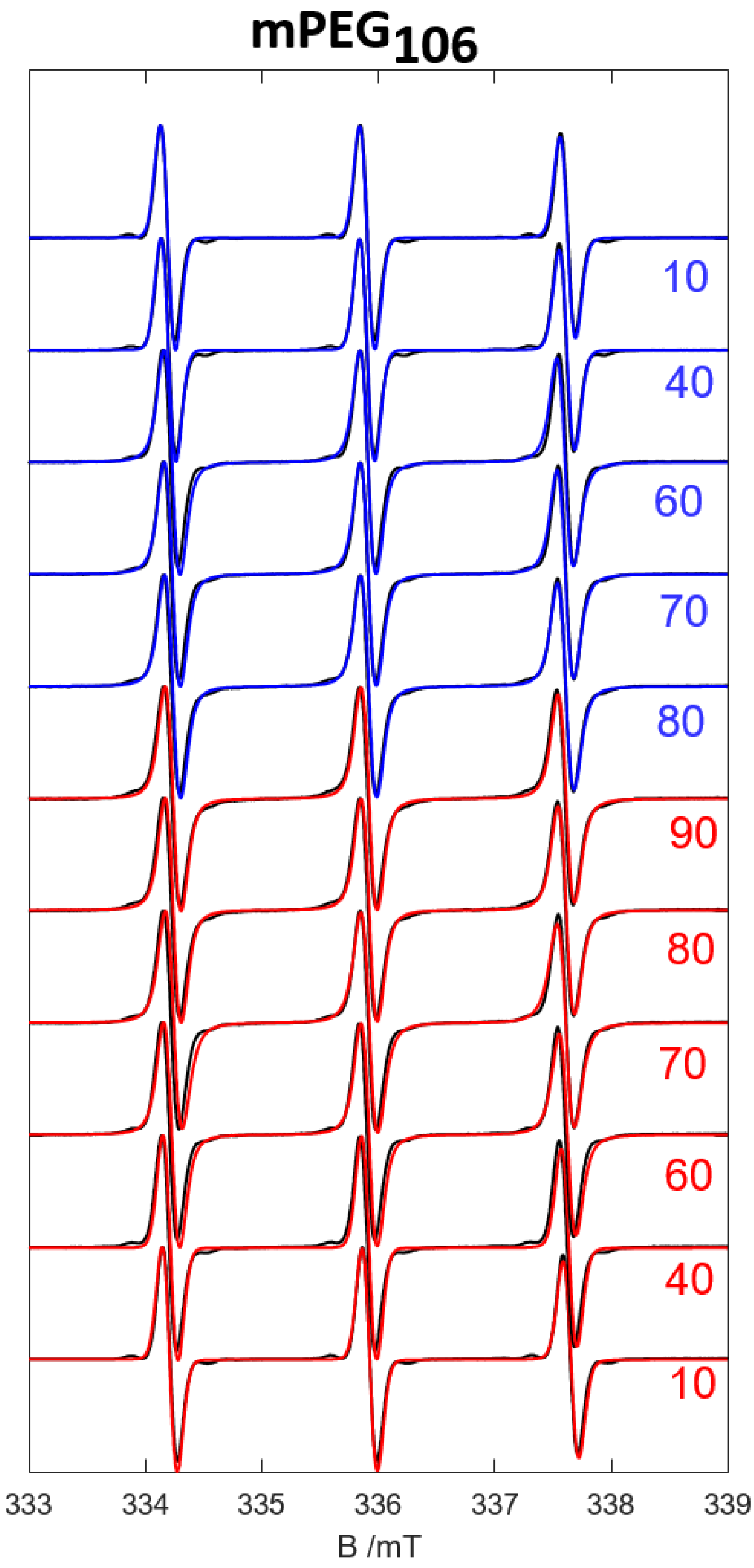

Figure S38. Experimental data (black) and corresponding simulation of EPR spectra of a 10 w% aqueous solution of $mPEG_{106}$ (1.00 mM TEMPO) for varying temperatures. Simulated spectra in heating and cooling cycles are shown in red and blue. For the purpose of clarity, only a selection of spectra is shown.

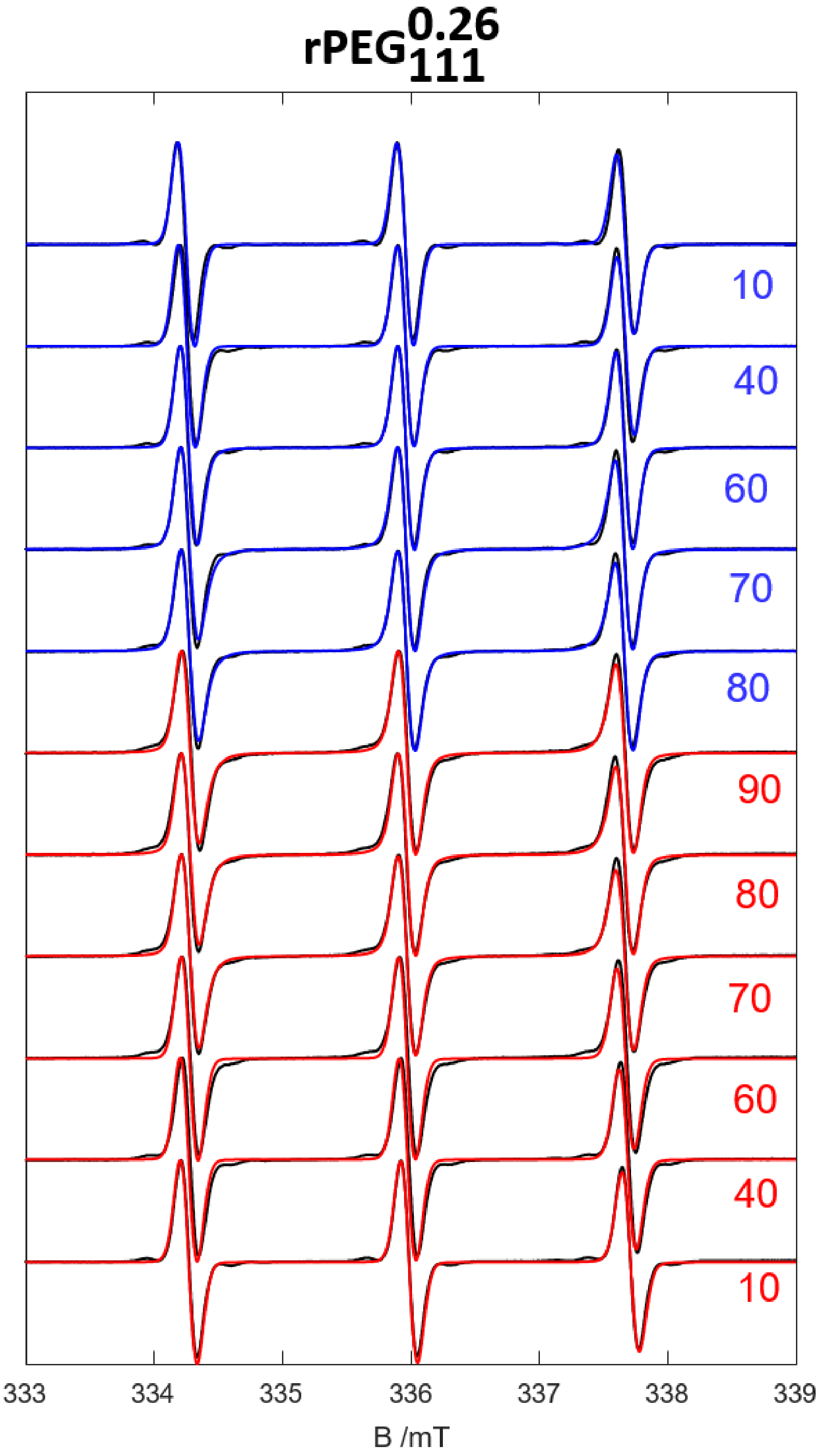

Figure S39. Experimental data (black) and corresponding simulation of EPR spectra of a 10 w% aqueous solution of $rPEG_{111}^{0.26}$ (1.00 mM TEMPO) for varying temperatures. Simulated spectra in heating and cooling cycles are shown in red and blue. For the purpose of clarity, only a selection of spectra is shown.

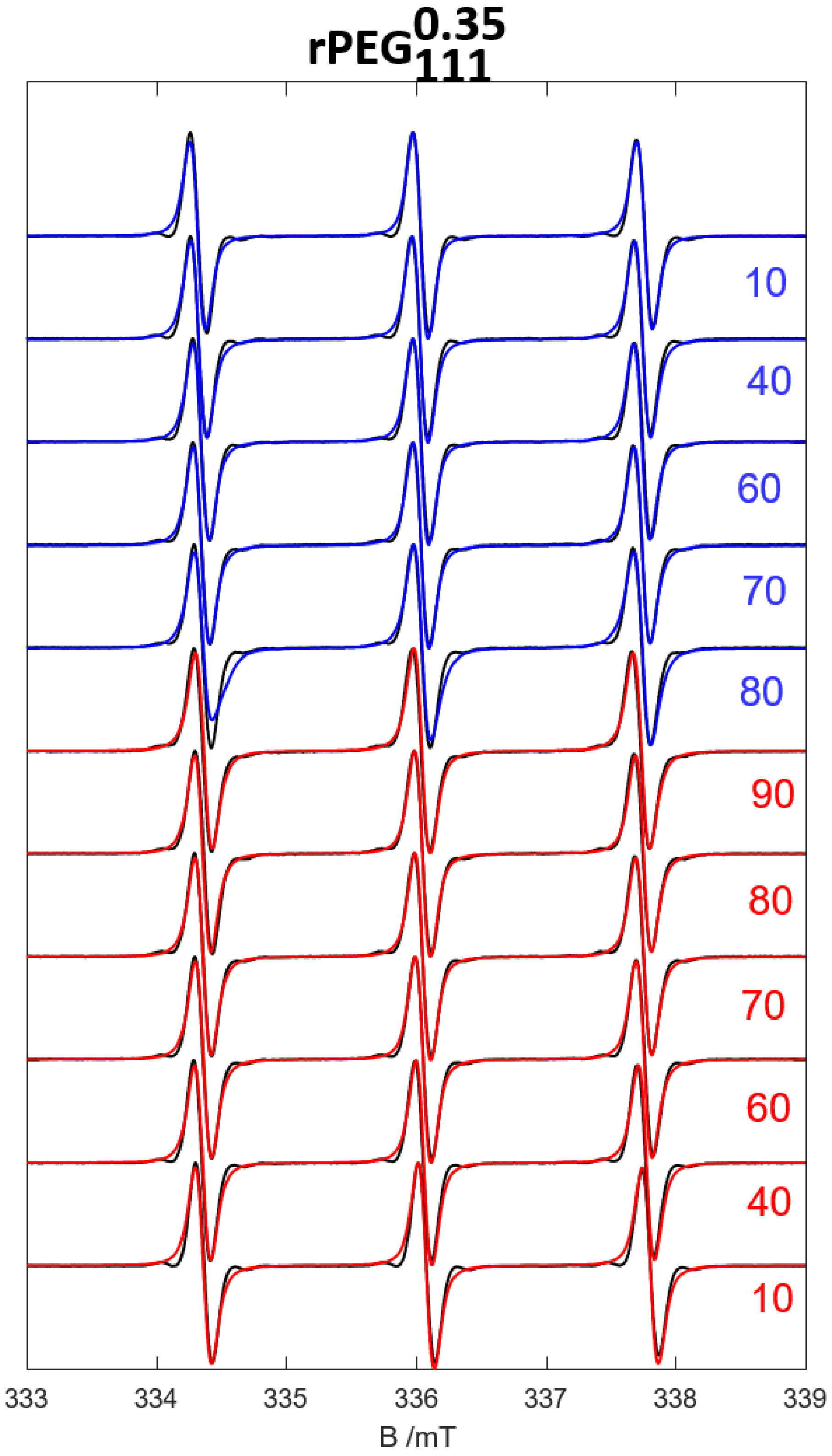

Figure S40. Experimental data (black) and corresponding simulation of EPR spectra of a 10 w% aqueous solution of $rPEG_{111}^{0.35}$ (1.00 mM TEMPO) for varying temperatures. Simulated spectra in heating and cooling cycles are shown in red and blue. For the purpose of clarity, only a selection of spectra is shown.

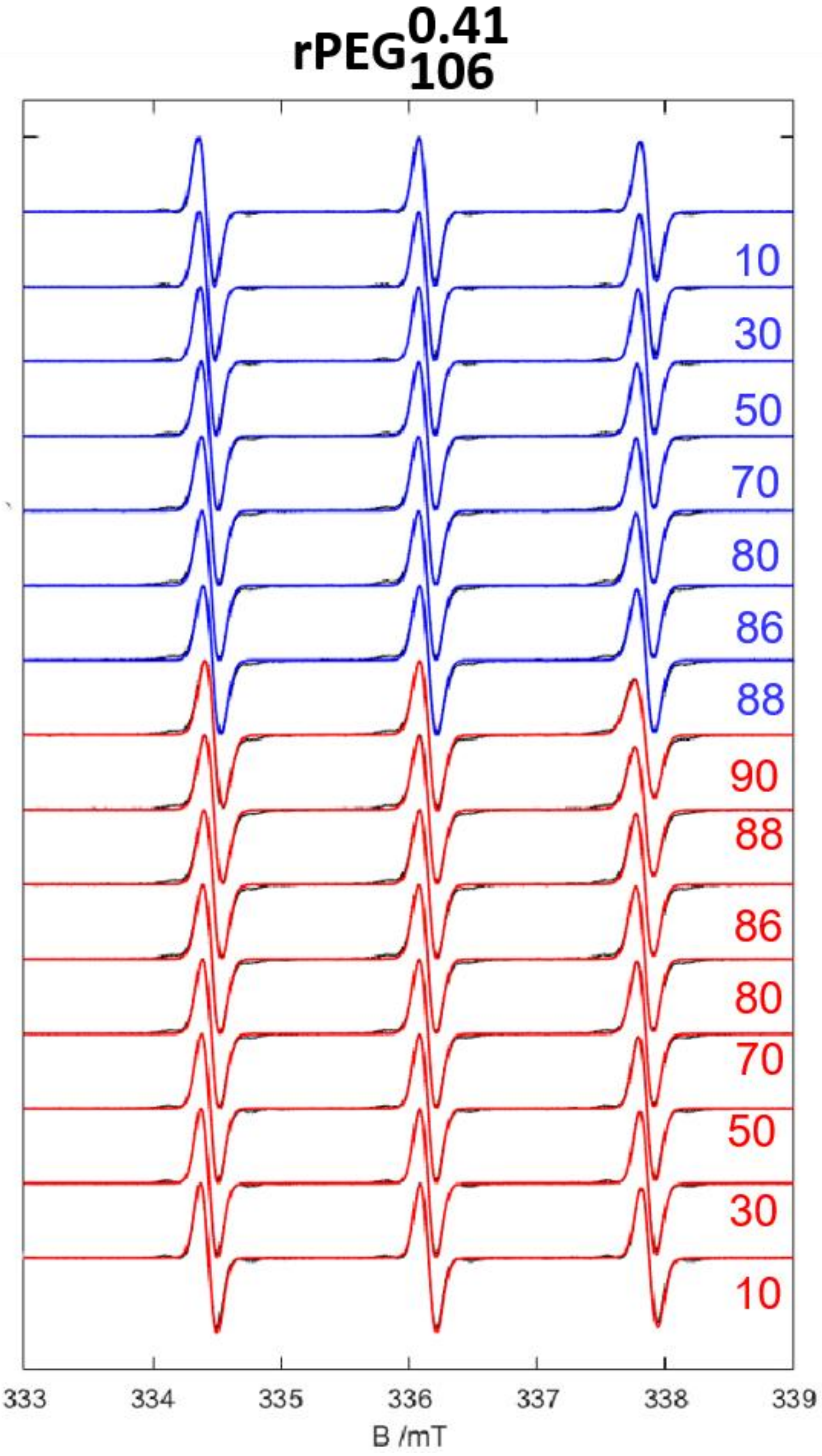

Figure S41. Experimental data (black) and corresponding simulation of EPR spectra of a 10 w% aqueous solution of $rPEG^{0.41}_{106}$ (1.00 mM TEMPO) for varying temperatures. Simulated spectra in heating and cooling cycles are shown in red and blue. For the purpose of clarity, only a selection of spectra is shown.

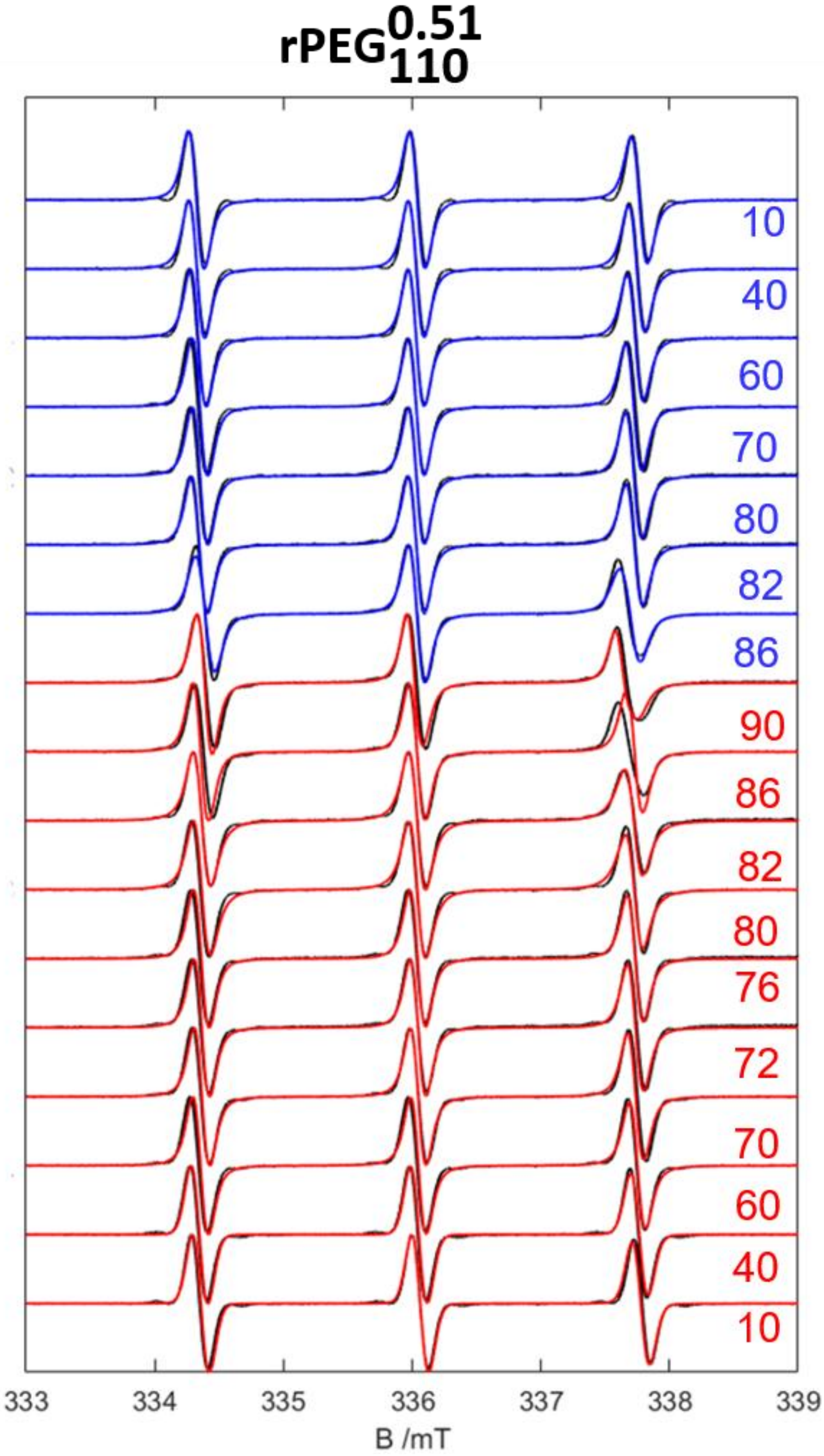

Figure S42. Experimental data (black) and corresponding simulation of EPR spectra of a 10 w% aqueous solution of $rPEG^{0.51}_{110}$ (1.00 mM TEMPO) for varying temperatures. Simulated spectra in heating and cooling cycles are shown in red and blue. For the purpose of clarity, only a selection of spectra is shown.

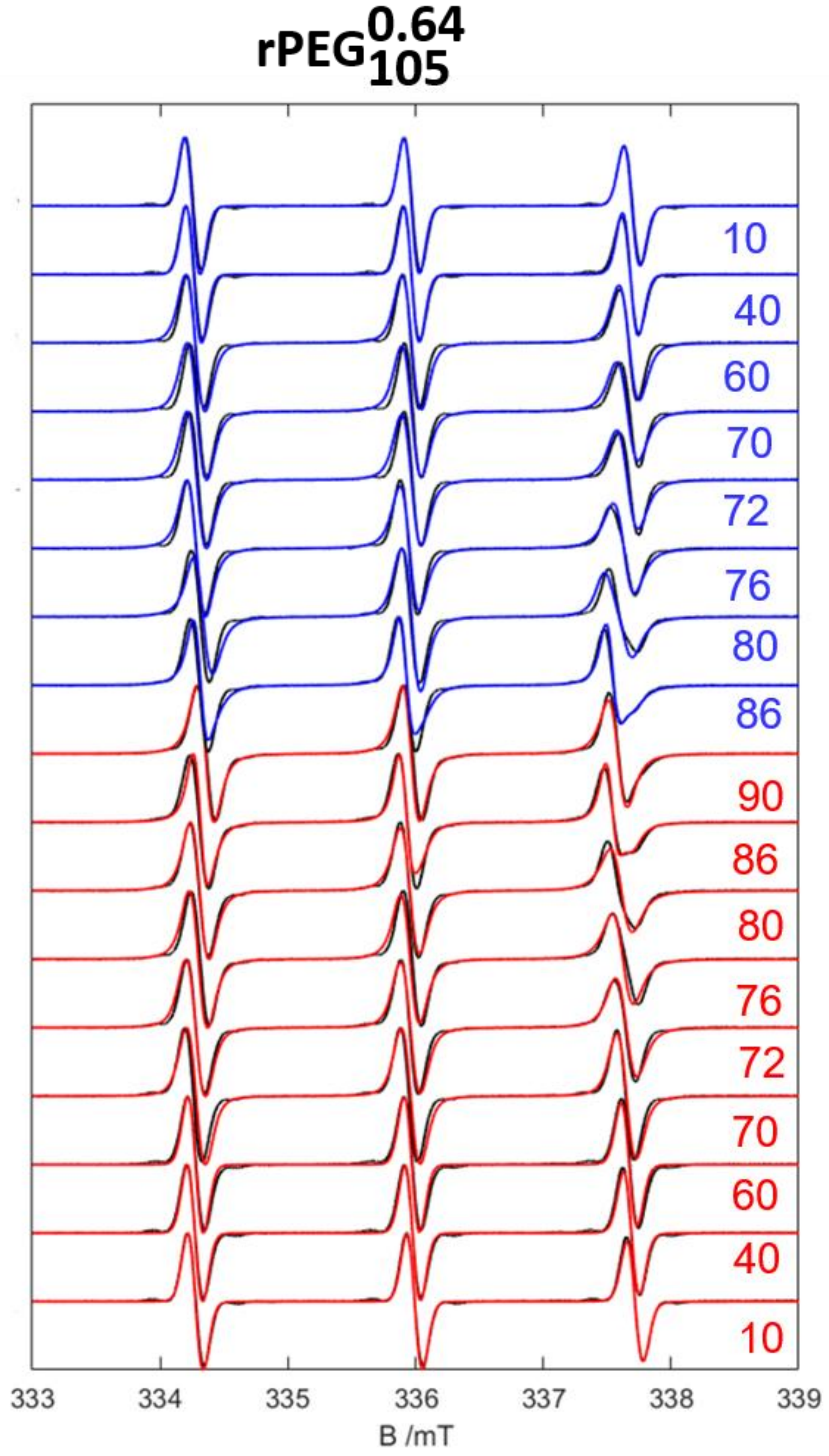

Figure S43. Experimental data (black) and corresponding simulation of EPR spectra of a 10 w% aqueous solution of rPEG$^{0.64}_{105}$ (1.00 mM TEMPO) for varying temperatures. Simulated spectra in heating and cooling cycles are shown in red and blue. For the purpose of clarity, only a selection of spectra is shown.

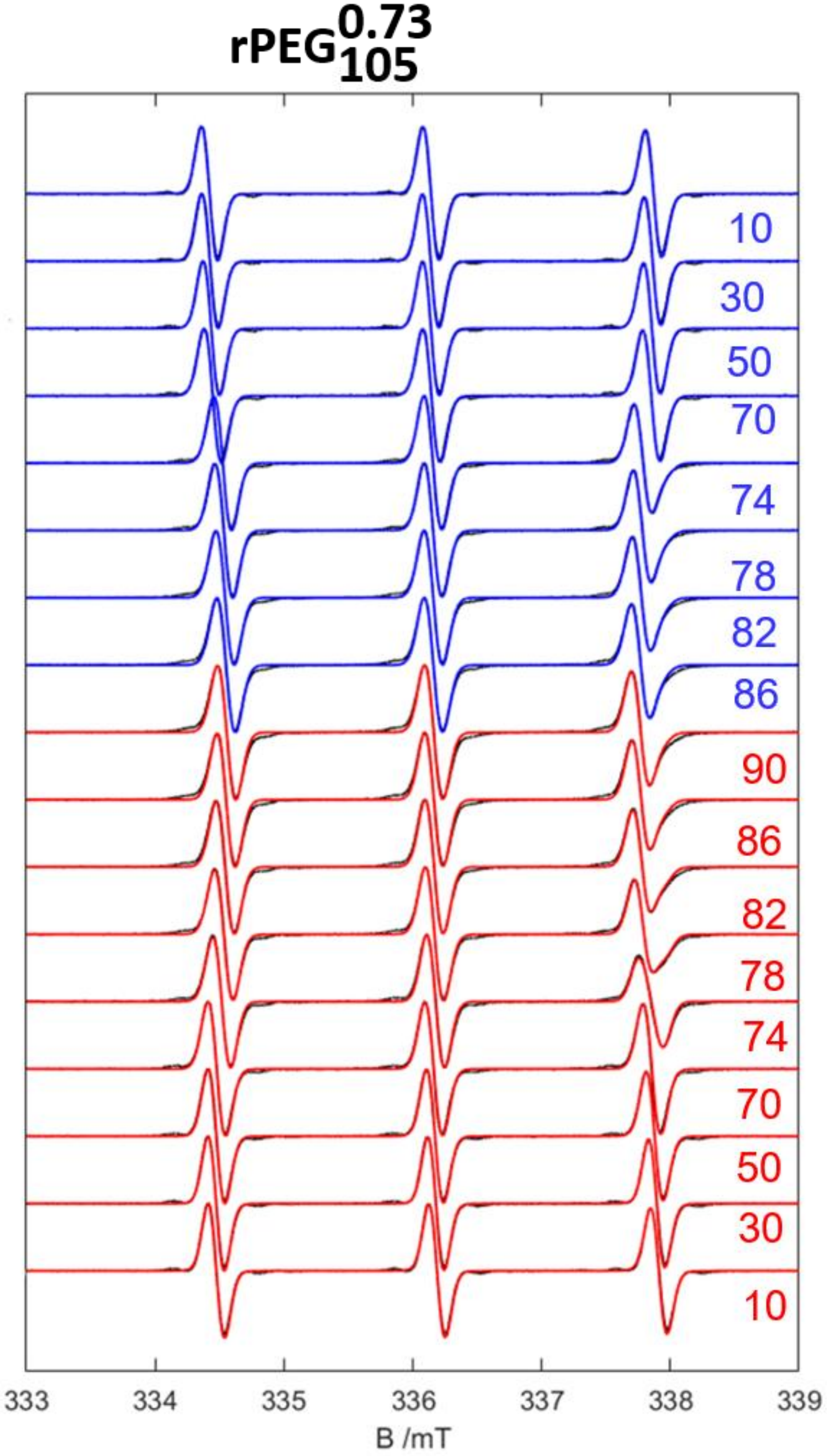

Figure S44. Experimental data (black) and corresponding simulation of EPR spectra of a 10 w% aqueous solution of rPEG$^{0.73}_{105}$ (1.00 mM TEMPO) for varying temperatures. Simulated spectra in heating and cooling cycles are shown in red and blue. For the purpose of clarity, only a selection of spectra is shown.

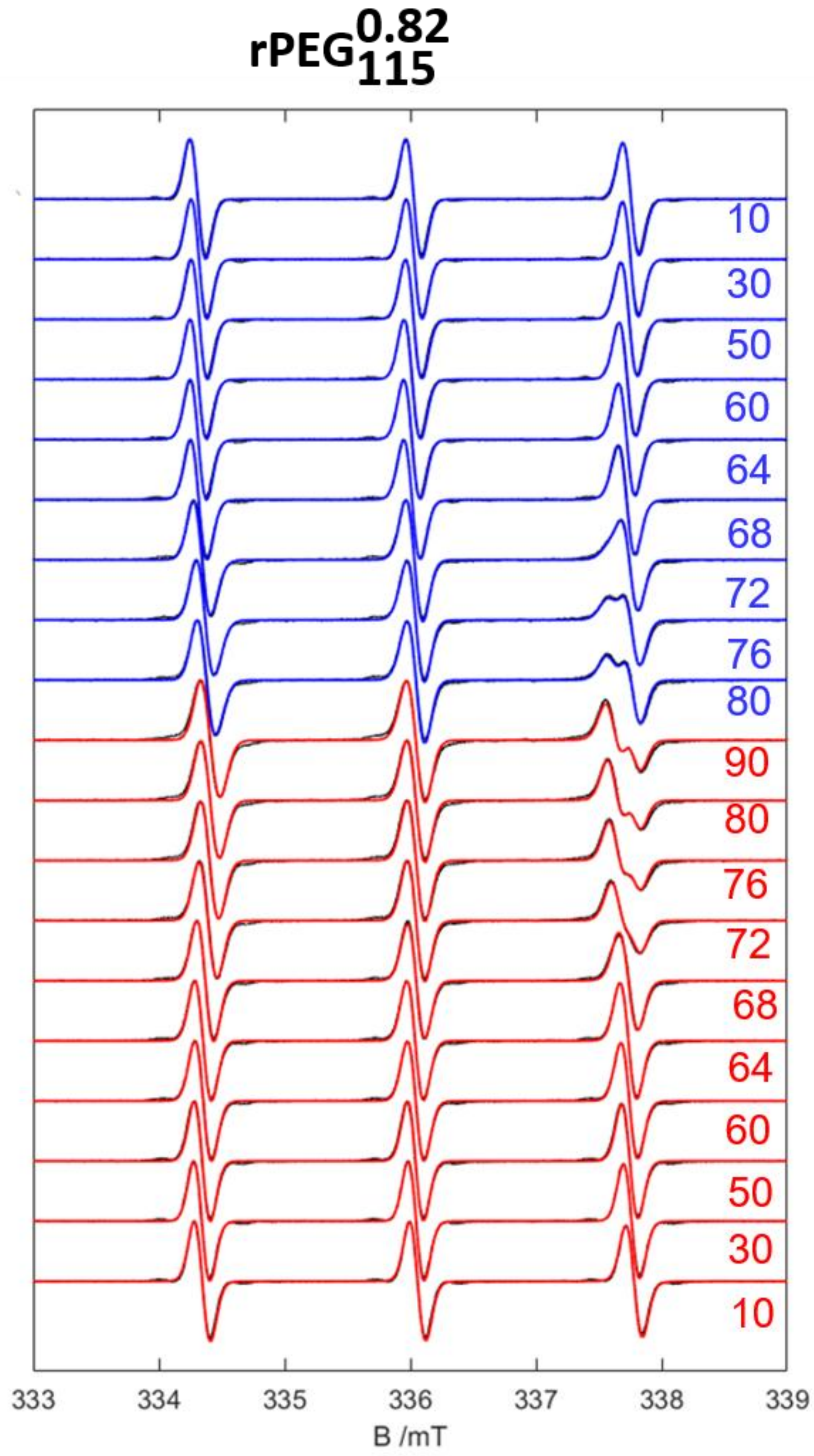

Figure S45. Experimental data (black) and corresponding simulation of EPR spectra of a 10 w% aqueous solution of rPEG$^{0.82}_{115}$ (1.00 mM TEMPO) for varying temperatures. Simulated spectra in heating and cooling cycles are shown in red and blue. For the purpose of clarity, only a selection of spectra is shown.

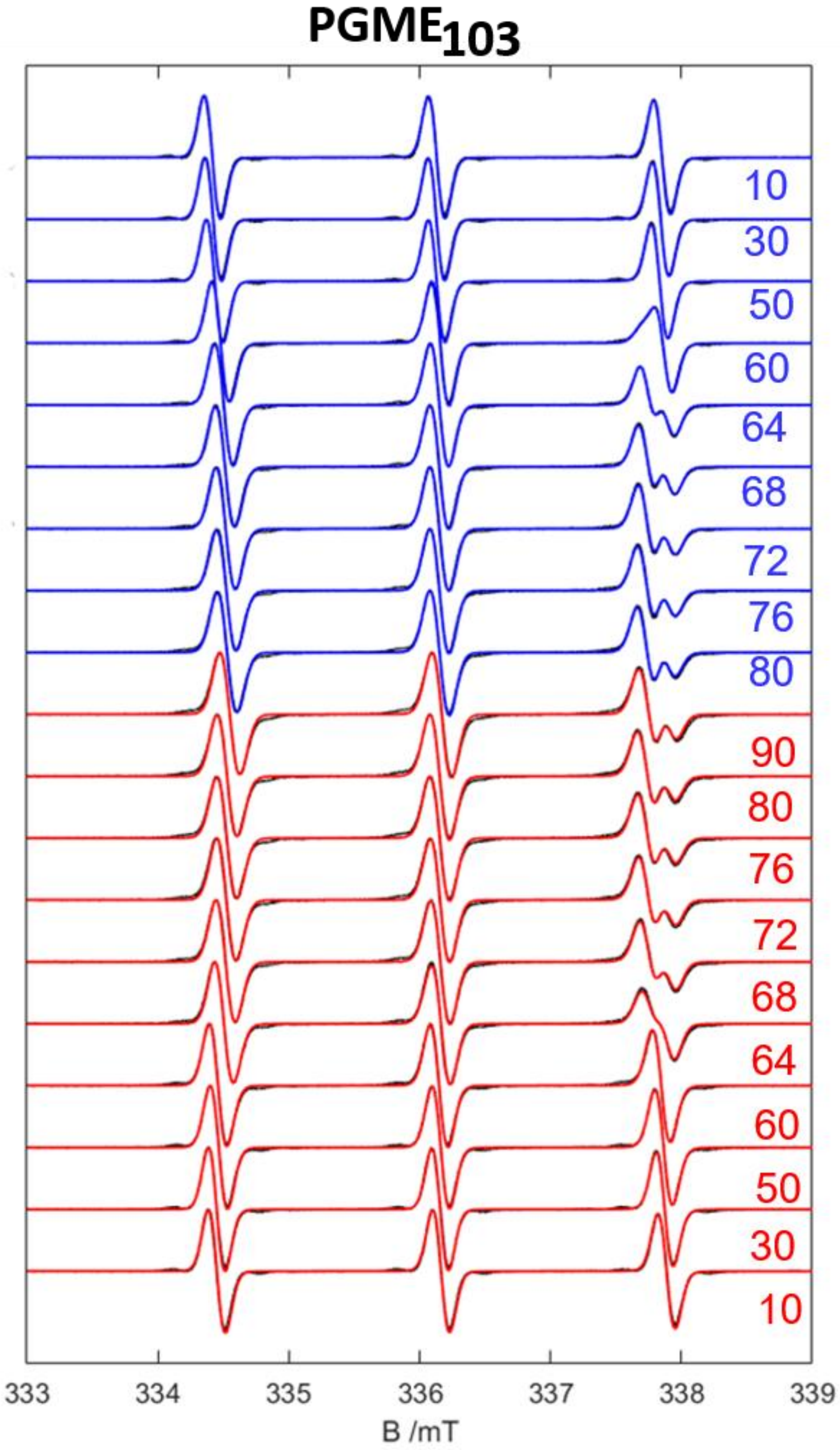

Figure S46. Experimental data (black) and corresponding simulation of EPR spectra of a 10 w% aqueous solution of $PGME_{103}$ (1.00 mM TEMPO) for varying temperatures. Simulated spectra in heating and cooling cycles are shown in red and blue. For the purpose of clarity, only a selection of spectra is shown.

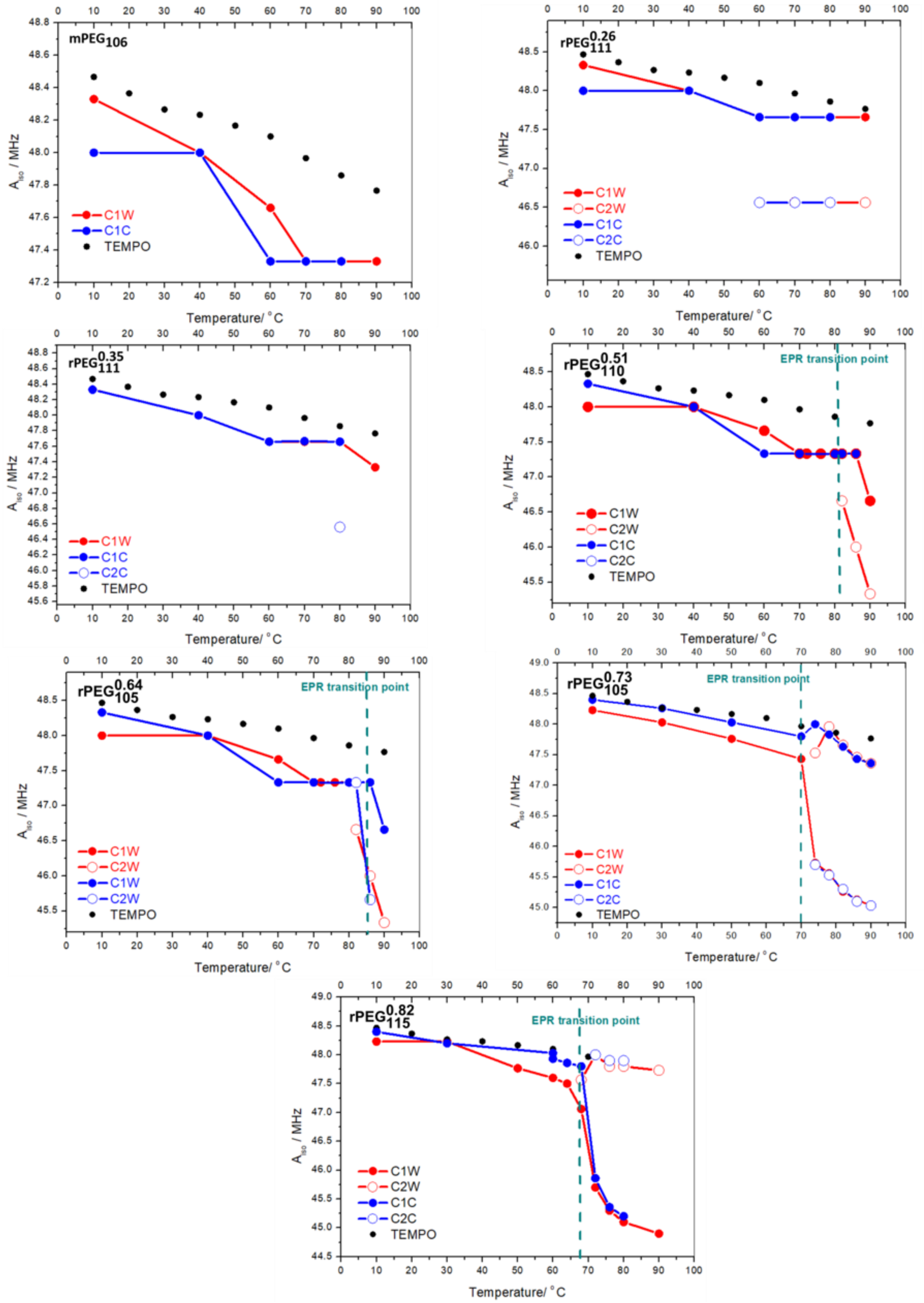

Figure S47. Change in local hydration (A_{iso} / MHz) of the polymer samples (with GME content 0, 26, 35, 51, 64, 73 and 81 mol%) due to temperature variation, as detected by EPR spectroscopy. The first and second components are abbreviated as C1 and C2. Simulated data for warming up (w) and cooling down (c) cycles are shown in red and blue curves. For the sake of comparison, isotropic hyperfine coupling of the spin probe (TEMPO) is shown as black points. EPR based transition temperatures are indicated by dashed lines**S**pectral hydrophobicity of all the measured Data points during heating and cooling cycles are shown in red and blue.

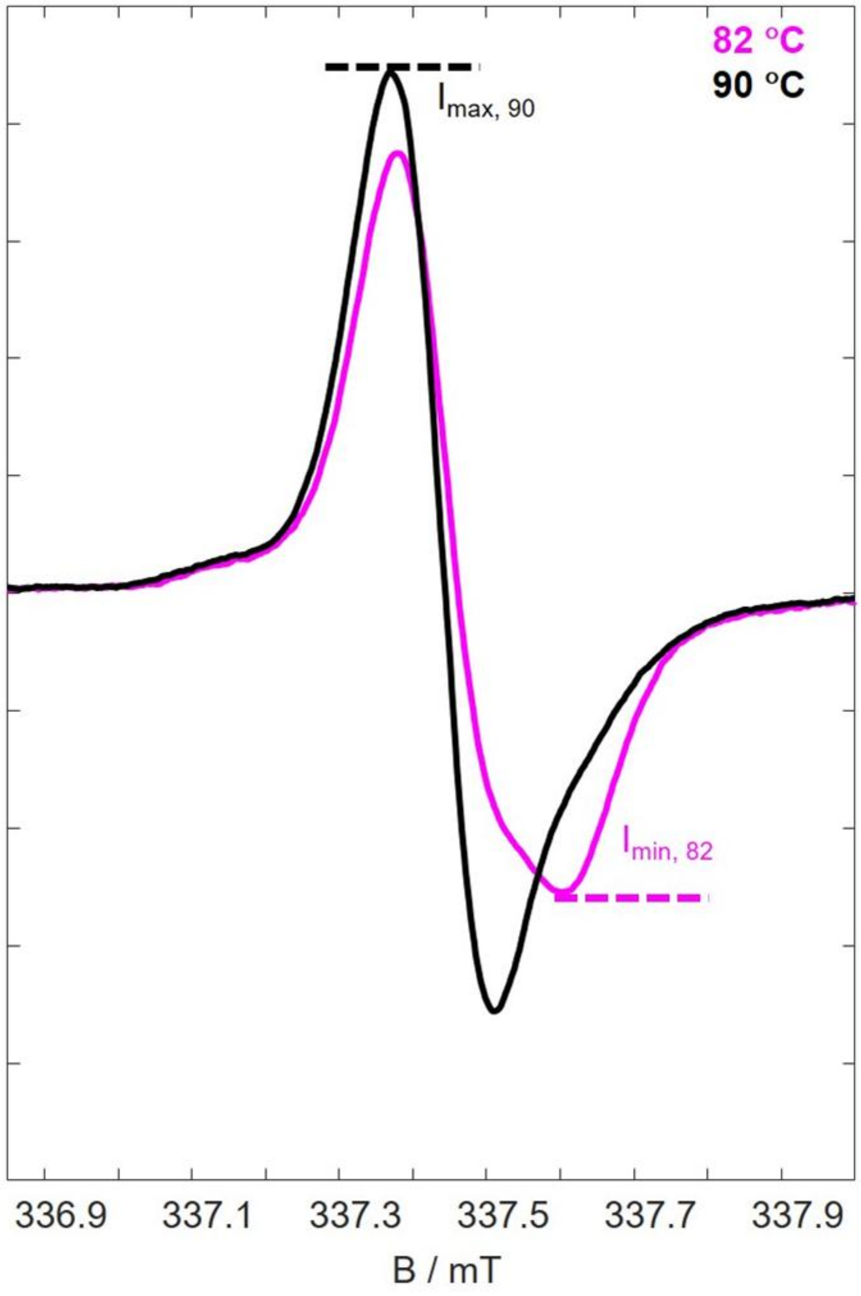

Figure S48. Reading spectral data to calculate overall hydrophobicity. $\boldsymbol{I_{max,90}}$ represents the intensity at the maximum of the high-field peak measured at 90 °C, and $\boldsymbol{I_{min,T}}$ is the intensity of the minimum of the "first component" (the hydrophilic component) in the high-field peak at measured temperature $\boldsymbol{T}$.

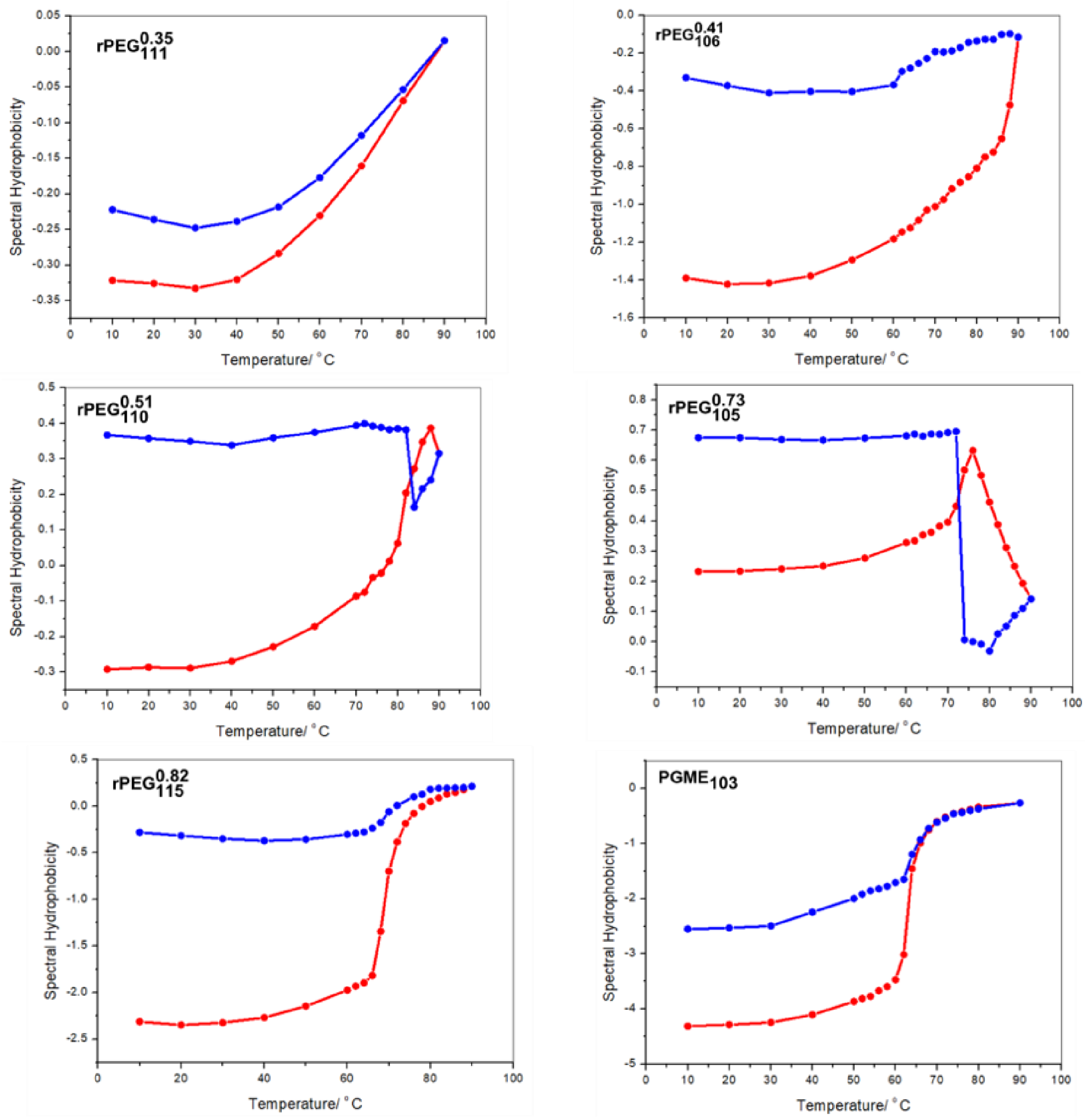

Figure S49. **S**pectral hydrophobicity of all the measured polymer samples with GME content 35, 41, 51, 73, 82, and 100 mol%. Data points during heating and cooling cycles are shown in red and blue.

8.2. Spin Hamiltonian parameters derived from simulations

$\boldsymbol{A_{iso}}$ and $\boldsymbol{g_{iso}}$ are the isotropic hyperfine coupling (in MHz) and *g*-value. The rotational correlation time ($\boldsymbol{\tau_c}$) is given in nanoseconds and spectral contribution of each component in spectrum in given as *% contribution*. Simulation data regarding cooling cycle are shown with index "C".

$$\tau_C = \frac{1}{6\sqrt[3]{D_{xx}D_{yy}D_{zz}}} \qquad \text{(eq S14)}$$

$\boldsymbol{A_{iso}}$ and $\boldsymbol{g_{iso}}$ are the trace of the ***A*** and ***g***-tensors, calculated as the following (eq S15, S16):

$$A_{iso} = \frac{A_{xx}+A_{yy}+A_{zz}}{3} \qquad \text{(eq S15)}$$

$$g_{iso} = \frac{g_{xx}+g_{yy}+g_{zz}}{3} \qquad \text{(eq S16)}$$

Table S3. Spin Hamiltonian parameters derived from spectral simulation of TEMPO spin probe-reference.

***T*/ °C**	***A****iso*/ MHz	$2A'_{zz}$ / MHz	τ_c / ns	g_{iso}	**% contribution**
**10**	48.4666	109.4	0.0056	2.0059	100
**20**	48.366	109.1	0.0081	2.0059	100
**30**	48.266	108.8	0.0081	2.0059	100
**40**	48.233	108.7	0.0056	2.0059	100
**50**	48.1666	108.5	0.0046	2.0059	100
**60**	48.1	108.3	0.0044	2.0059	100
**70**	47.966	107.9	0.0043	2.0059	100
**80**	47.86	107.6	0.0048	2.0059	100
**90**	47.766	107.3	0.0048	2.0059	100
**80-C**	47.86	107.6	0.0036	2.0059	100
**70-C**	47.95	107.85	0.0046	2.0059	100
**60-C**	48	108	0.0045	2.0059	100
**50-C**	48.1	108.3	0.0046	2.0059	100
**40-C**	48.2	108.6	0.0065	2.0059	100
**30-C**	48.3	108.9	0.0064	2.0059	100
**20-C**	48.366	109.1	0.0089	2.0059	100
**10-C**	48.433	109.3	0.0107	2.0059	100

Table S4. Spin Hamiltonian parameters derived from spectral simulation for $mPEG_{106}$.

***T*/ °C**	A_{iso} / MHz	$2A'_{zz}$ / MHz	τ_c / ns	g_{iso}	**% contribution**
**10**	48.33	108	0.0385	2.0059	100
**40**	48	107	0.0385	2.0059	100
**60**	47.66	106	0.0300	2.0059	100
**70**	47.33	105	0.0226	2.0059	100
**80**	47.33	105	0.0226	2.0059	100
**90**	47.33	105	0.0227	2.0059	100
**80-C**	47.33	105	0.0385	2.0059	100
**70-C**	47.33	105	0.0385	2.0059	100
**60-C**	47.33	105	0.0385	2.0059	100
**40-C**	48	107	0.0300	2.0059	100
**10-C**	48	107	0.0300	2.0059	100

Table S5. Spin Hamiltonian parameters derived from spectral simulation for $rPEG_{111}^{0.26}$.

T / °C	A_{iso} / MHz	$2A'_{zz}$ / MHz	τ_c / ns	g_{iso}	% contribution
**10**	48.33	108	0.030	2.0059	100
**40**	48	107	0.030	2.0059	100
**60**	47.66	106	0.030	2.0059	100
**70**	46.56<br>47.66	105<br>106	0.050<br>0.0260	2.0059	45<br>54
**80**	46.56<br>47.66	105<br>106	0.050<br>0.0057	2.0059	54<br>46
**90**	46.56<br>47.66	105<br>106	0.050<br>0.0057	2.0059	54<br>46
**80-C**	46.56<br>47.66	105<br>106	0.050<br>0.0057	2.0059	54<br>46
**70-C**	46.56<br>47.66	105<br>106	0.050<br>0.0057	2.0059	54<br>46
**60-C**	46.56<br>47.66	105<br>106	0.050<br>0.0057	2.0059	28<br>72
**40-C**	48	107	0.0350	2.0059	100
**10-C**	48	107	0.0350	2.0059	100

Table S6. Spin Hamiltonian parameters derived from spectral simulation for $rPEG_{111}^{0.35}$.

T/ °C	A_{iso}/ MHz	$2A'_{zz}$ / MHz	τ_c / ns	g_{iso}	% contribution
**10**	48.33	106	0.0300	2.0061	100
**40**	48	105	0.0300	2.0061	100
**60**	47.66	104	0.0300	2.0061	100
**70**	47.66	104	0.0300	2.0061	100
**80**	47.66	104	0.0300	2.0061	100
**90**	47.33	103	0.0300	2.0061	100
**80-C**	46.56<br>47.66	105<br>104	0.0500<br>0.0300	2.0059<br>2.0061	25<br>75
**70-C**	47.666	104	0.0300	2.0061	100
**60-C**	47.66	104	0.0300	2.0061	100
**40-C**	48	105	0.0300	2.0061	100
**10-C**	48.33	106	0.0300	2.0061	100

Table S7. Spin Hamiltonian parameters derived from spectral simulation for $rPEG_{106}^{0.41}$.

T/ °C	A_{iso}/ MHz	$2A'_{zz}$ / MHz	τ_c / ns	g_{iso}	**% contribution**
**10**	48.26	108.8	0.0138	2.0059	100
**30**	48.066	108.2	0.0079	2.0059	100
**50**	47.86	107.6	0.0063	2.0059	100
**70**	47.566	106.7	0.0054	2.0059	100
**80**	47.333	106.0	0.0053	2.0059	100
**86**	46.733	104.2	0.0630	2.0059	10
	47.333	106.0	0.0040	2.00589	90
**88**	46.733	104.2	0.0631	2.0059	40
	47.34	106.1	0.0040	2.00586	60
**90**	46.166	102.5	0.050	2.0059	37
	47.566	106.7	0.0030	2.00584	63
**88-C**	46.266	102.8	0.0501	2.0059	5
	47.533	106.6	0.0031	2.00588	95
**86-C**	47.533	106.6	0.0050	2.0059	100
**80-C**	47.6	106.8	0.0063	2.0059	100
**70-C**	47.766	107.3	0.0063	2.0059	100
**50-C**	47.966	107.9	0.0050	2.0059	100
**30-C**	48.2	108.6	0.0050	2.0059	100
**10-C**	48.366	109.1	0.0138	2.0059	100

Table S8. Spin Hamiltonian parameters derived from spectral simulation for $rPEG_{110}^{0.51}$.

T / °C	A_{iso} / MHz	$2A'_{zz}$ / MHz	τ_c / ns	g_{iso}	**% contribution**
**10**	48	107	0.0300	2.0059	100
**40**	48	107	0.0300	2.0059	100
**60**	47.66	107	0.0227	2.0059	100
**70**	47.333	106	0.0227	2.0059	100
**72**	47.333	106	0.0227	2.0059	100
**76**	47.333	106	0.0227	2.0059	100
**80**	47.333	106	0.0227	2.0059	100
**82**	46.66<br>47.333	103<br>106	0.0894<br>0.0300	2.0059<br>2.0059	40<br>60
**86**	46<br>47.333	101<br>106	0.0959<br>0.0300	2.0059<br>2.0059	60<br>40
**90**	45.333<br>46.66	101<br>104	0.2491<br>0.1053	2.0059<br>2.0059	50<br>50
**86-C**	45.66<br>47.333	101<br>106	0.0430<br>0.0300	2.0059<br>2.0059	60<br>40
**82-C**	47.333<br>47.33	107<br>106	0.0531<br>0.0300	2.0059<br>2.0059	40<br>60
**80-C**	47.33	106	0.0488	2.0059	100
**70-C**	47.333	105	0.0488	2.0059	100
**60-C**	47.333	106	0.0488	2.0059	100
**40-C**	48	106	0.0488	2.0059	100
**10-C**	48.33	106	0.0488	2.0059	100

Table S9. Spin Hamiltonian parameters derived from spectral simulation for $rPEG_{105}^{0.64}$.

T / °C	A_{iso} / MHz	$2A'_{zz}$ / MHz	τ_c / ns	g_{iso}	**% contribution**
**10**	48.33	107	0.0300	2.0059	100
**40**	48	106	0.0260	2.0059	100
**60**	47.66	105	0.0260	2.0059	100
**70**	47.33	104	0.0260	2.0059	100
**72**	47.33	104	0.1042	2.0059	100
**76**	46.66	104	0.1263	2.0059	100
**80**	46.33	103	0.1491	2.0059	100
**86**	45.33<br>46.33	101<br>103	0.0571<br>0.1133	2.0061<br>2.0057	59<br>41
**90**	45.33	98	0.0770	2.0059	100[a]
**86-C**	45.33<br>46.00	101<br>103	0.0494<br>0.2230	2.0061<br>2.0057	52<br>48
**80-C**	45.00<br>46.00	100<br>103	0.0426<br>0.4217	2.0061<br>2.0057	27<br>73
**76-C**	47.00	104	0.1263	2.0059	100
**72-C**	47.33	104	0.1042	2.0059	100
**70-C**	47.33	104	0.0260	2.0059	100
**60-C**	47.66	105	0.0260	2.0059	100
**40-C**	48.00	106	0.0260	2.0059	100
**10-C**	48.33	107	0.0300	2.0059	100

[a] entirely hydrophobic contribution

.

Table S10. Spin Hamiltonian parameters derived from spectral simulation for $rPEG_{105}^{0.73}$.

T / °C	A_{iso} / MHz	$2A'_{zz}$ / MHz	τ_c / ns	g_{iso}	**% contribution**
**10**	48.23	108.7	0.0126	2.0059	100
**30**	48.03	108.1	0.0079	2.0059	100
**50**	47.766	107.3	0.0069	2.0059	100
**70**	47.433	106.3	0.0054	2.0059	100
**74**	45.73<br>47.53	101.2<br>106.6	0.0100<br>0.0050	2.0059<br>2.00576	43<br>57
**78**	45.56<br>47.96	100.7<br>107.9	0.0100<br>0.0050	2.0059<br>2.0057166	77.5<br>22.5
**82**	45.266<br>47.666	99.8<br>107.0	0.0100<br>0.002511	2.0059<br>2.0057	83<br>17
**86**	45.133<br>47.46	99.4<br>106.4	0.0100<br>0.0025	2.0059<br>2.0057	87<br>13
**90**	45.033<br>47.366	99.1<br>106.1	0.0100<br>0.0025	2.0059<br>2.0057	90<br>10
**86-C**	45.1<br>47.43	99.3<br>106.3	0.0100<br>0.0025	2.0059<br>2.0057	90<br>10
**82-C**	45.3<br>47.633	99.9<br>106.9	0.0100<br>0.0025	2.0059<br>2.0057	90<br>10
**78-C**	45.533<br>47.833	100.6<br>107.5	0.0100<br>0.0025	2.0059<br>2.0057	89<br>11
**74-C**	45.7<br>48	101.1<br>108.0	0.0100<br>0.0031	2.0059<br>2.0057	87<br>13
**70-C**	47.8	107.4	0.0044	2.0059	100
**50-C**	48.033	108.1	0.0044	2.0059	100
**30-C**	48.26	108.8	0.0081	2.0059	100
**10-C**	48.4	109.2	0.0091	2.0059	100

Table S11. Spin Hamiltonian parameters derived from spectral simulation for $rPEG_{115}^{0.82}$.

T / °C	***A**_{iso}* / MHz	$2A'_{zz}$ / MHz	τ_c / ns	g_{iso}	**% contribution**
**10**	48.23	108	0.0134	2.0059	100
**30**	48.23	108	0.0089	2.0059	100
**50**	47.766	107.3	0.0075	2.0059	100
**60**	47.6	106.8	0.0061	2.0059	100
**64**	47.5	106.5	0.0061	2.0059	100
**68**	47.06<br>47.566	105.2<br>106.7	0.025<br>0.0061	2.0059<br>2.0057	62<br>38
**72**	45.7<br>48	101.1<br>108	0.025<br>0.0061	2.0059<br>2.0057	60<br>40
**76**	45.3<br>47.8	99.9<br>107.4	0.025<br>0.0061	2.0059<br>2.0057	62<br>38
**80**	45.1<br>47.8	99.3<br>107.4	0.025<br>0.0061	2.0059<br>2.005683	63<br>37
**90**	44.9<br>47.73	98.7<br>107.2	0.0501<br>0.0061	2.0059<br>2.0056	61<br>39
**80-C**	45.2<br>47.9	99.6<br>107.7	0.0501<br>0.0057	2.0059<br>2.0056	39<br>61
**76-C**	45.36<br>47.9	100.1<br>107.7	0.0501<br>0.0057	2.0059<br>2.00571	34<br>66
**72-C**	45.86<br>48	101.6<br>108	0.0501<br>0.0057	2.0059<br>2.0058	23<br>77
**68-C**	47.8	107.4	0.0234	2.0059	100
**64-C**	47.86	107.6	0.0144	2.0059	100
**60-C**	47.93	107.8	0.0107	2.0059	100
**50-C**	48.03	108.1	0.0079	2.0059	100
**30-C**	48.2	108.6	0.0072	2.0059	100
**10-C**	48.4	109.2	0.0123	2.0059	100

Table S12. Spin Hamiltonian parameters derived from spectral simulation for $PGME_{103}$.

T / °C	A_{iso} / MHz	$2A'_{zz}$ / MHz	τ_c / ns	g_{iso}	% contribution
**10**	48.233	108.7	0.0135	2.0059	100
**30**	48.066	108.2	0.0155	2.0059	100
**50**	47.733	107.2	0.0158	2.0059	100
**60**	46.5<br>47.63	103.5<br>106.9	0.0316<br>0.0158	2.0059<br>2.0059	8<br>92
**64**	45.466<br>47.8	100.4<br>107.4	0.0355<br>0.0141	2.0059<br>2.005683	51<br>49
**68**	45.166<br>47.966	99.5<br>107.9	0.0316<br>0.0126	2.0059<br>2.00565	62.9<br>37.1
**72**	44.96<br>47.9	98.9<br>107.7	0.0316<br>0.0126	2.0059<br>2.0056	66.5<br>32.5
**76**	44.83<br>47.9	98.5<br>107.7	0.0282<br>0.0112	2.0059<br>2.0056	67<br>33
**80**	44.8<br>47.9	98.4<br>107.7	0.0199<br>0.0112	2.0059<br>2.0056	68.8<br>31.2
**90**	44.63<br>47.66	97.9<br>107	0.0199<br>0.0112	2.0059<br>2.0056	68<br>32
**80-C**	44.733<br>47.83	98.2<br>107.5	0.0199<br>0.0126	2.0059<br>2.0056	68<br>32
**76-C**	44.84<br>47.9	98.5<br>107.7	0.0199<br>0.0126	2.0059<br>2.0056	66<br>34
**72-C**	44.96<br>47.93	98.9<br>107.8	0.0199<br>0.0126	2.0059<br>2.0056	65<br>35
**68-C**	45.033<br>47.93	99.1<br>107.8	0.0251<br>0.0126	2.0059<br>2.0056	63<br>37
**64-C**	45.266<br>47.9	99.8<br>107.7	0.0251<br>0.0126	2.0059<br>2.0056	58<br>42
**60-C**	45.4<br>47.666	100.2<br>107.0	0.0251<br>0.0158	2.0059<br>2.0057	22<br>78
**50-C**	47.733	107.2	0.0158	2.0059	100
**30-C**	48	108	0.0158	2.0059	100
**10-C**	48.23	108.7	0.0151	2.0059	100

9. Gas chromatography characterization of GME monomer

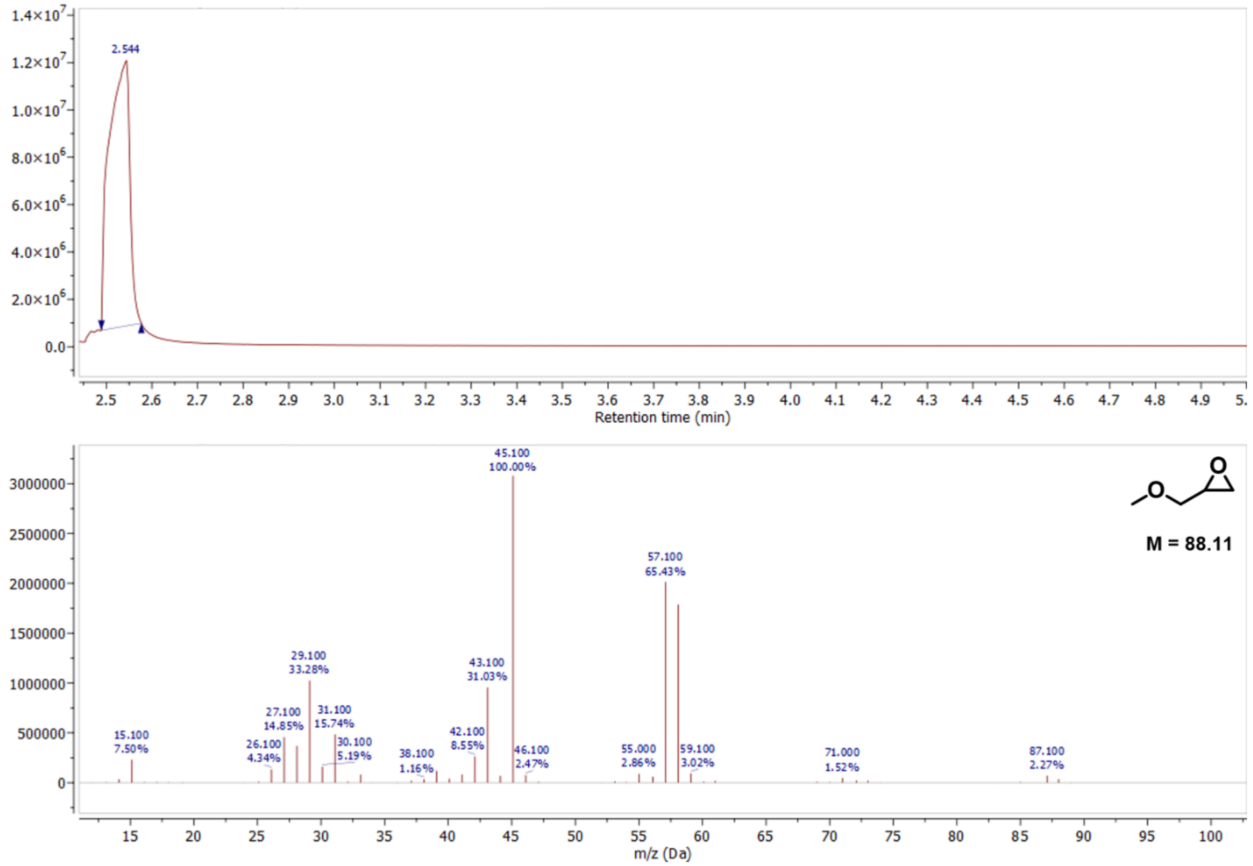

Figure S50. Top: Chromatogram of glycidyl methyl ether showing a retention time of 2.544 minutes with no other impurities found. Bottom: mass spectrum showing fragments of GME.

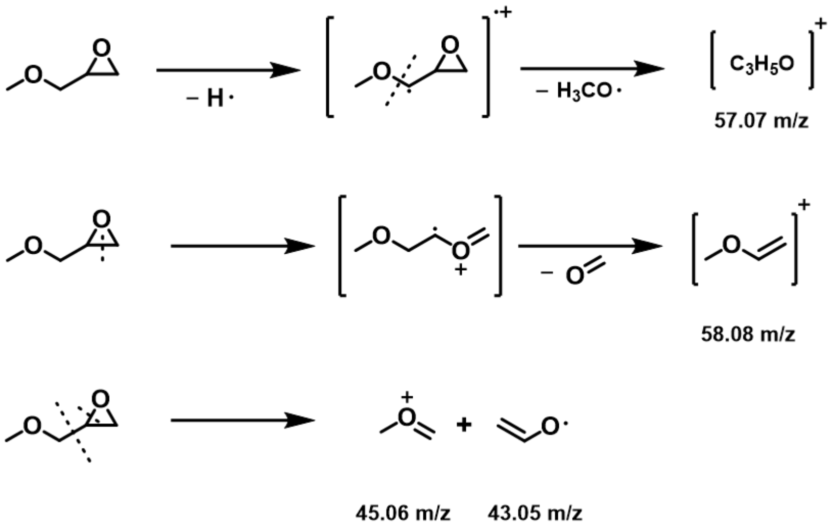

Scheme S3. Possible mass fragmentation of GME.

Supporting Information: References

(1) Han, S.; Hagiwara, M.; Ishizone, T. Synthesis of Thermally Sensitive Water-Soluble Polymethacrylates by Living Anionic Polymerizations of Oligo(ethylene glycol) Methyl Ether Methacrylates. *Macromolecules* 2003, *36* (22), 8312–8319. DOI: 10.1021/ma0347971.

(2) Aoshima, S.; Oda, H.; Kobayashi, E. Synthesis of thermally-induced phase separating polymer with well-defined polymer structure by living cationic polymerization. I. Synthesis of poly(vinyl ether)s with oxyethylene units in the pendant and its phase separation behavior in aqueous solution. *J. Polym. Sci., Part A: Polym. Chem.* 1992, *30* (11), 2407–2413. DOI: 10.1002/pola.1992.080301115.

(3) Junk, M. J. N.; Li, W.; Schlüter, A. D.; Wegner, G.; Spiess, H. W.; Zhang, A.; Hinderberger, D. Formation of a mesoscopic skin barrier in mesoglobules of thermoresponsive polymers. *J. Am. Chem. Soc.* 2011, *133* (28), 10832–10838. DOI: 10.1021/ja201217d.

(4) Kirschenbaum, L. J.; Riesz, P. Sonochemical degradation of cyclic nitroxides in aqueous solution. *Ultrason. Sonochem.* 2012, *19* (5), 1114–1119. DOI: 10.1016/j.ultsonch.2012.01.014.

(5) Stoll, S.; Schweiger, A. EasySpin, a comprehensive software package for spectral simulation and analysis in EPR. *J. Magn. Reson.* 2006, *178* (1), 42–55. DOI: 10.1016/j.jmr.2005.08.013.

(6) Schneider, D. J.; Freed, J. H. *Calculating Slow Motional Magnetic Resonance Spectra*. In: Berliner, L.J., Reuben, J. (eds) Spin Labeling. Biological Magnetic Resonance, vol 8. Springer, Boston, MA., Vol. 8. DOI: 10.1007/978-1-4613-0743-3_1.

(7) Nakatsuji, Y.; Nakamura, T.; Okahara, M.; Dishong, D. M.; Gokel, G. W. Crown cation complex effects. 22. Enhancement of cation binding in lariat ethers bearing a methyl group at the quaternary, pivot carbon atom. *J. Org. Chem.* 1983, *48* (8), 1237–1242. DOI: 10.1021/jo00156a018.

(8) Sigma-Aldrich. Safety Data Sheet 3-Chloro-1-methoxy-2-propanol for synthesis.

(9) Bakhtin, S.; Shved, E.; Bespalko, Y.; Tyurina, T.; Palchykov, V. Detailing the elementary stages in the oxirane ring opening reactions with carboxylic acids catalyzed by tertiary amines. *J. Phys. Org. Chem.* 2020, *33* (9). DOI: 10.1002/poc.4071.

(10) Reinicke, S.; Schmelz, J.; Lapp, A.; Karg, M.; Hellweg, T.; Schmalz, H. Smart hydrogels based on double responsive triblock terpolymers. *Soft Matter* 2009. DOI: 10.1039/b900539k.

(11) Dreier, P.; Matthes, R.; Fuß, F.; Schmidt, J.; Schulz, D.; Linden, G. M.; Barent, R. D.; Schüttner, S.; Neun, B. W.; Cedrone, E.; Dobrovolskaia, M. A.; Bros, M.; Frey, H. Isomerization of Poly(ethylene glycol): A Strategy for the Evasion of Anti-PEG Antibody Recognition. *J. Am. Chem. Soc.* 2025, *147* (25), 21538–21548. DOI: 10.1021/jacs.5c02716.

(12) Bae, Y. C.; Lambert, S. M.; Soane, D. S.; Prausnitz, J. M. Cloud-point curves of polymer solutions from thermooptical measurements. *Macromolecules* 1991, *24* (15), 4403–4407. DOI: 10.1021/ma00015a024.
